# Supplementary material for: Variable kinship patterns in Neolithic Anatolia revealed by ancient genomes
Source: Curr Biol. 2021 Jun 7;31(11):2455–2468.e18. doi: 10.1016/j.cub.2021.03.050 (PMC8210650; doi:10.1016/j.cub.2021.03.050)
Supplement: Document S1. Figures S1–S4 and Tables S1–S4 [file mmc1.pdf]

Current Biology, Volume *31*

Supplemental Information

Variable kinship patterns in Neolithic Anatolia revealed by ancient genomes

Reyhan Yaka, Igor Mapelli, Damla Kaptan, Ayça Doğu, Maciej Chyleński, Ömür Dilek Erdal, Dilek Koptekin, Kıvılcım Başak Vural, Alex Bayliss, Camilla Mazzucato, Evrim Fer, Sevim Seda Çokoğlu, Vendela Kempe Lagerholm, Maja Krzewińska, Cansu Karamurat, Hasan Can Gemici, Arda Sevkar, Nihan Dilşad Dağtaş, Gülşah Merve Kılınç, Donovan Adams, Arielle R. Munters, Ekin Sağlıcan, Marco Milella, Eline M.J. Schotsmans, Erinç Yurtman, Mehmet Çetin, Sevgi Yorulmaz, N. Ezgi Altınışık, Ayshin Ghalichi, Anna Juras, C. Can Bilgin, Torsten Günther, Jan Storå, Mattias Jakobsson, Maurice de Kleijn, Gökhan Mustafaoğlu, Andrew Fairbairn, Jessica Pearson, İnci Togan, Nurcan Kayacan, Arkadiusz Marciniak, Clark Spencer Larsen, Ian Hodder, Çiğdem Atakuman, Marin Pilloud, Elif Sürer, Fokke Gerritsen, Rana Özbal, Douglas Baird, Yılmaz Selim Erdal, Güneş Duru, Mihriban Özbaşaran, Scott D. Haddow, Christopher J. Knüsel, Anders Götherström, Füsun Özer, and Mehmet Somel

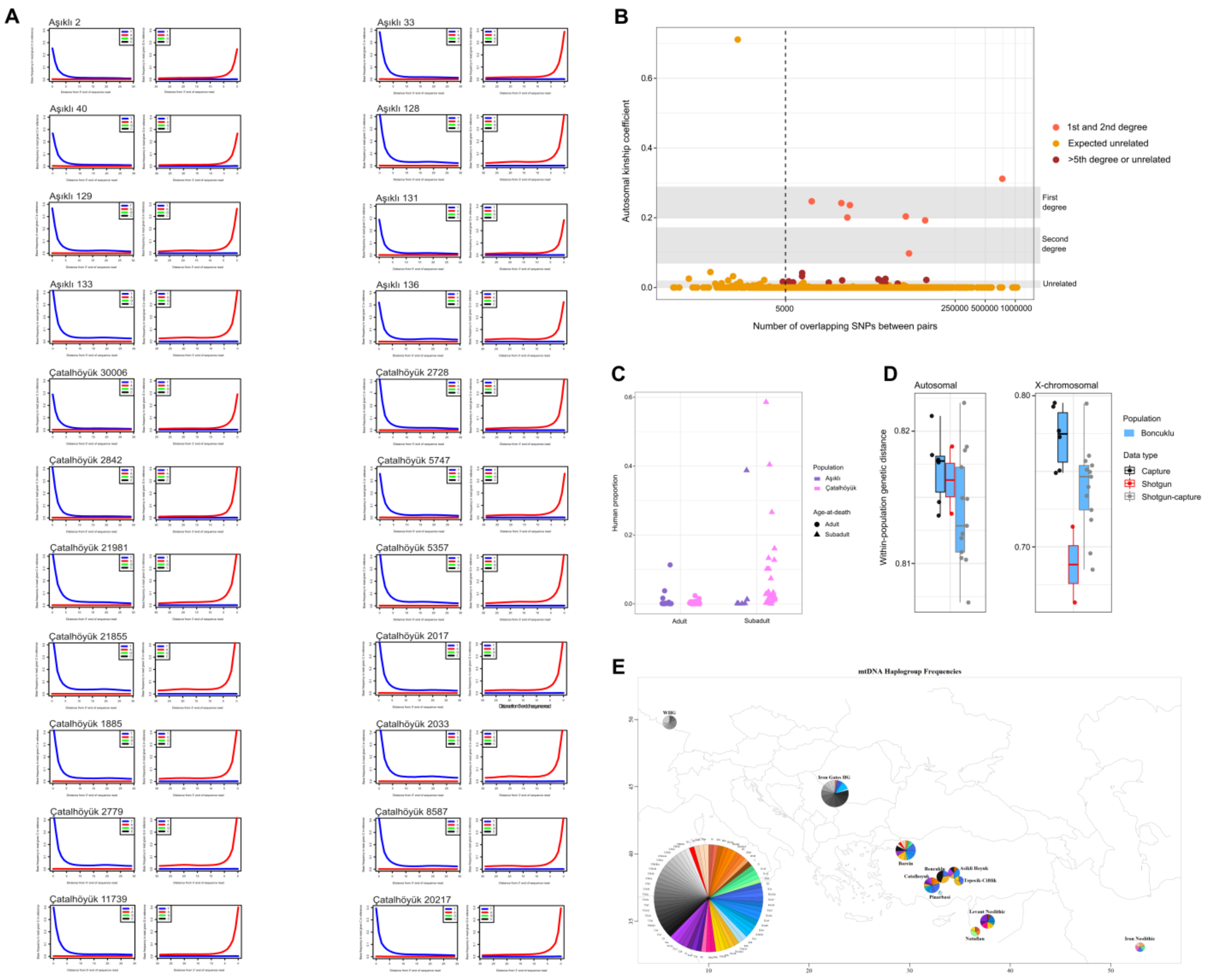

Figure S1. Postmortem damage (deamination) patterns, kinship coefficient (*θ*) estimates, endogenous human DNA proportions, technical influence on *f₃*-statistic-based genetic distances and mitochondrial DNA haplogroups, Related to Table 1, Figure 2 and STAR Methods. (A) The graphs show postmortem damage patterns (*PMD*) of all samples from Aşıklı (*n*=8) and Çatalhöyük (*n*=14) as measured by the frequency of transitions at the first 30 positions at the 5' (cytosine to thymine transitions) and 3'ends (guanine to adenine transitions) of the reads. The x-axis indicates the distance from the 5' and 3'ends of the reads and the y-axis indicates the frequency of the *PMDs* (Table Z1). **(B)** Kinship coefficient (*θ*) estimates vs. overlapping SNP numbers between n=1711 pairs of n=59 Anatolian Neolithic individuals. The x-axis shows the number of overlapping autosomal SNPs between a pair of ancient genomes, and the y-axis shows kinship coefficient estimates for pairs of individuals calculated with autosomal genomic data using *NgsRelate* software (Table Z11). The horizontal gray bars indicate expected *θ* values based on simulation for first-degree related, second-degree related, and unrelated pairs, from top to bottom, calculated with *NgsRelate* software using haploid data (STAR Methods). The orange points are pairs expected to be unrelated based on their temporal and spatial distances, pink points are those pairs identified as 1st and 2nd degree related from the same site and period, while dark red points are those pairs identified as distantly (>5th degree) or unrelated from the same site and period. The pair with a *θ* estimate of >0.7 is Aşıklı 2-Aşıklı 40, with c.300 years between them. Based on this result, in order to avoid false positives, a minimum

5,000 overlapping SNPs was chosen as a conservative threshold to assess genetic kinship between pairs. **(C)** Endogenous human DNA proportions in Aşıklı (n=30) and Çatalhöyük (n=60) individuals. Endogenous human DNA proportions in Aşıklı (purple) and Çatalhöyük (pink) individuals' remains were measured as the percentage of shotgun sequenced DNA molecules from prescreening experiments mapping to the reference human genome. Each individual is represented by a single observation. The highest proportion was used for a minority of cases where multiple libraries had been produced per individual. Adult and subadult categories were assigned using osteological age-at-death estimates by the anthropology teams, using standard methods such as human growth, dental calcification and bone maturity [S1]. Endogenous DNA proportions show significant difference between subadult and adult categories in Çatalhöyük (two sided Mann-Whitney U test, $P<0.0001$), but not in Aşıklı ($P=0.70$). **(D)** Technical influence on f_3-statistic-based genetic distances. Distributions of pairwise genetic distances based on outgroup f_3-statistics ($1-f_3$) among Boncuklu individuals, plotted depending on data type, for autosomal SNPs (left) and for X chromosomal SNPs (right). "Capture" indicates pairs of individuals where data were generated using the 1240K SNP capture technology [S2], while "shotgun" indicates pairs of individuals where data were generated using shotgun sequencing enriched with whole genome capture probes [3]; "shotgun-capture" indicates pairs of individuals where one of each pair was produced using either procedure. Although the impact of technical differences is modest for autosomal data, in the comparisons involving X chromosomal SNPs the f_3-statistics-based distance estimates behave differently between the capture and shotgun datasets (despite the overall genetic homogeneity of the Boncuklu population), suggesting the influence of technical effects. **(E)** The geographical distribution of mitochondrial DNA haplogroups of the ancient individuals used in this study. The map displays mitochondrial haplogroup frequencies identified in the early Holocene populations listed in Tables Z1 and Z3. Shades of the same color represent haplogroups categorized under the same major haplogroup, as indicated in the key. Size of the pies are proportional to the sample size. Pie charts were plotted based on the approximate coordinates of archaeological sites and regions. WHG: Western European hunter-gatherers. The map was drawn using the *mapplots* package version 1.5.1 [4] in *R*.

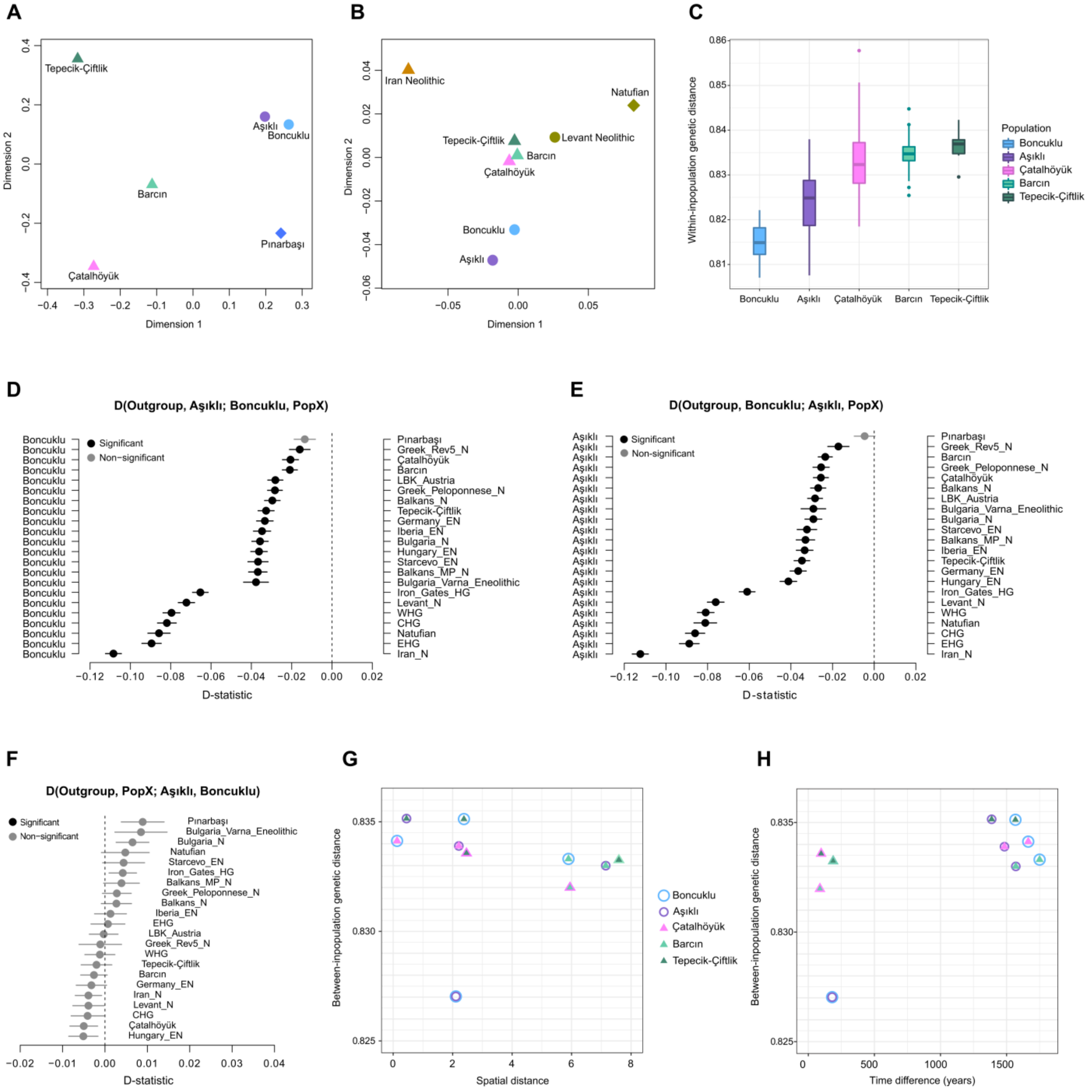

Figure S2. Genome-wide similarity patterns among ancient populations, Related to Figure 1. (**A**) Population level multidimensional scaling (MDS) plot summarizing genetic distances based on outgroup f_3-statistics calculated between populations from Anatolia using population genotype data (goodness of fit r^2=0.8). (**B**) Population level MDS plot summarizing genetic distances based on F_{ST} calculated between populations from Anatolia, Levant and Iran using population genotype data (goodness of fit r^2=0.95). **(C)** Distribution of within-population genetic distances for each population based on outgroup f_3-statistics. The whiskers extend to the most extreme data point ≤1.5 times the interquartile range. (**D**) Population level *D*-statistics for the topology *D(Yoruba, Aşıklı; Boncuklu, PopX)* where Boncuklu is indicated on the left-hand y-axis and *PopX* stands for ancient individual/populations from Anatolian, Near Eastern, Balkans and European farmer and hunter-gatherer groups, indicated on the right-hand y-axis. $D < 0$ shows higher genetic affinity between the test population (name indicated on the top, Aşıklı) and

Boncuklu, and $D > 0$ shows higher genetic affinity between the test population and the second individual/population as *PopX*. In each comparison, black color indicates nominally significant *D*-statistics (*i.e.,* not corrected for multiple testing) with $|Z| \geq 3$ and grey-color shows non-significant values. Horizontal lines show +/- one standard error. See Table Z3 for population details. (**E**) Population level *D*-statistic for the topology *D(Yoruba, Boncuklu; Aşıklı, PopX)* where Aşıklı is indicated on the left-hand y-axis and *PopX* stands for ancient individual/populations from Anatolian, Near Eastern, Balkans and European farmer and hunter-gatherer groups, indicated on the right-hand y-axis. $D < 0$ shows higher genetic affinity between the test population (name indicated on the top, Boncuklu) and Aşıklı, and $D > 0$ shows higher genetic affinity between the test population and the second individual/population as *PopX*. See Table Z3 for population details. (**F**) Population level *D*-statistics for the topology *D(Yoruba, PopX; Aşıklı, Boncuklu)* where Aşıklı and Boncuklu are indicated on the left- and right-hand y-axis, respectively, and *PopX* stands for ancient individual/populations from Anatolian, Near Eastern, Balkans and European farmer and hunter-gatherer groups, indicated in the middle. $D < 0$ shows higher genetic affinity between the test population (*PopX*) and Aşıklı, and $D > 0$ shows higher genetic affinity between the test population (*PopX*) and Boncuklu. See Table Z3 for population details. (**G-H**) Genetic distance between populations compared with their spatial and chronological differences. The plots show genetic distance vs. spatial distance (**G**) and genetic distance vs. time difference (**H**) between pairs of Neolithic Anatolian settlements. Each pair is denoted by the combination of symbols of both sites, shown in the key. For each pair of sites, a genetic distance matrix was created based on outgroup f_3-statistics using $(1\text{-}f_3)$. Matrices of spatial distance and time distance between the same pairs were calculated as the Euclidean distance between their longitude and latitude, and the difference between the ages of the settlements (mid-point of the occupation period), respectively. Overall, both time and space explain non-significant genetic variation among across these five sites (one-sided Mantel test $P>0.8$ and $P>0.4$, respectively).

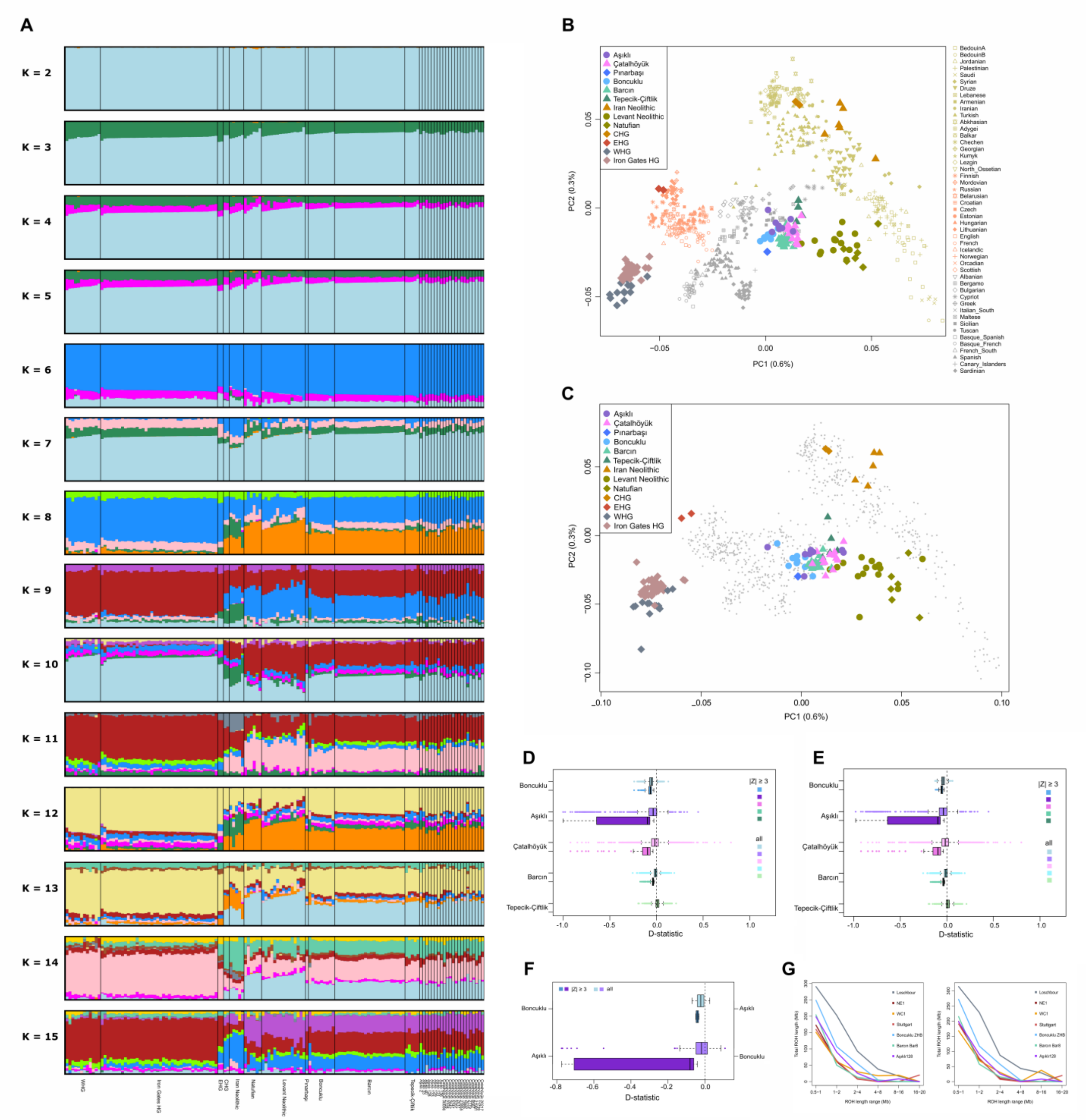

Figure S3. *ADMIXTURE* analysis, principal components analysis (PCA), individual-level *D*-statistics results, and runs of homozygosity (ROH), Related to Figure 1. (A) *ADMIXTURE* analysis results for *K*=2 to *K*=15 ancestry components for ancient individuals are shown as bar charts. The individuals included in each population sample are listed in Tables Z2 and Z3. We performed unsupervised *ADMIXTURE* analysis using n=231 present-day populations from the Human Origins dataset [5,6] and ancient individuals were projected on the calculated components (STAR Methods). **(B)** PCA with ancient individuals from Anatolia including published ancient and present-day individuals from West Eurasian populations. The individuals included in each population sample are listed in Tables Z2 and Z3. Each ancient individual's genotype was projected upon the first two principal components calculated using n=49 West Eurasian present-day populations from the Human Origins dataset [5,6] (the percentages of

variance explained are shown in parentheses). Colored dots represent different ancient and modern-day individuals as shown in the key. **(C)** PCA plot describing genetic similarities as in panel B, but using only n=110,933 transversion SNPs and n=49 West Eurasian present-day populations from the Human Origins dataset [5,6]. Also, for simplicity we did not color present-day populations in this figure. **(D)** Distributions of individual-level *D*-statistics involving comparisons among and within Anatolian Neolithic settlements. Negative *D*-statistics indicate an individual was genetically closer to another from her/his own settlement (shown on the left-hand side labels), than to an individual from one of the other four settlements, in *D*-tests of the form *D(Outgroup, X1; X2, X3)*, where *X1* and *X2* are from the same, and *X3* from a different Anatolian Neolithic settlement. Test results that are nominally significant ($|Z|>3$, darker colors) and all results (lighter colors) are shown as separate boxplots. In total, 22,638 *D*-tests were conducted and 84-93% of 576-11,780 comparisons per site were significant, except for Tepecik-Çiftlik. In the latter, none of the test results were nominally significant. In all analyses we removed all but one individual where clusters of genetic relatives were identified. **(E)** Distributions of individual-level *D*-statistics as in panel D, but only using shotgun data for Boncuklu, to avoid the influence of technical biases that may arise due to use of capture and shotgun data. The results are qualitatively the same as in panel D. **(F)** Distributions of individual-level *D*-statistics as in panel D for Aşıklı vs Boncuklu comparisons only using shotgun data for Boncuklu to avoid the influence of technical biases that may arise due to use of capture and shotgun data. In total, 156 *D*-tests were conducted and 11-13% of 36-120 comparisons per site were significant. **(G)** Runs of homozygosity (ROH) in seven ancient early Holocene West Eurasian genomes. The left panel shows ROH length distributions for Loschbour (European Mesolithic), NE1 (Hungary Neolithic), WC1 (Iran Neolithic), Stuttgart (early European Neolithic), Boncuklu ZHB (Bon002) (Anatolian Aceramic), Barcın Bar8 (M10-106) (Anatolian Ceramic Neolithic), Aşıklı 128 (Anatolian Aceramic) and in the right panel ROH length distributions with each genome subsampled down to 5× coverage. Estimated F_{ROH} values per genome are presented in Table Z15.

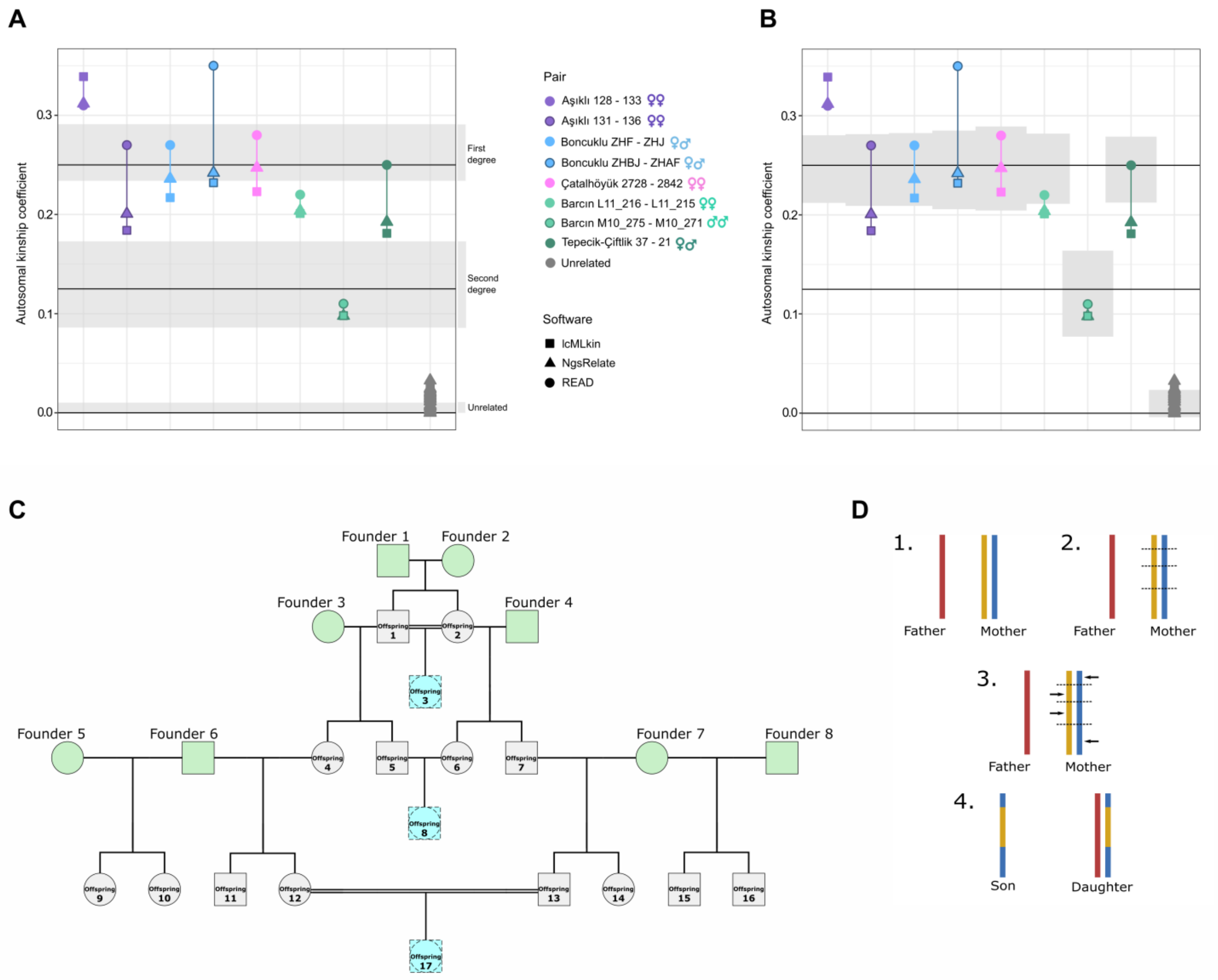

Figure S4. Kinship coefficients (θ), the pedigree used in kinship simulations and simulation of X chromosomal meiosis, Related to Figure 2. (A-B) Kinship coefficients (θ) between related pairs of individuals calculated using autosomal genomic data and three different software, shown as different symbols. The horizontal black lines indicate expected autosomal θ values for first-degree related, second-degree related, and unrelated pairs (0.25, 0.125 and 0, respectively). The horizontal gray bars indicate expected θ values for first-degree related, second-degree related, and unrelated pairs, from top to bottom, calculated with *NgsRelate* software on one hundred simulated pedigrees, which resulted in 2,000 samples for each group (STAR Methods). Whereas first-, second- and third- degree ranges were defined by the 0.025 and 0.975 quantiles, the unrelated group had an upper boundary identified by the 0.95 quantile, as we considered zero to be its lower limit. The left panel (**A**) shows the ranges for diploid data with 5,000 SNPs – [0.2357, 0.2882], [0.0919, 0.1681], [0.0304, 0.1187], [0.0000, 0.0095] for first-, second-, third- (not in the figure) degrees and unrelated pairs respectively (the difference from Figure 2A is that the latter shows ranges calculated using pseudo-haploidized data) (Table Z16). In the right panel (**B**) the ranges were determined using haploidized data and the same number of SNPs each pair shared, i.e., 157,172 SNPs for Aşıklı 128-133 resulting in a first-degree range of [0.2201, 0.2760], 20,810 for Aşıklı 131-136 and an interval for the first degree equal to [0.2155, 0.2765], 22,076 for Boncuklu ZHF-ZHJ resulting in a first-degree range of [0.2142, 0.2790], 18,136 for Boncuklu ZHBJ-ZHAF for an estimated first-degree interval equal to [0.2148, 0.2771], 9,151 for

Çatalhöyük 2728-2842 and first-degree range of [0.2118, 0.2827], 80,115 for Barcın L11_216-L11_215 producing a first-degree interval of [0.2197, 0.2763], 86,247 for Barcın M10_275-M10_271 and a second-degree range of [0.0852, 0.1586], 125,110 for Tepecik-Çiftlik 37-21 with a first-degree range of [0.2204, 0.2742]. For the unrelated group we considered 5,000 SNPs which resulted in an interval of [0.0000, 0.0210] (Table Z16). Since Aşıklı 128-133 shared 741,193 SNPs, we used the full set of 157,172 SNPs for this Aşıklı pair. **(C)** The light green-colored elements (delimited by a solid line) represent the founders who were randomly selected from a genomic polymorphism dataset of unrelated modern Tuscany individuals from 1000 Genomes phase 3 dataset [7]. All individuals in the pedigree have a predefined sex (circle for female and square for male), the only exception being the inbred offspring (light blue-colored elements surrounded by dashed lines) whose sex was uniformly randomized across the generated pedigrees. (**D**) The X chromosome recombination process in the mother but not in the father. A pair of X chromosomes in the mother (**1**) are each randomly broken at 3 recombination (crossover) points (**2**), and the resulting 4 segments are randomly switched between each pair of X chromosomes (**3**). A son solely inherits one of the resulting mother's recombinant gamete, whereas a daughter inherits also the original X chromosome of the father (**4**).

Site	Individuals studied	Contemporaneous pairs	Co-buried pairs	First (second) degree related pairs	Kin pairs among co-burials %	Co-buried individuals	Genetic kin	Kin among co-burials %
Aşıklı	8	10	10	2<br>(0)	20<br>(2/10)	5	4	80<br>(4/5)
Boncuklu	9	36	10	2<br>(0)	20<br>(2/10)	5	4	80<br>(4/5)
Çatalhöyük	14	66	17	1<br>(0)	6<br>(1/17)	10	2	20<br>(2/10)
Barcın	23	107	12	1<br>(1)	16<br>(2/12)	10	4	40<br>(4/10)
Tepecik-Çiftlik	5	4	1	1<br>(0)	100<br>(1/1)	2	2	100<br>(2/2)
TOTAL	59	223	50	7<br>(1)	-	32	16	-

Table S1. Frequencies of individuals and individual pairs studied for genetic kinship, Related to Figure 2 and STAR Methods.
The column titled "Contemporaneous pairs" shows the numbers of all pairs of individuals from the same or similar archaeological levels and thus could be potentially closely related. "Co-buried pairs" shows the number of pairs of individuals in co-burial clusters, associated with (*i.e.,* buried inside or next to) the same or proximate buildings, shown in detail in Figure 3. "First (second) degree related pairs" shows the number of first-degree (and in parentheses, second-degree) related pairs identified in this study. "Kin pairs among co-burials %" shows the percentage of co-buried pairs identified as genetic kin, i.e. first- or second-degree (and the actual numbers in parentheses). "Co-buried individuals" shows the number of individuals' whose remains were associated with a building that included at least one other individual genetically studied here. "Kin among co-burials %" shows the percentage of co-buried individuals with a first- or second-degree relative identified in this study (and the actual numbers in parentheses). All genetic kin identified were individuals who were part of co-burials clusters. No pairs with third degree relatedness were identified here. A Fisher's exact test performed by comparing the frequencies of individuals in co-burials with relatives in Aşıklı and Boncuklu (n=8) versus Çatalhöyük and Barcın Höyük (n=6), and without relatives in the same pairs of sites (n=2 and n=14, respectively), reveals a significant difference (odds ratio=8.6, Fisher's exact test P=0.019). When including Tepecik-Çiftlik data to the Ceramic Neolithic group, we also find a marginal difference between Aceramic and Ceramic period frequencies (odds ratio=6.6, Fisher's exact test P=0.054).

**Kin-relationship**	**Autosomal θ**	**chrX θ**	k_0	k_1	k_2
Mother-daughter	0.25	0.25	0	1	0
Mother-son	0.25	0.5	0	1	0
Father-daughter	0.25	0.5	0	1	0
Father-son	0.25	0	0	1	0
Sisters	0.25	0.375	0.25	0.5	0.25
Brothers	0.25	0.5	0.25	0.5	0.25
Brother-sister	0.25	0.25	0.25	0.5	0.25

Table S2. Expected autosomal and X chromosomal (chrX) kinship coefficients (θ) and Cotterman coefficients for first-degree genetic relatives, Related to Figure 2 and STAR Methods.
Cotterman coefficients (k_0, k_1, k_2) define probabilities of sharing 0, 1 or 2 alleles identical-by-descent between a pair of individuals. The values assume no inbreeding in the pedigree.

**Pair**	**Most likely pedigree relationship**	**Autosomal θ**	**chr X θ**	**Autosomal Cotterman coefficients**			**MtDNA haplogroup**	**chrY haplogroup**	**Osteological age-at-death**	**No. of SNPs**	**C14 date (95% cal BCE)**
				k_0	k_1	k_2					
Aşıklı 128-133	Sisters	0.31	0.25	0.15	0.45	0.40	Same	-	Child and old adult	741,193	8225–7955<br>8170-7735
	We inferred this pair as likely sisters (as opposed to mother-daughter) due to their low k_1 values, although their low X chromosomal θ could also be consistent with a mother-daughter pair. Notably, the Çatalhöyük and Barcın sister pairs also display similar statistics. They may have been contemporary based on their radiocarbon data.										
Aşıklı 131-136	Sisters	0.20	0.17	0.46	0.28	0.25	Same	-	Child and adult	20,810	8200-7740<br>8175-7655
	We inferred this pair as likely sisters (as opposed to mother-daughter) due to their low k_1 values, although their low X chromosomal θ could also be consistent with a mother-daughter pair. Notably, the Çatalhöyük and Barcın sister pairs also display similar statistics. They may have been contemporary based on their radiocarbon data.										
Boncuklu ZHJ-ZHF	Mother-son	0.24	0.43	0.22	0.62	0.13	Same	-	Old adult and adult	22,076	8295-8240<br>8225-7940
	We inferred this pair as parent-offspring (as opposed to brother-sister) due to their relatively high X chromosomal θ, their relatively high autosomal k_1 values and their age-at-death and C14 dates. A mother-son relationship is more likely than a father-daughter relationship given mtDNA haplogroup sharing (Table Z14) and their age-at-death and C14 dates (Table Z2). Their radiocarbon data suggests that it is 90% probable that the woman (ZHJ) died first.										
Boncuklu ZHBJ-ZHAF	Brother-sister	0.24	0.28	0.18	0.67	0.10	Same	-	Middle adults	18,136	>7952<br>8285-8010
	We inferred this pair as likely brother-sister (as opposed to parent-offspring) due to their low X chromosomal θ, but their relatively high autosomal k_1 could also be consistent with a mother-son pair. A father-daughter pair is also possible but even less likely due to mtDNA haplogroup sharing. They may have been contemporary based on their radiocarbon data.										
Çatalhöyük 2728-2842	Sisters	0.25	0.23	0.49	0.03	0.48	Same	-	Infant and child	9,151	6695-6505<br>6690-6505
	We inferred this pair as sisters (as opposed to mother-daughter) due to their subadult age. Their radiocarbon data also suggests they may have been contemporary.										

<table>
<tr><td rowspan="2">Barcın L11_216-L11_215</td><td>Sisters</td><td>0.20</td><td>0.28</td><td>0.37</td><td>0.44</td><td>0.19</td><td>Same</td><td>-</td><td>Both infants</td><td>80,115</td><td>c.6200-6100
6320-6080</td></tr>
<tr><td colspan="11">We inferred this pair as sisters (as opposed to mother-daughter) due to their subadult age. Their radiocarbon data also suggests they may have been contemporary.</td></tr>
<tr><td rowspan="2">Barcın M10_275 - M10_271</td><td>Paternal half-siblings or uncle-nephew</td><td>0.1</td><td>0.02</td><td>0.61</td><td>0.39</td><td>0.0</td><td>Different</td><td>Same</td><td>Both infants</td><td>86,247</td><td>6405-6240
6425-6250</td></tr>
<tr><td colspan="11">We inferred this pair as paternal half-siblings or an uncle-nephew pair (as opposed to maternal half-siblings or a grandparent-grandchild pair) due to their sharing of Y chromosomal but not mtDNA haplogroups and their young age. A third-degree relationship (paternal cousins) is also possible. Their radiocarbon data suggests they may have been contemporary.</td></tr>
<tr><td rowspan="2">Tepecik-Çiftlik 37-21</td><td>Mother-son</td><td>0.19</td><td>0.41</td><td>0.23</td><td>0.77</td><td>0.00</td><td>Same</td><td>-</td><td>Adult and old adult</td><td>125,110</td><td>6385-6100
6225-6070</td></tr>
<tr><td colspan="11">We inferred this pair as likely mother-son (as opposed to brother-sister) due to their high X chromosomal θ, their high autosomal k_1 value and their C14 dates, although a father-daughter pair is also possible, but less likely. Their radiocarbon data also suggests that it is 96% probable that the woman (Individual 37) died first.</td></tr>
</table>

Table S3. Combined evidence for pedigree relationships for related pairs and its evaluation, Related to Figure 2 and STAR Methods.

Cotterman coefficients (k_0, k_1, k_2) define probabilities of sharing 0, 1 or 2 alleles identical-by-descent between a pair of individuals. The "Osteological age" and "C14 date" columns show the information for each individual in each pair in the same order as listed in the "Pair" column. Expected values for the mentioned statistics are presented in Table S2. For each pair, we provide brief discussion on how the most likely relationship was inferred.

Site	*n*	*ρ*	*P*-value	Sample criteria	Individuals excluded (justification)
Çatalhöyük	13	-0.0402	0.62	No relatives	2728 (with relatives)
Çatalhöyük	11	-0.0014	0.49	No relatives and only mid-7th mil. BCE	2728 (with relatives), 11739 and 20217 (Final Çatalhöyük layers)
Çatalhöyük	9	-0.1086	0.77	No relatives and only levels South K,M,N	2728 (with relatives), 11739 and 20217 (Final Çatalhöyük layers)
Barcın	21	-0.0857	0.76	No relatives	L11-215 and M10-271 (with relatives)
Barcın	13	0.0219	0.36	No relatives and only Layers c and d	L11-215 and M10-271 (with relatives), L11_322, M13_72, M11_59, M10_106, L11_213, L11_439, M13_170, L11_216 (from Layers a and b)
Aşıklı	6	-0.3035	0.65	No relatives	Ash131 and Ash133 (with relatives)
Aşıklı	5	0.0191	0.42	No relatives and spatial proximity	Ash131 and Ash133 (with relatives), Ash002 (spatially an outlier)
Boncuklu	7	0.0348	0.45	No relatives	ZHAF and ZHJ (with relatives)

Table S4. Correlation between genetic and spatial distances across burials, Related to STAR Methods.

The table shows the number of individuals (*n*), Pearson correlation coefficient (*ρ*) and Mantel test *P*-values (one-sided) per test. The column "Sample criteria" describes what criteria were chosen to include individuals in the analysis, where "no relatives" indicates one individual from each pair of close relatives was removed. The column "Individuals excluded" lists the IDs of individuals excluded from the analysis and the justification in parentheses.

Supplemental References:

S1. Buikstra, J.E., and Ubelaker, D.H. (1994). Standards for Data Collection from Human Skeletal Remains : Proceedings of a Seminar at the Field Museum of Natural History J. E. Buikstra, ed. (Arkansas Archeological Survey).

S2. Feldman, M., Fernández-Domínguez, E., Reynolds, L., Baird, D., Pearson, J., Hershkovitz, I., May, H., Goring-Morris, N., Benz, M., Gresky, J., et al. (2019). Late Pleistocene human genome suggests a local origin for the first farmers of central Anatolia. Nat. Commun. *10*, 1–10. 10.1038/s41467-019-09209-7.

S3. Kılınç, G.M., Omrak, A., Özer, F., Günther, T., Büyükkarakaya, A.M., Bıçakçı, E., Baird, D., Dönertaş, H.M., Ghalichi, A., Yaka, R., et al. (2016). The Demographic Development of the First Farmers in Anatolia. Curr. Biol. *26*, 2659–2666. 10.1016/j.cub.2016.07.057.

S4. Gerritsen, H. (2018). mapplots: Data Visualisation on Maps. R package version 1.5.1. https://cran.r-project.org/package=mapplots.

S5. Lazaridis, I., Patterson, N., Mittnik, A., Renaud, G., Mallick, S., Kirsanow, K., Sudmant, P.H., Schraiber, J.G., Castellano, S., Lipson, M., et al. (2014). Ancient human genomes suggest three ancestral populations for present-day Europeans. Nature *513*, 409–413. 10.1038/nature13673.

S6. Patterson, N., Moorjani, P., Luo, Y., Mallick, S., Rohland, N., Zhan, Y., Genschoreck, T., Webster, T., and Reich, D. (2012). Ancient admixture in human history. Genetics *192*, 1065–1093. 10.1534/genetics.112.145037.

S7. Auton, A., Abecasis, G.R., Altshuler, D.M., Durbin, R.M., Bentley, D.R., Chakravarti, A., Clark, A.G., Donnelly, P., Eichler, E.E., Flicek, P., et al. (2015). A global reference for human genetic variation. Nature *526*, 68–74. 10.1038/nature15393.
