# Supplementary material for: Variable kinship patterns in Neolithic Anatolia revealed by ancient genomes
Source: Curr Biol. 2021 Jun 7;31(11):2455–2468.e18. doi: 10.1016/j.cub.2021.03.050 (PMC8210650; doi:10.1016/j.cub.2021.03.050)
Supplement: Document S2. Article plus supplemental information [file mmc2.pdf]

Report

Current Biology

Variable kinship patterns in Neolithic Anatolia revealed by ancient genomes

Highlights

- Genetic kinship estimated from co-buried individuals' genomes in Neolithic Anatolia
- Close relatives are common among co-burials in Aşıklı and Boncuklu
- Many unrelated infants found buried in the same building in Çatalhöyük and Barcın
- Neolithic societies in Southwest Asia may have held diverse concepts of kinship

Authors

Reyhan Yaka, Igor Mapelli, Damla Kaptan, ..., Anders Götherström, Füsun Özer, Mehmet Somel

Correspondence

anders.gotherstrom@arklab.su.se (A.G.), fusunozer@hacettepe.edu.tr (F.Ö.), msomel@metu.edu.tr (M.S.), yakaryhn@gmail.com (R.Y.)

In brief

Yaka et al. use ancient genomes from Neolithic Anatolia and present evidence for diverse concepts of social kinship in Neolithic societies. In some communities, like Çatalhöyük, many genetically unrelated infants were buried together inside the same buildings, whereas in other sites, people buried together were frequently close biological kin.

Yaka et al., 2021, Current Biology *31*, 2455–2468
June 7, 2021 © 2021 The Authors. Published by Elsevier Inc.
https://doi.org/10.1016/j.cub.2021.03.050

CellPress

Current Biology

CellPress
OPEN ACCESS

Report

Variable kinship patterns in Neolithic Anatolia revealed by ancient genomes

Reyhan Yaka,[1,*] Igor Mapelli,[1,38] Damla Kaptan,[1,38] Ayça Doğu,[1,38] Maciej Chyleński,[2,38] Ömür Dilek Erdal,[3] Dilek Koptekin,[4] Kıvılcım Başak Vural,[1] Alex Bayliss,[5,6] Camilla Mazzucato,[7] Evrim Fer,[8] Sevim Seda Çokoğlu,[1] Vendela Kempe Lagerholm,[9,10] Maja Krzewińska,[10,11] Cansu Karamurat,[12] Hasan Can Gemici,[12] Arda Sevkar,[3] Nihan Dilşad Dağtaş,[1] Gülşah Merve Kılınç,[1,13] Donovan Adams,[14] Arielle R. Munters,[15,16] Ekin Sağlıcan,[1] Marco Milella,[17] Eline M.J. Schotsmans,[18,19] Erinç Yurtman,[1] Mehmet Çetin,[1] Sevgi Yorulmaz,[1] N. Ezgi Altınışık,[3,20] Ayshin Ghalichi,[1,21] Anna Juras,[2] C. Can Bilgin,[1] Torsten Günther,[15] Jan Storå,[9] Mattias Jakobsson,[15] Maurice de Kleijn,[22] Gökhan Mustafaoğlu,[23] Andrew Fairbairn,[24] Jessica Pearson,[25] İnci Togan,[1] Nurcan Kayacan,[26] Arkadiusz Marciniak,[27]

(Author list continued on next page)

[1]Department of Biological Sciences, Middle East Technical University (METU), Ankara, Turkey
[2]Institute of Human Biology and Evolution, Faculty of Biology, Adam Mickiewicz University, Poznań, Poland
[3]Department of Anthropology, Hacettepe University, Ankara, Turkey
[4]Department of Health Informatics, Middle East Technical University (METU)
[5]Scientific Dating, Historic England, London, UK
[6]Biological & Environmental Sciences, University of Stirling, Stirling, UK
[7]Department of Anthropology, Stanford University, Stanford, CA, 94303 USA
[8]Department of Genetics, University of Arizona, 85719, Tucson, AZ, USA
[9]Department of Archaeology and Classical Studies, Stockholm University, Stockholm, Sweden
[10]Centre for Palaeogenetics, Stockholm, Sweden
[11]Archaeological Research Laboratory, Department of Archaeology and Classical Studies, Stockholm University, Stockholm, Sweden
[12]Graduate School of Social Sciences, Middle East Technical University (METU), Ankara, Turkey
[13]Department of Bioinformatics, Graduate School of Health Sciences, Hacettepe University, 06100, Ankara, Turkey
[14]Department of Anthropology, University of Central Florida

(Affiliations continued on next page)

SUMMARY

The social organization of the first fully sedentary societies that emerged during the Neolithic period in Southwest Asia remains enigmatic,[1] mainly because material culture studies provide limited insight into this issue. However, because Neolithic Anatolian communities often buried their dead beneath domestic buildings,[2] household composition and social structure can be studied through these human remains. Here, we describe genetic relatedness among co-burials associated with domestic buildings in Neolithic Anatolia using 59 ancient genomes, including 22 new genomes from Aşıklı Höyük and Çatalhöyük. We infer pedigree relationships by simultaneously analyzing multiple types of information, including autosomal and X chromosome kinship coefficients, maternal markers, and radiocarbon dating. In two early Neolithic villages dating to the 9th and 8th millennia BCE, Aşıklı Höyük and Boncuklu, we discover that siblings and parent-offspring pairings were frequent within domestic structures, which provides the first direct indication of close genetic relationships among co-burials. In contrast, in the 7th millennium BCE sites of Çatalhöyük and Barcın, where we study subadults interred within and around houses, we find close genetic relatives to be rare. Hence, genetic relatedness may not have played a major role in the choice of burial location at these latter two sites, at least for subadults. This supports the hypothesis that in Çatalhöyük,[3–5] and possibly in some other Neolithic communities, domestic structures may have served as burial location for social units incorporating biologically unrelated individuals. Our results underscore the diversity of kin structures in Neolithic communities during this important phase of sociocultural development.

RESULTS AND DISCUSSION

Our study focuses on social organization across two Neolithic periods. The Aceramic period is represented by Aşıklı Höyük (c. 8,350–7,300 cal BCE)[6–8] and Boncuklu (c. 8,300–7,600 cal BCE)[9,10] (Figure 1A), which are among the earliest sedentary communities in Central Anatolia. During the 9th millennium these sites were characterized by small curvilinear buildings, and both

This is an open access article under the CC BY license (http://creativecommons.org/licenses/by/4.0/).

CellPress
OPEN ACCESS

Clark Spencer Larsen,[28] Ian Hodder,[7] Çiğdem Atakuman,[29,39] Marin Pilloud,[30,39] Elif Sürer,[31,39] Fokke Gerritsen,[32,39] Rana Özbal,[33,39] Douglas Baird,[25,39] Yılmaz Selim Erdal,[3,20,39] Güneş Duru,[34,39] Mihriban Özbaşaran,[35,39] Scott D. Haddow,[36,39] Christopher J. Knüsel,[19,39] Anders Götherström,[9,37,39,*] Füsun Özer,[3,20,39,*] and Mehmet Somel[1,39,40,*]

[15]Human Evolution, Department of Organismal Biology, Uppsala University, 751 05 Uppsala, Sweden
[16]SciLife Lab, Uppsala University, 751 05 Uppsala, Sweden
[17]Department of Physical Anthropology, Institute of Forensic Medicine, University of Bern, Sulgenauweg 40, CH-3007 Bern, Switzerland
[18]Centre for Archaeological Science, University of Wollongong, Wollongong, Australia
[19]UMR 5199, De la Préhistoire à l'Actuel: Culture, Environnement et Anthropologie (PACEA), Université de Bordeaux, Pessac, France
[20]Human G Lab, Department of Anthropology, Hacettepe University, Ankara, Turkey
[21]Department of Archaeogenetics, Max-Planck Institute for the Science of Human History, Kahlaische Strasse 10, D-07745, Jena, Germany
[22]Spatial Information Laboratory (SPINlab) at the Vrije Universiteit Amsterdam, Amsterdam, Netherlands
[23]Department of Archaeology, Faculty of Letters, Ankara Hacı Bayram Veli University, Abant 1 Cad. No:10/2D, Yenimahalle, Ankara
[24]School of Social Science, The University of Queensland, Michie Building, St Lucia, Brisbane, QLD, Australia
[25]Department of Archaeology, Classics and Egyptology, University of Liverpool, 8–14 Abercromby Square, Liverpool, L69 7WZ, UK
[26]Department of Prehistory, Faculty of Letters, Istanbul University, Ordu Cad. No: 6, 34459, Laleli, Istanbul
[27]Faculty of Archaeology, Adam Mickiewicz University, Poznań, Poland
[28]Department of Anthropology, Ohio State University, Columbus OH, USA 43210-1106
[29]Institute of Social Sciences, Middle East Technical University (METU), Ankara, Turkey
[30]Department of Anthropology, University of Nevada, Reno
[31]Department of Modeling and Simulation, Graduate School of Informatics, Middle East Technical University (METU), Ankara, Turkey
[32]Netherlands Institute in Turkey, Istanbul, Turkey
[33]Department of Archaeology and History of Art, Koç University, 34450 Istanbul, Turkey
[34]Mimar Sinan Fine Arts University, Istanbul 34134, Turkey
[35]Department of Prehistory, Istanbul University, Istanbul 34134, Turkey
[36]Department of Cross-Cultural and Regional Studies, University of Copenhagen, Copenhagen, Denmark
[37]Centre for Palaeogenetics, Stockholm, Sweden
[38]These authors contributed equally
[39]These authors contributed equally
[40]Lead Contact
*Correspondence: yakaryhn@gmail.com (R.Y.), anders.gotherstrom@arklab.su.se (A.G.), fusunozer@hacettepe.edu.tr (F.Ö.), msomel@metu.edu.tr (M.S.)
https://doi.org/10.1016/j.cub.2021.03.050

maintained mainly forager subsistence practices. The subsequent Ceramic Neolithic period communities were increasingly reliant on food production, and they lived in larger settlements characterized by rectilinear, clustered architecture. In our study, this later period is represented by Çatalhöyük (c. 7,100–5,950 cal BCE),[11–16] Tepecik-Çiftlik (c. 7,500–5,800 cal BCE),[17] and Barcın Höyük (c. 6,600–6,000 cal BCE).[18,19] For this study, we screened Neolithic period human remains from Aşıklı Höyük (n = 30) and Çatalhöyük (n = 60) by shotgun DNA sequencing. Owing to adverse conditions and the antiquity of the material, only n = 8 (26%) and n = 14 (23%) skeletons (all petrous bones), respectively, contained $\geq$0.1% human DNA. These were directly deep sequenced or sequenced after enrichment by whole-genome capture probes, resulting in a total of 22 genomes with 0.01× to 5.0× coverage (median = 0.09×) (Tables 1 and Z1) (tables with the prefix "Z" are supplemental data tables, which can be found at Zenodo Data: https://doi.org/10.5281/zenodo.4587657). After confirming the authenticity of the data (Figure S1A and Table Z1), we combined them with published genomes from Boncuklu Höyük (n = 9), Barcın Höyük (n = 23), and Tepecik-Çiftlik (n = 5)[20–23] (Tables S1 and Z2). We integrated this with unpublished spatial and stratigraphic data from the archaeological sites.

Increased genetic diversity from the Aceramic to the Ceramic period

We first analyzed genetic relationships at the population level. Principal components analysis (Figure 1B), *ADMIXTURE* analysis,[24] as well as F_{ST}, f_3- and *D*-statistics[25] (Figures S2A and S2B, S2D–S2F, and S3A–S3C, and Tables Z3-Z6) showed that Aşıklı Höyük and Çatalhöyük people belonged to the Central and West Anatolian early Holocene gene pool, represented by Boncuklu Höyük, Tepecik-Çiftlik, and Barcın Höyük individuals, as well as an Epipalaeolithic Central Anatolian individual from Pınarbaşı.[21] Within this regional group, we discern genetically distinct communities, such that individuals from these sites (except for Tepecik-Çiftlik) tended to share more recent common ancestry with individuals from the same settlement compared with those of other settlements (among 576–11,780 *D*-tests per site, 84%–93% were nominally significant in this direction; Figures S3D–S3F and Table Z7). F_{ST}, f_3- and *D*-statistics also showed that residents of the two Aceramic Neolithic settlements, Aşıklı Höyük and Boncuklu Höyük, were genetically highly similar to each other (Figures 1C, S2A, S2B, and S2D–S2F) relative to Ceramic Neolithic-period populations.

Aceramic Neolithic-period populations had lower within-group genetic diversity (measured using the f_3-statistic) than did Ceramic Neolithic groups (Figures 1D and S2C, and Tables Z8 and Z9) and carried a higher fraction of short runs of homozygosity (ROH) than most Ceramic Neolithic genomes (Figure S3G). This temporal increase in diversity, also noted in earlier studies,[20] could be explained by two non-exclusive scenarios, namely population growth and genetic admixture. By testing *D(Outgroup, X; Aceramic Anatolian, Ceramic Anatolian)*, where *X* represents an early Holocene Zagros or Levantine population, we found results compatible with southern and eastern gene flow into Central

CellPress
OPEN ACCESS

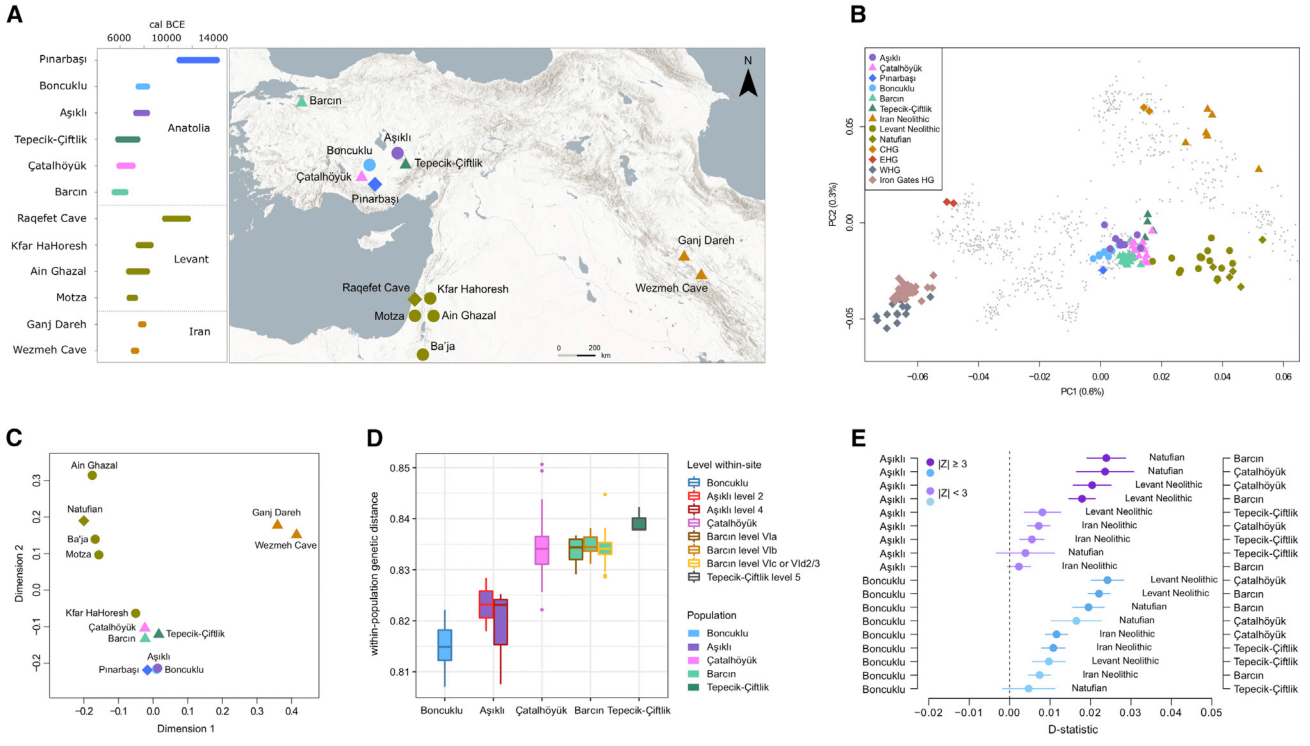

Figure 1. Population relationships in Neolithic Anatolia

(A) Geographic map of early Holocene SW Asian settlements with genome data used in the study (Table Z3). The map was created using *ArcGIS Pro*® 2.4.0 (*ArcGIS Pro*® is the intellectual property of Esri and is used herein under license. For more information about Esri® software, please visit www.esri.com. Map sources: Esri, USGS, NOAA).

(B) Principal components analysis (PCA) plot describing the genetic affinities among ancient populations studied. The genotype of each ancient individual was projected upon the first two PCs calculated using present-day West Eurasians. Colored dots represent ancient individuals. Figure S3B lists population labels of present-day individuals (gray dots).

(C) Multidimensional scaling plot summarizing f_3-statistic-based genetic distances between Anatolian populations (goodness of fit r^2 = 0.92).

(D) Boxplots showing within-population genetic distances (i.e., diversity) calculated using roughly contemporaneous individuals from each settlement (STAR Methods). Boxplot whiskers extend <1.5 times the interquartile range.

(E) Population level *D*-statistics calculated as *D(Yoruba, X; Aceramic, Ceramic)*, where *Aceramic* indicates Aşıklı and Boncuklu shown on the left-hand y axis, and *Ceramic* indicates Çatalhöyük, Barcın, or Tepecik-Çiftlik shown on the right-hand y axis, and *X* stands for ancient populations from the Levant and Iran, shown in the middle. Negative or positive *D* values indicate higher genetic affinity between *X* and Aceramic or Ceramic Neolithic Anatolians, respectively. Darker colors show nominally significant *D*-statistics with $|Z| \geq 3$, and lighter colors show non-significant values. Error bars show ± 1 standard error. See also Figures S2 and S3 and Tables Z1–Z5, Z8–Z9.

and West Anatolia between roughly 7,500 and 6,500 cal BCE (Figure 1E and Table Z4) as previously suggested.[21,26] Using *qpAdm*, we could also model Ceramic Neolithic Anatolian populations as mixtures of c.90% Aceramic Neolithic Anatolian ancestry (estimate ± 1 standard error: 89%–92% ± 2%–4%) and c.10% Levantine ancestry (8%–11% ± 2%–4%) (models that included Zagros or Caucasus populations were not supported) (Table Z10). Notably, the timing of increased population mobility is contemporaneous with a stronger reliance on agriculture and animal husbandry as food sources, a shift to larger buildings, likely population growth, and possible shifts in patterns of social organization, as we describe below.

Estimating pedigree relationships among Neolithic co-burials

Neolithic Southwest Asian settlements contain structures that are usually interpreted as domestic dwellings that served as focal points for the socialization of household members.[27] These societies frequently interred their dead, including subadults and adults of both sexes, beneath the floors of these buildings while they were inhabited by the living.[1,28,29] A common assumption has been that these burials were of household members who were related in some way, possibly genetically or through social kinship.[27,30,31] However, it is not yet clear if individuals buried under house floors necessarily lived in those structures as part of a single co-resident group[32] (STAR Methods). The extent of dietary similarity among individuals interred within the same building, for instance, is currently ambiguous.[33,34] Nevertheless, the assemblage of burials within or around domestic structures is expected to carry information about household composition and/or burial practices, and it may shed light on the relative importance of genetic relatedness as an organizing principle within these early Neolithic communities. In previous studies at Çatalhöyük, analyses of dental morphometrics and of mitochondrial DNA have suggested that individuals interred within the same building are often not genetically closely related.[3–5] The question has remained

Table 1. Archaeological, osteological, and genetic characteristics of sequenced individuals

Individual ID	Site	Stratigraphic level / area	Building	Calibrated ^{14}C date (cal. BCE)	Age class	Molecular sex	Genome coverage	Mitochondrial DNA haplogroup	Y chromosome haplogroup
2	Aşıklı Höyük	2A	AB	7585–7475 (95%)	Young adult	XX	0.02	H2a2a	-
33	Aşıklı Höyük	2C	C	7945–7890 (9%)<br>7870–7595 (86%)	Child	XY	0.07	U3a	G2a2b
40	Aşıklı Höyük	2B	BH	7935–7915 (1%)<br>7825–7590 (94%)	Old adult	XX	0.03	N1a1a1	-
128	Aşıklı Höyük	4	B3	8225–7955 (95%)	Child	XX	5.03	K1a4	-
129	Aşıklı Höyük	4	B3	8170–8115 (6%)<br>8060–8045 (1%)<br>8010–7985 (1%)<br>7970–7735 (86%)	Young adult	XX	0.79	K1a4	-
133	Aşıklı Höyük	4	B1	8170–8115 (8%)<br>8060–8040 (1%)<br>8010–7980 (2%)<br>7975–7735 (84%)	Old adult	XX	1.16	K1a4	-
131	Aşıklı Höyük	4	B1	8200–8110 (16%)<br>8095–8035 (7%)<br>8015–7740 (72%)	Child	XX	0.09	T2c1a	-
136	Aşıklı Höyük	4	B1	8175–8110 (7%)<br>8090–8075 (1%)<br>8065–8040 (1%)<br>8015–7705 (84%)<br>7695–7655 (2%)	Adult	XX	0.15	T2c1a	-
30006 F.7615	Çatalhöyük	North G	114	6645–6495 (94%)<br>6490–6480 (1%)	Infant	XX	0.07	K1a4	-
8587 F.1013	Çatalhöyük	North G	114	-	Neonate	XX	0.14	T2e	-
2728 F.258	Çatalhöyük	South M	50	6695-6505 (95%)	Infant	XX	0.08	K1a	-
2842 F.274	Çatalhöyük	South M	50	6690-6505 (95%)	Child	XX	0.09	K1a	-
2017 F.96	Çatalhöyük	South M	50	6815–6790 (2%)<br>6775–6595 (93%)	Neonate	XX	0.03	T2	-
1885 F.84	Çatalhöyük	South M	50	6905–6885 (1%)<br>6825–6635 (92%)<br>6625–6600 (2%)	Child	XY	0.07	K1a	G2a2a1
2033 F.84/86	Çatalhöyük	South M	50	6690–6590 (95%)	Child	XY	0.01	H2a2a1d	H3a1
2779 F.265	Çatalhöyük	South M	50	-	Infant	XY	0.27	H2a2a	C1a2
5357 F.576	Çatalhöyük	South K	17	7035–6680 (93%)<br>6670–6650 (2%)	Infant	XY	0.06	N1a1a1	C1a2
21855 F.8214	Çatalhöyük	South K	17	-	Child	XX	0.07	H2a2a1	-
21981 F.8153	Çatalhöyük	South N	89	-	Infant	XX	0.09	K1a17	-
5747 F.1064	Çatalhöyük	South M	91	6640–6490 (95%)	Infant	XX	0.12	T2c1	-
11739 F.1912	Çatalhöyük	TP Q-R	-	6235–6075 (95%)	Middle adult	XX	0.20	K1b1	-
20217 F.3931	Çatalhöyük	TP Q-R	-	6415–6240 (95%)	Child	XX	0.06	K1a4b	-

The "individual ID" column indicates excavation IDs, including feature number ("F.") for Çatalhöyük individuals. For details of the radiocarbon dating see Table Z2. Age codes indicate infant: 2 months–3 years; child: 3–12 years; adolescent: 12–20 years; young adult: 20–35 years; middle adult: 35–50 years; old adult: 50+ years. See also Figure S1 and Tables Z1 and Z2.

unresolved, however, due to the inability of either data type to sufficiently identify exact pedigree relationships on any one site.

Here, we re-address the question of co-burial relationships using genome data from Neolithic Anatolian communities. In order to infer reliable pedigree relationships, we used different sources of information simultaneously. First, we employed three allele frequency-based methods to infer genetic kinship coefficients: *NgsRelate*,[35] *lcMLkin*,[36] and *READ*[37] (Figures 2, S4A, and S4B). Second, to distinguish different pedigree relationships among putative first-degree pairs (e.g., siblings, mother-son, father-daughter), we used the probabilities of sharing 0, 1, or 2 alleles identical-by-descent (Cotterman coefficients; k_0, k_1, k_2), although the low coverage of our genome data constrained the utility of this latter approach (Tables S2 and Z11). Therefore, for inferring pedigree relationships we combined (a) kinship coefficients (θ) estimated from autosomal and from X chromosomal loci, (b)

CellPress
OPEN ACCESS

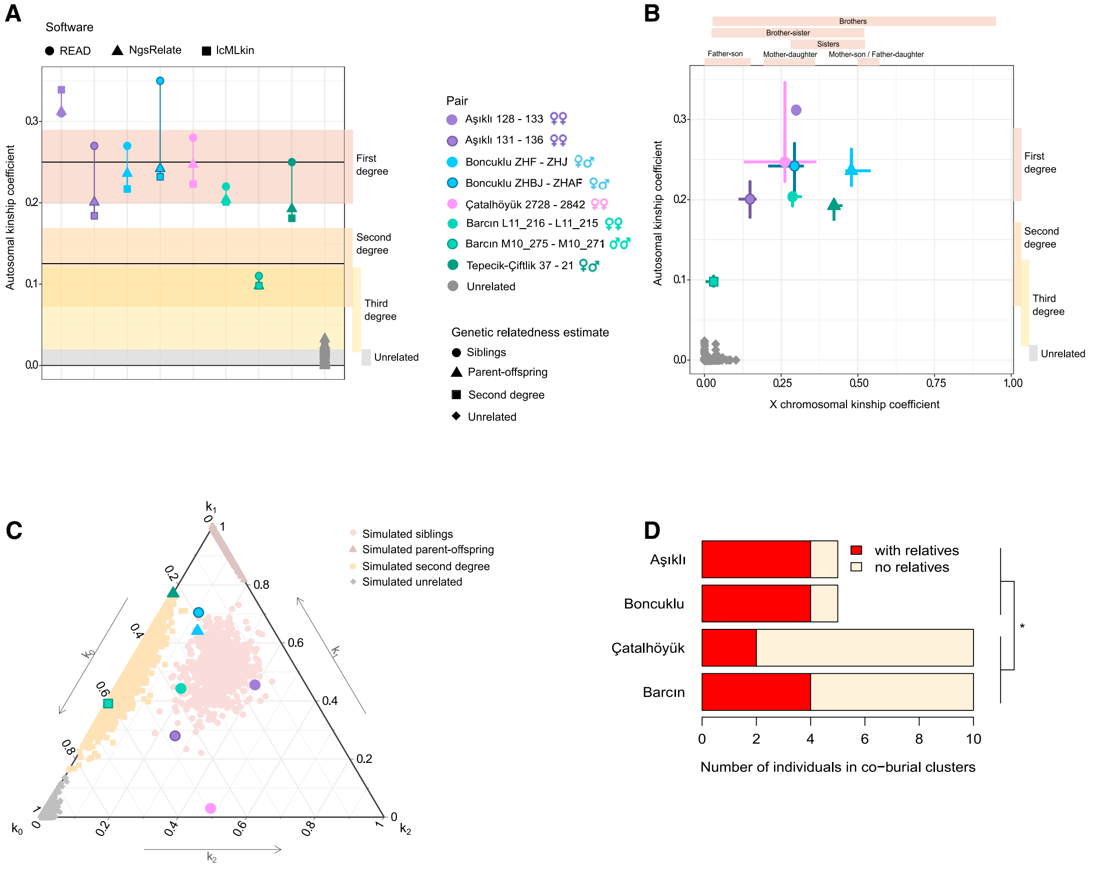

Figure 2. Genetic relatedness estimation among co-buried individuals using genomic data

(A) Autosomal kinship coefficients (θ) between pairs of individuals calculated using three different software programs. The horizontal black lines indicate expected autosomal θ values for first- and second-degree related and unrelated pairs. The high estimates for the Aşıklı 128–133 pair may be influenced by inbreeding (STAR Methods). The horizontal colored bars indicate expected θ ranges for different degrees of relatedness estimated using simulations with 5,000 SNPs (95% confidence interval). Figure S4B presents the same results, where simulations were performed using the same SNP numbers per pair.

(B) Autosomal versus X chromosomal kinship coefficients (θ) between pairs calculated with *NgsRelate*. The vertical bars on the right indicate expected θ ranges for different degrees of relatedness estimated using simulations with 5,000 autosomal SNPs, while the horizontal bars on the top indicate expected θ ranges for different types of relatedness estimated using simulations with 800 X chromosome SNPs (empirical 95% bootstrap confidence interval). The horizontal and vertical point-bars describe uncertainty in autosomal and X chromosomal θ estimates, respectively, calculated by bootstrapping SNPs 100 times (Table Z12).

(C) Probabilities of sharing 0, 1, or 2 autosomal alleles identical-by-descent (Cotterman coefficients; k_0, k_1, k_2) between pairs of individuals calculated with *NgsRelate*. The gray dots indicate expected values based on simulation. The estimated pedigree relationships reflect joint evaluation of different information (e.g., age at death) in addition to Cotterman coefficients (Table S3).

(D) Frequencies of individuals found in co-burial clusters with or without close relatives identified (Figure 3), among all co-buried individuals tested genetically in a site. (*) indicates $p < 0.05$. Including the Tepecik-Çiftlik data in the Aceramic period versus Ceramic period comparison yields an odds ratio = 6.6 and $p = 0.054$. See also Figures S1 and S4 and Tables S1–S3, Z11, Z12, Z16–Z19.

mitochondrial haplotype sharing, (c) osteological age-at-death estimates, and (d) radiocarbon dates (Table S3) (STAR Methods). Finally, we performed pedigree simulations to determine the power of kinship coefficient estimation using low coverage data (Figure S4C). In addition, we studied the performance of the kinship estimation algorithms on negative controls, that is, real data from pairs of individuals who could historically not be close relatives (STAR Methods). We hence limited the kinship tests to pairs of individuals sharing a minimum of 5,000 single nucleotide polymorphisms (SNPs) (Figure S1B). This permits reliable estimations of genetic relatedness up to the 3rd degree (e.g., cousins). Pairs related beyond the 3rd degree are here referred to as "unrelated" (STAR Methods).

The final dataset included a total of 223 pairs of individuals buried within the same sites, who were broadly contemporaneous, and who had sufficient genomic data for reliably inferring genetic relatedness (Tables S1, S3, and Z11). Of these, co-burials comprised 32 individuals and 50 pairs, including 2–6 burials associated with the same building or building clusters (i.e., co-burials). In Çatalhöyük and Barcın, co-buried individuals who could be genetically sampled only included subadults. Importantly, all these buildings either had evidence of domestic use (e.g., hearths) or lacked evidence of systematic non-domestic use (e.g., use as animal penning), and did not deviate from others of the same layer in terms of structure or elaboration.

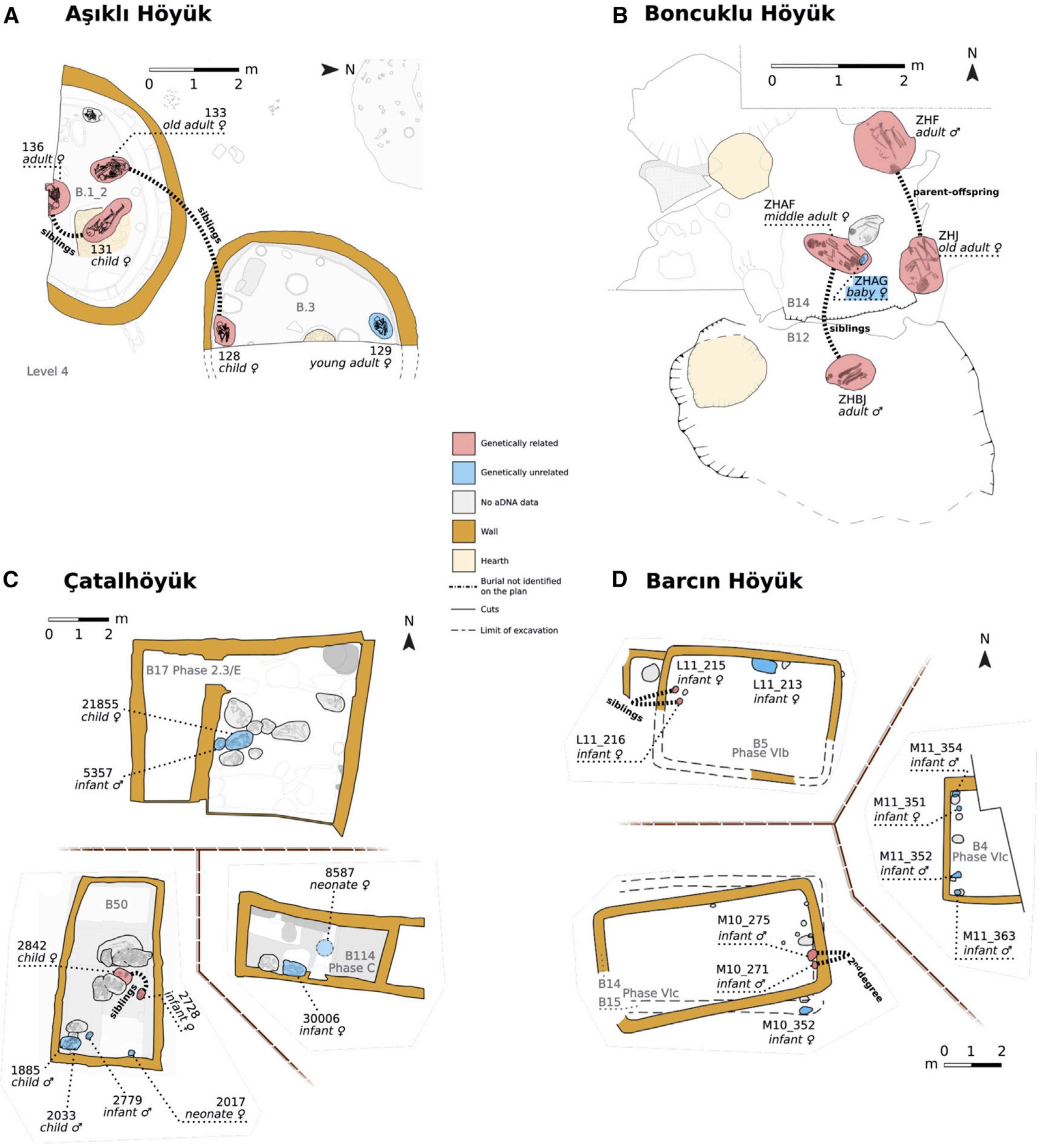

Figure 3. Relatedness among co-buried individuals in (A) Aşıklı Höyük, (B) Boncuklu Höyük, (C) Çatalhöyük, (D) Barcın Höyük

The plans show buildings where burials with identified close relatives are shown in red, burials with no identified relatives in blue, and burials for which no DNA data was available, in gray. Building numbers are shown starting with "B." The figure indicates the most likely inferred relationships, described in Table S3. See also Figure S4 and Tables S1–S3, Z11, and Z12.

Co-buried pairs in Aceramic period sites frequently include relatives

The data from Aşıklı Höyük included genomes of five individuals from the same stratigraphic layer who produced statistically consistent radiocarbon ages ($\chi^2 = 7.6$, $\chi^2(5\%) = 9.5$, n = 4; Table Z2) and could have lived at the same time. These individuals, all females, were interred in two buildings in close proximity and that shared a workspace, likely used by a single household[6] (Figure 3A). All three methods identified two pairs of first-degree relatives (Figure 2A, and Tables S3 and Z11). One pair buried in the same building included an adult and child (individuals 136 and 131). The other pair, buried in separate but proximate buildings, included an old adult and child (individual 133 and 128). The genetic and skeletal evidence indicated both pairs to be sisters (Figures 2A–2C and Tables S3 and Z11). However, we cannot exclude parent-offspring relationships. An adult female (individual 129), buried in the same building as individual 128, had no genetically close relatives among the other four individuals. Thus, although only a minority of studied individual pairs (2 of 10 pairs) were closely related, the majority of individuals studied (4 out of 5) had one close relative identified in the same or adjacent building (Figure 2D and Table S1).

The Boncuklu Höyük data comprised nine genomes of individuals who were buried in three buildings or in external spaces. Five individuals formed a co-burial cluster in two adjacent consecutive buildings (Figure 3B). Among these, two pairs of first-degree relatives were identified (Figures 2A–2C; Tables S3 and Z11) (also reported earlier[21]). The first were a possible mother and her adult son (individuals ZHF and ZHJ) and were buried in the same building (B14). Their radiocarbon results were different at the 1% significance level (χ^2 = 8.8, χ^2 (5%) = 3.8, n = 1; Table Z2), and suggested that the woman (ZHJ) died first with 90% probability. The second included a possible pair of adult male and female siblings (individuals ZHBJ and ZHAF). These individuals were buried in the proximate consecutive buildings (B12 and B14). Thus, as at Aşıklı Höyük, we could identify close relatives across the majority of individuals (4 out of 5) associated with neighboring building pairs (Figure 2D and Table S1). The only exception was a perinatal infant (individual ZHAG). Intriguingly, this infant was buried in the same grave with an adult female (individual ZHAF). The infant also shared the woman's mitochondrial haplotype but was closely related to neither the woman nor any other individual studied. Other individuals also lacked close relatives in this dataset.

Relatives are rare among Çatalhöyük and Barcın intramural burials

The Çatalhöyük data contained genomes of 14 individuals from multiple stratigraphic levels. All except one individual were subadults; 10 and 4 were genetically determined to have been females and males, respectively. Ten subadults, buried in three buildings dating to the mid-7th millennium BCE, constituted three co-burial clusters (Figure 3C). We identified a single pair of female siblings (individuals 2728 and 2842), an infant and a child, buried within the same building (Building 50) (Figures 2A–2C, and Tables S3, Z2 and Z11). The pair produced statistically consistent radiocarbon measurements (χ^2 = 0.0, χ^2 (5%) = 3.8, n = 1). None of the other pairs of individuals tested were closely related. Hence, among Çatalhöyük individuals co-buried in these three buildings and tested genetically, only 2 out of 10 had genetic kin identified (Figure 2D and Table S1).

The Barcın Höyük data included genomes of 23 individuals from multiple phases (VIa, VIb and VIc or VId2/3) (Figure 3D). Ten of these individuals were inserted into three or possibly four buildings (Table Z2). We determined two pairs of relatives, including a pair of subadult sisters (associated with Building 5) and a pair of subadult males who were second- or third-degree relatives (associated with Buildings 14/15) (Figures 2A–2C and Tables S3 and Z11). Both pairs were buried in close proximity to each other and produced statistically consistent radiocarbon measurements (L11 213 & 215, χ^2 = 0.7; M10 271 & 275, χ^2 = 0.2, χ^2 (5%) = 3.8, n = 1 for both; Table Z2). None of the other individuals had close relatives identified, including four infants buried in Building 4. Hence, among co-buried individuals we could identify relatives for only 4 out of 10 (Figure 2D and Table S1).

The Tepecik-Çiftlik data included genomes of a total of five individuals from two strata. We identified a probable pair of a mother and her adult son (individuals 37 and 21) buried in different parts of the same building (Building AY/AK) (Tables S3 and Z11). These individuals produced radiocarbon results that are different at the 1% significance level (χ^2 = 8.0, χ^2 (5%) = 3.8, n = 1), and which suggest that it is 96% probable that the woman (individual 37) died first (Table Z2).

Temporal or age-dependent variability in co-burial kinship patterns

The identification of multiple instances of close genetic relatedness among co-burials across all Neolithic Anatolian settlements studied suggests that early Neolithic social arrangements and possibly household composition were to some extent linked to genetic ties. Although long assumed,[38] genetic relatedness within Neolithic house-related social groups is documented here directly for the first time. This is particularly salient in the evidence from 9th and early 8th millennium Aşıklı Höyük and Boncuklu Höyük and could be considered suggestive of elements of close genetic kin relationships among groups buried together within Aceramic Neolithic houses.

Nevertheless, a notable fraction of our sample also contained individuals (nearly all subadults) buried in buildings together with genetically unrelated individuals (50% of 32 individuals; Figure 3). Genetic relatedness among co-burials was especially low in the 7th millennium cal BCE Çatalhöyük and Barcın Höyük, with the majority of co-burials lacking identifiable genetically related kin (the sample size from Tepecik-Çiftlik is too small to reach a general conclusion). Indeed, the combined frequencies of individuals among co-burials with and without identified relatives appeared different between Aşıklı and Boncuklu versus Çatalhöyük and Barcın Höyük (odds ratio = 8.6, Fisher's exact test p = 0.019; Figure 2D and Table S1). However, the difference becomes non-significant when including the co-buried adult pair from Tepecik-Çiftlik in the temporal comparison between Aceramic and Ceramic period sites (odds ratio = 6.6 and p = 0.054).

Two points need further mention. First, although all age groups are represented archaeologically among Çatalhöyük and Barcın Höyük burials, among samples with sufficient DNA data we had high proportions of subadults (13/14 and 16/23, respectively). This effect appears to be caused by better DNA preservation in subadult bones, at least at Çatalhöyük (Figure S1C; STAR Methods), possibly as a result of age-based differences in burial treatment.[39,40] As a consequence, in our study, no adult co-burials could be genetically examined from these two sites. Second, Çatalhöyük and Barcın Höyük buildings were significantly larger and contained more burials than those of the Aceramic Neolithic sites (Figure 3).

The infrequency of close relatives among subadults buried together in relatively large structures at Çatalhöyük and Barcın Höyük is intriguing. It raises the question of whether these buildings may have been used by extended families, such that the co-buried subadults could be distant cousins who were not identified by the methods employed. We thus tested whether individuals buried in closer proximity shared greater genetic similarity, using genetic distances based on the f_3-statistic (STAR Methods). After excluding close relatives, we found no correlation between genetic distance and spatial distance across burial pairs in either Çatalhöyük or Barcın Höyük (Pearson $r < 0.02$, Mantel test $p > 0.3$; Table S4). We also tested the hypothesis that overall genetic similarity among co-burials might be higher within buildings than between buildings. Again, we found no evidence for this (one-sided permutation test $p > 0.8$; Table S4). These results corroborate previous analyses that found no

significant correlation between burial location and dental similarities in Neolithic Çatalhöyük adults[3,4,41] and also a lack of mitochondrial DNA shared among co-burials.[5] We note that we do not expect all individuals associated with these buildings to have been buried within those structures. Also, not all individuals interred in these buildings could be sampled in this study. Still, the presence of individuals without identified relatives implies that the choice of the same structure for the burial of community members may be motivated, among other factors, by additional forms of social connectedness.[42,43] For instance, co-burials, including juveniles, may have included "adoptive, foster or fictive kin held together by memory and history making"[44]. Accordingly, co-burial and perhaps household composition in these later Neolithic settlements may have included—but also extended beyond—close genetic kin. It is also possible that the practice of co-burying subadults with genetically unrelated individuals was already present in the Aceramic period in Anatolia, but we did not sample these sufficiently in Aşıklı and Boncuklu. Indeed, the Boncuklu adult female-infant pair sharing a grave, found to be unrelated, may reflect such a tradition. It therefore remains unclear, yet, whether the difference among sites in co-burial patterns reflects a temporal shift or differential treatment of adults versus subadults in Neolithic Anatolia.

Varying traditions linking sex and space

Another set of observations involves burial patterns with respect to sex. First, we find co-burial of closely related adults of both sexes at Boncuklu Höyük and possible adult-child sister pairs at Aşıklı Höyük. Although our sample size is limited to reach a definitive conclusion, it is worth noting that the pattern is consistent with adult females retaining close ties to their natal households, symbolically or residentially, over significant periods of their lives. This scenario, at least at Boncuklu Höyük, could equally have applied to the males. Second, the sex patterning observed in Anatolian Neolithic burials appear distinct from those described for Neolithic and Bronze Age cemeteries in Europe, where male burials predominate,[45,46] and patrilocality is evident.[37,47,48] For instance, in a study of multiple cemeteries, Mittnik and colleagues identified only 2 first-degree related female pairs out of 21 first-degree relationships.[48] This proportion is different in our data, which reveals 4 first-degree related female pairs out of 7 first-degree relationships (odds ratio = 11.1, Fisher's exact test $p = 0.02$) (Table Z13). This result, as well as the contrast between co-burial of related adult females in the Aceramic period buildings and the stark patrilocal patterns in 6th-3rd millennium European cemeteries, are consistent with the notion that sex role differences intensified following the initial adoption of agriculture.[49] Meanwhile, both sister pairs we identified at Barcın and Çatalhöyük were subadults. In this regard, patrilocal traditions in Ceramic period Anatolian sites remain a possibility (as suggested earlier based on dental and mtDNA data[3,5]).

In summary, in addition to evidence for the existence of close genetic ties among putative households in the Aceramic period, we find that genetic relatedness among subadult co-burials was infrequent at Ceramic period Çatalhöyük and Barcın. Although we cannot yet pinpoint when and where this latter practice emerged, it appears plausible that during the transition from the Aceramic to the Ceramic Neolithic period in Anatolia, in parallel with changes in subsistence and population mobility, genetic relatedness may have become less important in the structuring of intramural burial traditions.

STAR★METHODS

Detailed methods are provided in the online version of this paper and include the following:

- KEY RESOURCES TABLE
- RESOURCE AVAILABILITY
  - Lead Contact
  - Materials Availability
- EXPERIMENTAL MODEL AND SUBJECT DETAILS
  - Archaeological background
  - Description of archaeological sites
  - Description of archaeological material
- METHOD DETAILS
  - Radiocarbon dating
  - Molecular biology laboratory methods
- QUANTIFICATION AND STATISTICAL ANALYSIS
  - Sequence read processing
  - Authentication of data, contamination estimates and molecular sex determination
  - Mitochondrial DNA and Y chromosome analyses
  - Dataset processing
  - Population genetic analyses
  - Estimating genetic relatedness and pedigree relationships
  - Inferring phenotypic traits and inbreeding for Aşıklı-128
  - Estimating the expected ranges for kinship coefficients using simulations
  - Spatial distances versus genetic distances among burials

SUPPLEMENTAL INFORMATION

Supplemental information can be found online at https://doi.org/10.1016/j.cub.2021.03.050.

ACKNOWLEDGMENTS

We thank all colleagues at the METU CompEvo and Hacettepe Human_G groups, and Özlen Konu for helpful discussion, the Konya Museum and the Ministry of Culture of Turkey for permissions, and three anonymous reviewers for suggestions. **Funding:** The work was supported by ERC (Consolidator Grant no. 772390 to M.S.), EMBO (Short-Term Fellowship grant no. STF 7909 to R.Y.), TÜBITAK of Turkey (grant no. 117Z229 to M.S.), AHRC/NSF (AH/M008908/1 to A.B. and I.H.), NCN of Poland (grants no. 2012/06/M/HS3/00286 to A.M., 2017/24/T/HS3/00511, and 2014/15/N/HS3/01272 to M.Ch.), National Science Foundation of the USA (Senior Biological Anthropology, NSF BCS-1827338 to M.P.), the French State via the 'Investments for the Future' framework program, Initiative d'Excellence de l'Université de Bordeaux (IdEx) (Award No. ANR-10-IDEX-03-02 to C.J.K).

AUTHOR CONTRIBUTIONS

(a) R.Y., I.M., M.Ch., İ.T., Ç.A., M.P., E.Sü., F.G., R.Ö., D.B., Y.S.E., G.D., M.Ö., S.D.H., C.J.K., A.Gö., F.Ö., and M.S. conceived and designed the study and experiments, with contributions by J.S., A.Ma., C.S.L., and I.H.; (b) Ö.D.E., N.K., A.Ma., C.S.L., I.H., Y.S.E., G.D., M.Ö., S.D.H., and C.J.K. provided the osteoarchaeological material; (c) M.Ch., Ö.D.E., M.M., E.M.J.Sc., Y.S.E., S.D.H., and C.J.K. prepared the osteoarchaeological material; (d) C.M., A.B.,

CellPress
OPEN ACCESS

C.K., H.C.G., D.A., Ç.A., M.P., F.G., R.Ö., D.B., Y.S.E., G.D., M.Ö., S.D.H., and C.J.K. compiled and analyzed archaeological data, with contributions by M.deK., G.M., A.F., J.P., and N.K.; (e) R.Y., A.D., M.Ch., D.Ka., and N.D.D. performed molecular biology laboratory experiments, with contributions and support from V.K.L., M.K., and S.Y., supervised by A.G., F.Ö., and M.S.; (f) R.Y., I.M., K.B.V., D.Ko., E.F., S.S.Ç., G.M.K., A.D., A.S., and M.S. analyzed genetic data and performed simulations, with contributions and support from M.Ch., N.D.D., M.Çe., E.Y., A.R.Mu., E.K., A.Gh., T.G., and M.J., supervised by N.E.A., E.Sü., A.G., F.Ö., and M.S.; (g) Ç.A., M.P., E.Sü., F.G., R.Ö., D.B., Y.S.E., G.D., M.Ö., S.D.H., C.J.K., A.Gö., F.Ö., and M.S. supervised the study, with contributions by C.C.B., A.J., and A.Ma.; (h) R.Y., I.M., M.M., Ç.A., M.P., E.Sü., F.G., R.Ö., D.B., Y.S.E., G.D., M.Ö., S.D.H., C.J.K., A.Gö., F.Ö., and M.S. wrote the manuscript with contributions from all authors.

DECLARATION OF INTERESTS

The authors declare no competing interests.

INCLUSION AND DIVERSITY

One or more of the authors of this paper self-identifies as a member of the LGBTQ+ community. The author list of this paper includes contributors from the location where the research was conducted who participated in the data collection, design, analysis, and/or interpretation of the work.

Received: September 19, 2020
Revised: January 8, 2021
Accepted: March 15, 2021
Published: April 14, 2021

REFERENCES

1. Kuijt, I. (2000). People and Space in Early Agricultural Villages: Exploring Daily Lives, Community Size, and Architecture in the Late Pre-Pottery Neolithic. J. Anthropol. Archaeol. *19*, 75–102. https://doi.org/10.1006/jaar.1999.0352.
2. Boz, B., and Hager, L.D. (2013). Living above the Dead: Intramural Burial practices at Çatalhöyük. In Humans and Landscapes of Çatalhöyük: Reports from the 2000-2008 Seasons, I. Hodder, ed. (Cotsen Institute of Archaeology), pp. 413–440.
3. Pilloud, M.A., and Larsen, C.S. (2011). "Official" and "practical" kin: Inferring social and community structure from dental phenotype at Neolithic Çatalhöyük, Turkey. Am. J. Phys. Anthropol. *145*, 519–530. https://doi.org/10.1002/ajpa.21520.
4. Larsen, C.S., Knüsel, C.J., Haddow, S.D., Pilloud, M.A., Milella, M., Sadvari, J.W., Pearson, J., Ruff, C.B., Garofalo, E.M., Bocaege, E., et al. (2019). Bioarchaeology of Neolithic Çatalhöyük reveals fundamental transitions in health, mobility, and lifestyle in early farmers. Proc. Natl. Acad. Sci. USA *116*, 12615–12623. https://doi.org/10.1073/pnas.1904345116.
5. Chyleński, M., Ehler, E., Somel, M., Yaka, R., Krzewińska, M., Dabert, M., Juras, A., and Marciniak, A. (2019). Ancient mitochondrial genomes reveal the absence of maternal kinship in the burials of Çatalhöyük people and their genetic affinities. Genes (Basel) *10*, 207. https://doi.org/10.3390/genes10030207.
6. Özbaşaran, M., Duru, G., and Stiner, M.C. (2018). The Early Settlement at Aşıklı Höyük: Essays in Honor of Ufuk Esin (Ege Yayınları).
7. Stiner, M.C., Buitenhuis, H., Duru, G., Kuhn, S.L., Mentzer, S.M., Munro, N.D., Pöllath, N., Quade, J., Tsartsidou, G., and Özbaşaran, M. (2014). A forager-herder trade-off, from broad-spectrum hunting to sheep management at Aşıklı Höyük, Turkey. Proc. Natl. Acad. Sci. USA *111*, 8404–8409. https://doi.org/10.1073/pnas.1322723111.
8. Abell, J.T., Quade, J., Duru, G., Mentzer, S.M., Stiner, M.C., Uzdurum, M., and Özbaşaran, M. (2019). Urine salts elucidate Early Neolithic animal management at Aşikli Höyük, Turkey. Sci. Adv. *5*, eaaw0038. https://doi.org/10.1126/sciadv.aaw0038.
9. Baird, D., Fairbairn, A., Jenkins, E., Martin, L., Middleton, C., Pearson, J., Asouti, E., Edwards, Y., Kabukcu, C., Mustafaoğlu, G., et al. (2018). Agricultural origins on the Anatolian plateau. Proc. Natl. Acad. Sci. USA *115*, E3077–E3086. https://doi.org/10.1073/pnas.1800163115.
10. Baird, D., Fairbairn, A., and Martin, L. (2017). The animate house, the institutionalization of the household in Neolithic central Anatolia. World Archaeol. *49*, 753–776. https://doi.org/10.1080/00438243.2016.1215259.
11. Bayliss, A., Brock, F., Farid, S., Hodder, I., Southon, J., and Taylor, R.E. (2015). Getting to the Bottom of It All: A Bayesian Approach to Dating the Start of Çatalhöyük. J. World Prehist. *28*, 1–26. https://doi.org/10.1007/s10963-015-9083-7.
12. Marciniak, A., Barański, M.Z., Bayliss, A., Czerniak, L., Goslar, T., Southon, J., and Taylor, R.E. (2015). Fragmenting times: Interpreting a Bayesian chronology for the Late Neolithic occupation of Çatalhöyük East, Turkey. Antiquity *89*, 154–176. https://doi.org/10.15184/aqy.2014.33.
13. Cessford, C., and Carter, T. (2005). Quantifying the consumption of obsidian at Neolithic Çatalhöyük, Turkey. J. Field Archaeol. *30*, 305–315. https://doi.org/10.1179/009346905791072279.
14. Cessford, C. (2005). Estimating the Neolithic population of Çatalhöyük. In Inhabiting Çatalhöyük: Reports from the 1995-99 Seasons, I. Hodder, ed. (McDonald Institute for Archaeological Research. British Institute for Archaeology at Ankara), pp. 323–326.
15. Hodder, I. (2000). Towards reflexive method in archaeology: the example at Çatalhöyük, I. Hodder, ed. (McDonald Institute for Archaeological Research, University of Cambridge).
16. Düring, B.S. (2010). The prehistory of Asia minor: From complex hunter-gatherers to early urban societies (Cambridge University Press). https://doi.org/10.1017/CBO9780511778926.
17. Bıçakçı, E., Godon, M., and Çakan, Y.G. (2012). Tepecik-Çiftlik. In The Neolithic in Turkey, M. Özdoğan, P.I. Kuniholm, and N. Başgelen, eds. (Archology and Art Publications), pp. 89–134.
18. Gerritsen, F., and Özbal, R. (2019). Barcın Höyük, a seventh millennium settlement in the Eastern Marmara region of Turkey. Doc. Praehist. *46*, 58–67. https://doi.org/10.4312/dp.46.4.
19. Özbal, R., and Gerritsen, F. (2019). Barcın Höyük in Interregional Perspective: An Initial Assessment. In Concluding the Neolithic: The Near East in the Second Half of the Seventh Millennium BCE, A. Marciniak, ed. (Lockwood Press), pp. 287–305. https://doi.org/10.2307/j.ctvd1c967.16.
20. Kılınç, G.M., Omrak, A., Özer, F., Günther, T., Büyükkarakaya, A.M., Bıçakçı, E., Baird, D., Dönertaş, H.M., Ghalichi, A., Yaka, R., et al. (2016). The Demographic Development of the First Farmers in Anatolia. Curr. Biol. *26*, 2659–2666. https://doi.org/10.1016/j.cub.2016.07.057.
21. Feldman, M., Fernández-Domínguez, E., Reynolds, L., Baird, D., Pearson, J., Hershkovitz, I., May, H., Goring-Morris, N., Benz, M., Gresky, J., et al. (2019). Late Pleistocene human genome suggests a local origin for the first farmers of central Anatolia. Nat. Commun. *10*, 1218. https://doi.org/10.1038/s41467-019-09209-7.
22. Hofmanová, Z., Kreutzer, S., Hellenthal, G., Sell, C., Diekmann, Y., Díez-Del-Molino, D., van Dorp, L., López, S., Kousathanas, A., Link, V., et al. (2016). Early farmers from across Europe directly descended from Neolithic Aegeans. Proc. Natl. Acad. Sci. USA *113*, 6886–6891. https://doi.org/10.1073/pnas.1523951113.
23. Mathieson, I., Lazaridis, I., Rohland, N., Mallick, S., Patterson, N., Roodenberg, S.A., Harney, E., Stewardson, K., Fernandes, D., Novak, M., et al. (2015). Genome-wide patterns of selection in 230 ancient Eurasians. Nature *528*, 499–503. https://doi.org/10.1038/nature16152.
24. Alexander, D.H., Novembre, J., and Lange, K. (2009). Fast model-based estimation of ancestry in unrelated individuals. Genome Res. *19*, 1655–1664. https://doi.org/10.1101/gr.094052.109.
25. Patterson, N., Moorjani, P., Luo, Y., Mallick, S., Rohland, N., Zhan, Y., Genschoreck, T., Webster, T., and Reich, D. (2012). Ancient admixture

in human history. Genetics *192*, 1065–1093. https://doi.org/10.1534/genetics.112.145037.

26. Kılınç, G.M., Koptekin, D., Atakuman, Ç., Sümer, A.P., Dönertaş, H.M., Yaka, R., Bilgin, C.C., Büyükkarakaya, A.M., Baird, D., Altınışık, E., et al. (2017). Archaeogenomic analysis of the first steps of Neolithization in Anatolia and the Aegean. Proc. Biol. Sci. *284*, 20172064. https://doi.org/10.1098/rspb.2017.2064.
27. Byrd, B.F. (1994). Public and Private, Domestic and Corporate: The Emergence of the Southwest Asian Village. Am. Antiq. *59*, 639–666. https://doi.org/10.2307/282338.
28. Mellaart, J. (1964). Excavations at Çatal Hüyük, 1963. Third Preliminary Report. Anatol. Stud. *14*, 39–119. https://doi.org/10.2307/3642466.
29. Mellaart, J. (1967). Çatal Hüyük: a neolithic town in Anatolia (London Thames & Hudson).
30. Flannery, K.V. (2002). The Origins of the Village Revisited: From Nuclear to Extended Households. Am. Antiq. *67*, 417–433. https://doi.org/10.2307/1593820.
31. Atakuman, Ç. (2014). Architectural Discourse and Social Transformation During the Early Neolithic of Southeast Anatolia. J. World Prehist. *27*, 1–42. https://doi.org/10.1007/s10963-014-9070-4.
32. Düring, B.S., and Marciniak, A. (2005). Households and communities in the central Anatolian Neolithic. Archaeol. Dialogues *12*, 165–187. https://doi.org/10.1017/S138020380600170X.
33. Pearson, J.A., Bogaard, A., Charles, M., Hillson, S.W., Larsen, C.S., Russell, N., and Twiss, K. (2015). Stable carbon and nitrogen isotope analysis at Neolithic Çatalhöyük: Evidence for human and animal diet and their relationship to households. J. Archaeol. Sci. *57*, 69–79. https://doi.org/10.1016/j.jas.2015.01.007.
34. Pearson, J., Lamb, A., and Evans, J. (2021). Multi-isotope Evidence of Diet (Carbon and Nitrogen) and Mobility (Strontium) at Neolithic Çatalhöyük. In Excavations at Çatalhöyük, *Volume 13*, I. Hodder, ed. (British Institute at Ankara).
35. Hanghøj, K., Moltke, I., Andersen, P.A., Manica, A., and Korneliussen, T.S. (2019). Fast and accurate relatedness estimation from high-throughput sequencing data in the presence of inbreeding. Gigascience *8*, 1–9. https://doi.org/10.1093/gigascience/giz034.
36. Lipatov, M., Sanjeev, K., Patro, R., and Veeramah, K.R. (2015). Maximum Likelihood Estimation of Biological Relatedness from Low Coverage Sequencing Data. bioRxiv, 1–20. https://doi.org/10.1101/023374.
37. Monroy Kuhn, J.M., Jakobsson, M., and Günther, T. (2018). Estimating genetic kin relationships in prehistoric populations. PLoS ONE *13*, e0195491. https://doi.org/10.1371/journal.pone.0195491.
38. Byrd, B.F. (2000). Households in transition: Neolithic social organization within Southwest Asia. In Life in Neolithic farming communities: social organization, identity, and differentiation, I. Kuijt, ed. (Kluwer Academic Publishers), pp. 63–98.
39. Haddow, S.D., and Knüsel, C.J. (2017). Skull Retrieval and Secondary Burial Practices in the Neolithic Near East: Recent Insights from Çatalhöyük, Turkey. Bioarchaeology Int. *1*, 52–71. https://doi.org/10.5744/bi.2017.1002.
40. Haddow, S.D., Schotsmans, E.M.J., Milella, M., Pilloud, M.A., Tibbetts, B., and Knüsel, C.J. (2020). From Parts to a Whole? Exploring Changes in Funerary Practices at Çatalhöyük. In Consciousness, Creativity, and Self at the Dawn of Settled Life, I. Hodder, ed. (Cambridge University Press), pp. 250–272. https://doi.org/10.1017/9781108753616.016.
41. Pilloud, M.A. (2009). Community Structure at Neolithic Çatalhöyük : Biological Distance Analysis of Household (Neighborhood, and Settlement).
42. Hodder, I. (2014). Çatalhöyük: The leopard changes its spots. A summary of recent work. Anatol. Stud. *64*, 1–22. https://doi.org/10.1017/S0066154614000027.
43. Mills, B.J. (2014). Relational Networks and Religious Sodalities at Çatalhöyük. In Religion at Work in a Neolithic Society: Vital Matters by Ian Hodder, I. Hodder, ed. (Cambridge University Press), pp. 159–186.
44. Hodder, I. (2016). More on history houses at Çatalhöyük: A response to Carleton et al. J. Archaeol. Sci. *67*, 1–6. https://doi.org/10.1016/j.jas.2015.10.010.
45. Sánchez-Quinto, F., Malmström, H., Fraser, M., Girdland-Flink, L., Svensson, E.M., Simões, L.G., George, R., Hollfelder, N., Burenhult, G., Noble, G., et al. (2019). Megalithic tombs in western and northern Neolithic Europe were linked to a kindred society. Proc. Natl. Acad. Sci. USA *116*, 9469–9474. https://doi.org/10.1073/pnas.1818037116.
46. Cassidy, L.M., Maoldúin, R.Ó., Kador, T., Lynch, A., Jones, C., Woodman, P.C., Murphy, E., Ramsey, G., Dowd, M., Noonan, A., et al. (2020). A dynastic elite in monumental Neolithic society. Nature *582*, 384–388. https://doi.org/10.1038/s41586-020-2378-6.
47. Bentley, R.A. (2013). Mobility and the diversity of Early Neolithic lives: Isotopic evidence from skeletons. J. Anthropol. Archaeol. *32*, 303–312. https://doi.org/10.1016/j.jaa.2012.01.009.
48. Mittnik, A., Massy, K., Knipper, C., Wittenborn, F., Friedrich, R., Pfrengle, S., Burri, M., Carlichi-Witjes, N., Deeg, H., Furtwängler, A., et al. (2019). Kinship-based social inequality in Bronze Age Europe. Science *366*, 731–734. https://doi.org/10.1126/science.aax6219.
49. Hansen, C.W., Jensen, P.S., and Skovsgaard, C.V. (2015). Modern gender roles and agricultural history: the Neolithic inheritance. J. Econ. Growth *20*, 365–404. https://doi.org/10.1007/s10887-015-9119-y.
50. Wilk, R.R., and Rathje, W.L. (1982). Household Archaeology. Am. Behav. Sci. *25*, 617–639.
51. Lévi-Strauss, C. (1991). Dictionnaire de l'ethnologie et de l'anthropologie, P. Bonte, and M. Izard, eds. (Presses Universitaires de France).
52. Hendon, J.A. (1996). Archaeological Approaches to the Organization of Domestic Labor: Household Practice and Domestic Relations. Annu. Rev. Anthropol. *25*, 45–61. https://doi.org/10.1146/annurev.anthro.25.1.45.
53. Bloch, M.E.F. (1998). How we think they think: anthropological approaches to cognition, memory and literacy (Oxford: Westview Press).
54. Carsten, J., and Hugh-Jones, S. (1995). About the house: Lévi-Strauss and beyond, J. Carsten, and S. Hugh-Jones, eds. (Cambridge University Press).
55. Gell, A. (1998). Art and agency: an anthropological theory (Clarendon Press).
56. Flannery, K.V. (1972). The Cultural Evolution of Civilizations. Annu. Rev. Ecol. Syst. *3*, 399–426. https://doi.org/10.1146/annurev.es.03.110172.002151.
57. Steadman, S.R. (2004). Heading home: The architecture of family and society in early sedentary communities on the Anatolian Plateau. J. Anthropol. Res. *60*, 515–558. https://doi.org/10.1086/jar.60.4.3631140.
58. Banning, E.B. (2003). Housing Neolithic farmers. Near East. Archaeol. *66*, 4–21. https://doi.org/10.2307/3210928.
59. Bar-Yosef, O. (2001). From Sedentary Foragers to Village Hierarchies: The Emergence of Social Institutions. Proc. Br. Acad. *110*, 1–38. https://doi.org/10.1186/2045-3701-4-49.
60. Goring-Morris, A.N., and Belfer-Cohen, A. (2008). A Roof Over One's Head: Developments in Near Eastern Residential Architecture Across the Epipalaeolithic-Neolithic Transition. In The Neolithic Demographic Transition and its Consequences, J.P. Bocquet-Appel, and O. Bar-Yosef, eds. (Springer), pp. 239–286. https://doi.org/10.1007/978-1-4020-8539-0_10.
61. Watkins, T. (2008). Supra-regional networks in the Neolithic of Southwest Asia. J. World Prehist. *21*, 139–171. https://doi.org/10.1007/s10963-008-9013-z.
62. Hodder, I. (2006). The Leopard's Tale: Revealing the Mysteries of Çatalhöyük (Thames &Hudson).
63. Mellaart, J. (1970). Excavations at Hacilar*Volume I-II* (The British Institute of Archaeology at Ankara).

CellPress
OPEN ACCESS

64. Roodenberg, J., Van As, A., Jacobs, L., and Wijnen, M.H. (2003). Early settlement in the plain of Yenişehir (NW Anatolia). Anatolica *29*, 17–59. https://doi.org/10.2143/ANA.29.0.2015511.

65. Marciniak, A. (2016). The Late Neolithic Transition: The Case of Çatalhöyük East. In The Origins of Food Production, N. Sanz, ed. (UNESCO), pp. 78–89.

66. Kuijt, I., Guerrero, E., Molist, M., and Anfruns, J. (2011). The changing Neolithic household: Household autonomy and social segmentation, Tell Halula, Syria. J. Anthropol. Archaeol. *30*, 502–522. https://doi.org/10.1016/j.jaa.2011.07.001.

67. Özbaşaran, M. (2012). Aşıklı. The Neolithic in Turkey (Arkeoloji Sanat Yayınları), pp. 135–158.

68. Düring, B. (2006). Constructing communities: clustered neighbourhood settlements of the Central Anatolian Neolithic CA. 8500-5500 Cal. BC. (Peeters Publishers).

69. Bienert, H.-D., Gebel, H.G.K., and Neef, R. (2004). Central Settlements in Neolithic Jordan (Ex Oriente).

70. Duru, G. (2018). Sedentism and Solitude: Exploring the impact of private space on social cohesion in the Neolithic. Religion, History, and Place in the Origin of Settled Life (University Press of Colorado). https://doi.org/10.5876/9781607327370.c006.

71. Matthews, R., Matthews, W., Richardson, A., Raheem, K.R., Walsh, S., Aziz, K.R., Bendrey, R., Whitlam, J.M.C., Bogaard, A., et al. (2019). The early Neolithic of Iraqi Kurdistan: current research at Bestansur, Shahrizor Plain. Paéorient *45*, 13–32.

72. Einwögerer, T., Friesinger, H., Händel, M., Neugebauer-Maresch, C., Simon, U., and Teschler-Nicola, M. (2006). Upper Palaeolithic infant burials. Nature *444*, 285. https://doi.org/10.1038/444285a.

73. Castex, D., and Kacki, S. (2016). Demographic Patterns Distinctive of Epidemic Cemeteries in Archaeological Samples. Paleomicrobiology of Humans (ASM Press), pp. 1–11. https://doi.org/10.1128/9781555819170.ch1.

74. Haddow, S.D., Sadvari, J.W., Knüsel, C.J., and Hadad, R. (2016). A Tale of Two Platforms: Commingled Remains and the Life-Course of Houses at Neolithic Çatalhöyük. Theoretical Approaches to Analysis and Interpretation of Commingled Human Remains (Springer International Publishing), pp. 5–29. https://doi.org/10.1007/978-3-319-22554-8_2.

75. Kuijt, I. (2002). Keeping the Peace. Life in Neolithic Farming Communities (Springer), pp. 137–164. https://doi.org/10.1007/0-306-47166-3_6.

76. Nadel, D., Danin, A., Power, R.C., Rosen, A.M., Bocquentin, F., Tsatskin, A., Rosenberg, D., Yeshurun, R., Weissbrod, L., Rebollo, N.R., et al. (2013). Earliest floral grave lining from 13,700-11,700-y-old Natufian burials at Raqefet Cave, Mt. Carmel, Israel. Proc. Natl. Acad. Sci. USA *110*, 11774–11778. https://doi.org/10.1073/pnas.1302277110.

77. Littleton, J., and Allen, H. (2007). Hunter-gatherer burials and the creation of persistent places in southeastern Australia. J. Anthropol. Archaeol. *26*, 283–298. https://doi.org/10.1016/j.jaa.2006.11.004.

78. Alt, K.W., Benz, M., Müller, W., Berner, M.E., Schultz, M., Schmidt-Schultz, T.H., Knipper, C., Gebel, H.-G.G.K., Nissen, H.J., and Vach, W. (2013). Earliest evidence for social endogamy in the 9,000-year-old-population of Basta, Jordan. PLoS ONE *8*, e65649. https://doi.org/10.1371/journal.pone.0065649.

79. Alt, K.W., Benz, M., Vach, W., Simmons, T.L., and Goring-Morris, A.N. (2015). Insights into the social structure of the PPNB Site of Kfar HaHoresh, Israel, based on dental remains. PLoS ONE *10*, e0134528. https://doi.org/10.1371/journal.pone.0134528.

80. Larsen, C.S., Hillson, S.W., Boz, B., Pilloud, M.A., Sadvari, J.W., Agarwal, S.C., Glencross, B., Beauchesne, P., Pearson, J., Ruff, C.B., et al. (2015). Bioarchaeology of Neolithic Çatalhöyük: Lives and Lifestyles of an Early Farming Society in Transition. J. World Prehist. *28*, 27–68. https://doi.org/10.1007/s10963-015-9084-6.

81. Özbaşaran, M., and Duru, G. (2015). The Early Sedentary Community of Cappadocia: Aşıklı Höyük. In La Cappadoce Méridionale de la préhistoire à la période byzantine, D. Beyer, O. Henry, and A. Tibet, eds. (3èmes Recontres d'archéologie de l'IFÉA), pp. 43–51.

82. Stiner, M.C., Bailey, K.S., Christidou, R., and Munro, N.D. (2018). Spatial and Zooarchaeological Evidence of Human-Animal Interactions in the Early PPN Settlement at Aşıklı Höyük. In The Early Settlement at Aşıklı Höyük: Essays in Honor of Ufuk Esin, M. Özbaşaran, G. Duru, and M.C. Stiner, eds. (Ege Yayınları), pp. 219–259.

83. Buitenhuis, H., Peters, J., Pöllath, N., Stiner, M.C., Munro, N.D., and Sarıtaş, Ö. (2018). The Faunal Remains from Levels 3 and 2 of Aşıklı Höyük: Evidence for Emerging Management Practices. In The Early Settlement at Aşıklı Höyük: Essays in Honor of Ufuk Esin, M. Özbaşaran, G. Duru, and M.C. Stiner, eds. (Ege Yayınları).

84. Peters, J., Neuberger, F., Wiechmann, I., Zimmermann, M., Balasse, M., and Pöllath, N. (2018). Shaping the Sheep: Human Management and Decision-making at Aşıklı Höyük, Central Anatolia. In The Early Settlement at Aşıklı Höyük: Essays in Honor of Ufuk Esin, M. Özbaşaran, G. Duru, and M.C. Stiner, eds. (Ege Yayınları), pp. 325–345.

85. Erdal, Ö.D. (2018). Lifestyle and Health Conditions of the Neolithic People of Aşıklı Höyük. In The Early Settlement at Aşıklı Höyük: Essays in Honor of Ufuk Esin., M.C. Özbaşaran, G. Duru, and M. Stiner, eds. (Ege Yayınları), pp. 405–423.

86. Baird, D. (2012). The Late Epipaleolithic, Neolithic, and Chalcolithic of the Anatolian Plateau, 13,000-4000 BC. In A Companion to the Archaeology of the Ancient Near East, D.T. Potts, ed. (Wiley), pp. 431–465. https://doi.org/10.1002/9781444360790.ch23.

87. Baird, D., Fairbairn, A., Martin, L., and Middleton, C. (2012). The Boncuklu project: the origins of sedentism, cultivation and herding in central Anatolia (Archaeology and Art Publications).

88. Byrd, B.F. (2005). Reassessing the emergence of village life in the Near East. J. Archaeol. Res. *13*, 231–290. https://doi.org/10.1007/s10814-005-3107-2.

89. Orton, D., Anvari, J., Gibson, C., Last, J., Bogaard, A., Rosenstock, E., and Biehl, P.F. (2018). A tale of two tells: dating the Çatalhöyük West Mound. Antiquity *92*, 620–639. https://doi.org/10.15184/aqy.2018.91.

90. Hodder, I., and Cessford, C. (2004). Daily Practice and Social Memory at Çatalhöyük. Am. Antiq. *69*, 17–40. https://doi.org/10.2307/4128346.

91. Bogaard, A., Fraser, R., Heaton, T.H.E., Wallace, M., Vaiglova, P., Charles, M., Jones, G., Evershed, R.P., Styring, A.K., Andersen, N.H., et al. (2013). Crop manuring and intensive land management by Europe's first farmers. Proc. Natl. Acad. Sci. USA *110*, 12589–12594. https://doi.org/10.1073/pnas.1305918110.

92. Russell, N., Twiss, K.C., Orton, D.C., and Demirergi, G.A. (2013). More on the Çatalhöyük mammal remains. In Humans and Landscapes of Çatalhöyük: Reports from the 2000-2008 Seasons, I. Hodder, ed. (British Institute at Ankara; Los Angeles: Cotsen Institute of Archaeology Press), pp. 213–258.

93. Wolfhagen, J., Veropoulidou, R., Ayala, G., Filipović, D., Kabukcu, C., Lancelotti, C., Madella, M., Pawłowska, K., Santiago-Marrero, C.G., and Wainwright, J. (2020). The seasonality of wetland and riparian task-scapes at Çatalhöyük. Near East. Archaeol. *83*, 98–109. https://doi.org/10.1086/708446.

94. Boz, B., Hager, L.D., and Haddow, S.D. (2006). Human Remains. Çatalhöyük Archive Report 2006.

95. Anvari, J., Brady, J., Franz, I., Naumov, G., Orton, D., Ostaptchouk, S., Stroud, E., Willet, P., Rosenstock, E., and Biehl, P.F. (2017). Continuous Change: Venturing into the Early Chalcolithic at Çatalhöyük in the Archaeology of Anatolia, S. Steadman, and G. McMahon, eds. (Cambridge Scholars Publishing).

96. Biehl, P.F. (2012). The transition of the megasite Çatalhöyük in the Late Neolithic and Early Chalcolithic. In Proceedings of the 7th International Congress on the Archaeology of the Ancient Near East. Volume I: Mega-Cities and Mega-Sites. The Archaeology of Consumption and Disposal, Landscape, Transport and Communication, R. Matthews, and J. Curtis, eds., pp. 17–34.

97. Knüsel, C.J., Milella, M., Betz, B., Dori, I., Garofalo, E., Glencross, B., Haddow, S.D., Ledger, M., Anastasiou, E., Mitchell, P., et al. Bioarchaeology at Neolithic Çatalhöyük: indicators of health, well-being and lifeway in their social context. In Peopling the Landscape of Çatalhöyük: Reports from the 2009-2017 Seasons. Çatalhöyük Research Project Series, Volume 13, I. Hodder, ed. (British Institute at Ankara).
98. Groenhuijzen, M.R., Kluiving, S.J., Gerritsen, F.A., and Künzel, M. (2015). Geoarchaeological research at Barcin Höyük: Implications for the initial Neolithic occupation of northwest Anatolia. Quat. Int. *367*, 51–61. https://doi.org/10.1016/j.quaint.2014.11.014.
99. Gerritsen, F.A., Özbal, R.D., and Thissen, L. (2013).). Barcın Höyük. The Beginnings of Farming in the Marmara Region. In The Neolithic in Turkey. New Excavations and New Research. Vol. 5 Northwestern Turkey and Istanbul, M. Özdoğan, N. Basgelen, and P. Kuniholm, eds. (Art and Archaeology Publications), pp. 93–112.
100. Gerritsen, F.A., Özbal, R., and Thissen, L.C. (2013). The earliest neolithic levels at Barcin Höyük, Northwestern Turkey. Anatolica *39*, 53–92. https://doi.org/10.2143/ANA.39.0.2990784.
101. Balcı, H., Cappers, R.T., Gerritsen, F.A., and Özbal, R.D. (2019). Barcın Höyük'te bitki seçimi: 2013–2015 yılı arkeo-botanik sonuçlarının degerlendirilmesi.
102. Galik, A. (2013). Barçın Höyük Zooarchaeology. https://doi.org/10.6078/M78G8HM0. http://opencontext.org/projects/74749949-4FD4-4C3E-C830-5AA75703E08E.
103. Würtenberger, D. (2012). Archäozoologische analysen am fundmaterial des Barcın Höyüks im vergleich mit aus- gewählten fundstellen des 7. und 6. Jt. v. Chr. in Nord- west- und Westanatolien.
104. Özbal, H., Thissen, L., Doğan, T., Gerritsen, F.A., Özbal, R.D., and Türkekul Bıyık, A. (2013). Neolitik Batı Anadolu ve Marmara Yerleşimleri Çanak Çömleklerinde Organik Kalıntı Analizleri. In Arkeometri Sonuçları Toplantısı, H. Dönmez, and Ö. Ötgün, eds. (Antiquity and Archeology, Art and Culture, History, Antiquity, CLUE+), pp. 105–114.
105. Evershed, R.P., Payne, S., Sherratt, A.G., Copley, M.S., Coolidge, J., Urem-Kotsu, D., Kotsakis, K., Ozdoğan, M., Ozdoğan, A.E., Nieuwenhuyse, O., et al. (2008). Earliest date for milk use in the Near East and southeastern Europe linked to cattle herding. Nature *455*, 528–531. https://doi.org/10.1038/nature07180.
106. Thissen, L., Özbal, H., Biyik, A.T., Gerritsen, F., and Özbal, R. (2010). The Land of Milk? Approaching Dietary Preferences of Late Neolithic Communities in NW Anatolia. Leiden J. Pottery Stud. *26*, 157–172.
107. de Groot, B., Thissen, L., Özbal, R., and Gerritsen, F. (2017). Clay preparation and function of the first ceramics in north-west Anatolia: A case study from Neolithic Barcın Höyük. J. Archaeol. Sci. Reports *16*, 542–552. https://doi.org/10.1016/j.jasrep.2017.06.028.
108. Lazaridis, I., Nadel, D., Rollefson, G., Merrett, D.C., Rohland, N., Mallick, S., Fernandes, D., Novak, M., Gamarra, B., Sirak, K., et al. (2016). Genomic insights into the origin of farming in the ancient Near East. Nature *536*, 419–424. https://doi.org/10.1038/nature19310.
109. Alpaslan Roodenberg, M.S., Gerritsen, F.A., and Özbal, R. (2013). Neolithic burials from Barcin Höyük: The 2007-2012 excavation seasons. Anatolica *39*, 93–111. https://doi.org/10.2143/ANA.39.0.2990785.
110. Büyükkarakaya, A.M., Çakan, Y.G., Godon, M., Erdal, Y.S., and Bıçakçı, E. (2019). Handling dead bodies: Investigating the formation process of a collective burial from Neolithic Tepecik-Çiftlik, Central Anatolia (Turkey). J. Anthropol. Archaeol. *56*, 101118. https://doi.org/10.1016/j.jaa.2019.101118.
111. Buikstra, J.E., and Ubelaker, D.H. (1994). Standards for Data Collection from Human Skeletal Remains : Proceedings of a Seminar at the Field Museum of Natural History, J.E. Buikstra, ed. (Arkansas Archeological Survey).
112. Özbaşaran, M., Duru, G., and Uzdurum, M. (2018). Architecture of the Early Settlement and Trends through the Cultural Sequence. The Early Settlement at Aşıklı Höyük (Ege Yayınları).
113. Brown, T.A., Nelson, D.E., Vogel, J.S., and Southon, J.R. (1988). Improved Collagen Extraction by Modified Longin Method. Radiocarbon *30*, 171–177. https://doi.org/10.1017/s0033822200044118.
114. Wacker, L., Němec, M., and Bourquin, J. (2010). A revolutionary graphitisation system: Fully automated, compact and simple. Nucleic Instruments Methods Phys. Res. Sect. B Beam Interact. with Mater. Atoms *268*, 931–934. https://doi.org/10.1016/j.nimb.2009.10.067.
115. Brock, F.C., Higham, T.F.G., Ditchfield, P., and Ramsey, C.B. (2010). Current pretreatment methods for AMS radiocarbon dating at the Oxford Radiocarbon Accelerator Unit (ORAU). Radiocarbon *52*, 103–112. https://doi.org/10.1017/S0033822200045069.
116. Dee, M.D., and Bronk Ramsey, C. (2000). Refinement of graphite target production at ORAU. Nucleic Instruments Methods Phys. Res. Sect. B Beam Interact. with Mater. Atoms *172*, 449–453. https://doi.org/10.1016/S0168-583X(00)00337-2.
117. Bronk Ramsey, C., Higham, T., and Leach, P. (2004). Towards High-Precision AMS: Progress and Limitations. Radiocarbon *46*, 17–24. https://doi.org/10.1017/s0033822200039308.
118. Beaumont, W., Beverly, R., Southon, J., and Taylor, R.E. (2010). Bone preparation at the KCCAMS laboratory. Nucleic Instruments Methods Phys. Res. Sect. B Beam Interact. with Mater. Atoms *268*, 906–909. https://doi.org/10.1016/j.nimb.2009.10.061.
119. Santos, G.M., Moore, R.D., Southon, J.R., Griffin, S., Hinger, E., and Zhang, D. (2007). AMS 14C sample preparation at the KCCAMS/UCI facility: Status report and performance of small samples. Radiocarbon *49*, 255–269. https://doi.org/10.1017/S0033822200042181.
120. Xu, X.M., Trumbore, S.E., Zheng, S.H., Southon, J.R., McDuffee, K.E., Luttgen, M., and Liu, J.C. (2007). Modifying a sealed tube zinc reduction method for preparation of AMS graphite targets: Reducing background and attaining high precision. Nucleic Instruments Methods Phys. Res. Sect. B Beam Interact. with Mater. Atoms *259*, 320–329. https://doi.org/10.1016/j.nimb.2007.01.175.
121. Southon, J.R., Santos, G.M., Druffel, E.R., Griffin, S., Trumbore, S., and Xu, X. (2005). High throughput, high precision 14C AMS with a small accelerator. International Atomic Energy Agency Symposium on Utilisation of Accelerators, IAEA-CN-115-10.
122. Longin, R. (1971). New method of collagen extraction for radiocarbon dating. Nature *230*, 241–242. https://doi.org/10.1038/230241a0.
123. Vogel, J.S., Southon, J.R., Nelson, D.E., and Brown, T.A. (1984). Performance of catalytically condensed carbon for use in accelerator mass spectrometry. Nucl. Instrum. Methods Phys. Res. B *5*, 289–293. https://doi.org/10.1016/0168-583X(84)90529-9.
124. Salehpour, M., Håkansson, K., Possnert, G., Wacker, L., and Synal, H.A. (2016). Performance report for the low energy compact radiocarbon accelerator mass spectrometer at Uppsala University. Nucleic Instruments Methods Phys. Res. Sect. B Beam Interact. with Mater. Atoms *371*, 360–364. https://doi.org/10.1016/j.nimb.2015.10.034.
125. Stuiver, M.J., and Polach, H.A. (1977). Reporting of 14C data. Radiocarbon *19*, 355–363.
126. Ward, G.K., and Wilson, S.R. (1978). Procedures for Comparing and Combining Radiocarbon Age Determinations: A Critique. Archaeometry *20*, 19–31. https://doi.org/10.1111/j.1475-4754.1978.tb00208.x.
127. Reimer, P.J., Austin, W.E.N., Bard, E., Bayliss, A., Blackwell, P., Bronk Ramsey, C., Butzin, M., Cheng, H., Edwards, R.L., Friedrich, M., et al. (2020). The IntCal20 Northern Hemisphere radiocarbon age calibration curve (0-55 kcal BP). Radiocarbon *62*, https://doi.org/10.1017/RDC.2020.41.
128. Stuiver, M., and Reimer, P.J. (1993). Extended 14C data base and revised CALIB 3.0 14C age calibration program. Radiocarbon *35*, 215–230. https://doi.org/10.1017/S0033822200013904.
129. Yaka, R., Birand, A., Yılmaz, Y., Caner, C., Açan, S.C., Gündüzalp, S., Parvizi, P., Erim Özdoğan, A., Togan, İ., and Somel, M. (2018). Archaeogenetics of Late Iron Age Çemialo Sırtı, Batman: Investigating maternal genetic continuity in north Mesopotamia since the Neolithic.

Am. J. Phys. Anthropol. *166*, 196–207. https://doi.org/10.1002/ajpa.23423.

130. Dabney, J., Knapp, M., Glocke, I., Gansauge, M.T., Weihmann, A., Nickel, B., Valdiosera, C., García, N., Pääbo, S., Arsuaga, J.L., and Meyer, M. (2013). Complete mitochondrial genome sequence of a Middle Pleistocene cave bear reconstructed from ultrashort DNA fragments. Proc. Natl. Acad. Sci. USA *110*, 15758–15763. https://doi.org/10.1073/pnas.1314445110.

131. Ottoni, C., Ricaut, F.X., Vanderheyden, N., Brucato, N., Waelkens, M., and Decorte, R. (2011). Mitochondrial analysis of a Byzantine population reveals the differential impact of multiple historical events in South Anatolia. Eur. J. Hum. Genet. *19*, 571–576. https://doi.org/10.1038/ejhg.2010.230.

132. Yang, D.Y., Eng, B., Waye, J.S., Dudar, J.C., and Saunders, S.R. (1998). Technical note: improved DNA extraction from ancient bones using silica-based spin columns. Am. J. Phys. Anthropol. *105*, 539–543. https://doi.org/10.1002/(SICI)1096-8644(199804)105:4<539:AID-AJPA10>3.0.CO;2-1.

133. Svensson, E.M., Anderung, C., Baubliene, J., Persson, P., Malmström, H., Smith, C., Vretemark, M., Daugnora, L., and Götherström, A. (2007). Tracing genetic change over time using nuclear SNPs in ancient and modern cattle. Anim. Genet. *38*, 378–383. https://doi.org/10.1111/j.1365-2052.2007.01620.x.

134. Meyer, M., and Kircher, M. (2010). Illumina sequencing library preparation for highly multiplexed target capture and sequencing. Cold Spring Harb. Protoc. *2010*, t5448. https://doi.org/10.1101/pdb.prot5448.

135. Günther, T., Valdiosera, C., Malmström, H., Ureña, I., Rodriguez-Varela, R., Sverrisdóttir, Ó.O., Daskalaki, E.A., Skoglund, P., Naidoo, T., Svensson, E.M., et al. (2015). Ancient genomes link early farmers from Atapuerca in Spain to modern-day Basques. Proc. Natl. Acad. Sci. USA *112*, 11917–11922. https://doi.org/10.1073/pnas.1509851112.

136. Kircher, M. (2012). Analysis of high-throughput ancient DNA sequencing data. Methods Mol. Biol. *840*, 197–228. https://doi.org/10.1007/978-1-61779-516-9_23.

137. Schubert, M., Lindgreen, S., and Orlando, L. (2016). AdapterRemoval v2: rapid adapter trimming, identification, and read merging. BMC Res. Notes *9*, 88. https://doi.org/10.1186/s13104-016-1900-2.

138. Li, H., and Durbin, R. (2009). Fast and accurate short read alignment with Burrows-Wheeler transform. Bioinformatics *25*, 1754–1760. https://doi.org/10.1093/bioinformatics/btp324.

139. Lazaridis, I., Patterson, N., Mittnik, A., Renaud, G., Mallick, S., Kirsanow, K., Sudmant, P.H., Schraiber, J.G., Castellano, S., Lipson, M., et al. (2014). Ancient human genomes suggest three ancestral populations for present-day Europeans. Nature *513*, 409–413. https://doi.org/10.1038/nature13673.

140. Skoglund, P., Northoff, B.H., Shunkov, M.V., Derevianko, A.P., Pääbo, S., Krause, J., and Jakobsson, M. (2014). Separating endogenous ancient DNA from modern day contamination in a Siberian Neandertal. Proc. Natl. Acad. Sci. USA *111*, 2229–2234. https://doi.org/10.1073/pnas.1318934111.

141. Skoglund, P., Malmström, H., Omrak, A., Raghavan, M., Valdiosera, C., Günther, T., Hall, P., Tambets, K., Parik, J., Sjögren, K.G., et al. (2014). Genomic diversity and admixture differs for Stone-Age Scandinavian foragers and farmers. Science *344*, 747–750. https://doi.org/10.1126/science.1253448.

142. Fu, Q., Mittnik, A., Johnson, P.L.F., Bos, K., Lari, M., Bollongino, R., Sun, C., Giemsch, L., Schmitz, R., Burger, J., et al. (2013). A revised timescale for human evolution based on ancient mitochondrial genomes. Curr. Biol. *23*, 553–559. https://doi.org/10.1016/j.cub.2013.02.044.

143. Li, H., Handsaker, B., Wysoker, A., Fennell, T., Ruan, J., Homer, N., Marth, G., Abecasis, G., and Durbin, R.; 1000 Genome Project Data Processing Subgroup (2009). The Sequence Alignment/Map format and SAMtools. Bioinformatics *25*, 2078–2079. https://doi.org/10.1093/bioinformatics/btp352.

144. Rasmussen, M., Guo, X., Wang, Y., Lohmueller, K.E., Rasmussen, S., Albrechtsen, A., Skotte, L., Lindgreen, S., Metspalu, M., Jombart, T., et al. (2011). An Aboriginal Australian genome reveals separate human dispersals into Asia. Science *334*, 94–98. https://doi.org/10.1126/science.1211177.

145. Skoglund, P., Malmström, H., Raghavan, M., Storå, J., Hall, P., Willerslev, E., Gilbert, M.T.P., Götherström, A., and Jakobsson, M. (2012). Origins and genetic legacy of Neolithic farmers and hunter-gatherers in Europe. Science *336*, 466–469. https://doi.org/10.1126/science.1216304.

146. Skoglund, P., Storå, J., Götherström, A., and Jakobsson, M. (2013). Accurate sex identification of ancient human remains using DNA shotgun sequencing. J. Archaeol. Sci. *40*, 4477–4482. https://doi.org/10.1016/j.jas.2013.07.004.

147. Kılınç, G.M., Kashuba, N., Yaka, R., Sümer, A.P., Yüncü, E., Shergin, D., Ivanov, G.L., Kichigin, D., Pestereva, K., Volkov, D., et al. (2018). Investigating Holocene human population history in North Asia using ancient mitogenomes. Sci. Rep. *8*, 8969. https://doi.org/10.1038/s41598-018-27325-0.

148. Weissensteiner, H., Pacher, D., Kloss-Brandstätter, A., Forer, L., Specht, G., Bandelt, H.J., Kronenberg, F., Salas, A., and Schönherr, S. (2016). HaploGrep 2: mitochondrial haplogroup classification in the era of high-throughput sequencing. Nucleic Acids Res. *44* (W1). W58-63. https://doi.org/10.1093/nar/gkw233.

149. Brandt, G., Haak, W., Adler, C.J., Roth, C., Szécsényi-Nagy, A., Karimnia, S., Möller-Rieker, S., Meller, H., Ganslmeier, R., Friederich, S., et al.; Genographic Consortium (2013). Ancient DNA reveals key stages in the formation of central European mitochondrial genetic diversity. Science *342*, 257–261. https://doi.org/10.1126/science.1241844.

150. Fernández, E., Pérez-Pérez, A., Gamba, C., Prats, E., Cuesta, P., Anfruns, J., Molist, M., Arroyo-Pardo, E., and Turbón, D. (2014). Ancient DNA analysis of 8000 B.C. near eastern farmers supports an early neolithic pioneer maritime colonization of Mainland Europe through Cyprus and the Aegean Islands. PLoS Genet. *10*, e1004401. https://doi.org/10.1371/journal.pgen.1004401.

151. Poznik, G.D., Xue, Y., Mendez, F.L., Willems, T.F., Massaia, A., Wilson Sayres, M.A., Ayub, Q., McCarthy, S.A., Narechania, A., Kashin, S., et al.; 1000 Genomes Project Consortium (2016). Punctuated bursts in human male demography inferred from 1,244 worldwide Y-chromosome sequences. Nat. Genet. *48*, 593–599. https://doi.org/10.1038/ng.3559.

152. Auton, A., Brooks, L.D., Durbin, R.M., Garrison, E.P., Kang, H.M., Korbel, J.O., Marchini, J.L., McCarthy, S., McVean, G.A., and Abecasis, G.R.; 1000 Genomes Project Consortium (2015). A global reference for human genetic variation. Nature *526*, 68–74. https://doi.org/10.1038/nature15393.

153. Mallick, S., Li, H., Lipson, M., Mathieson, I., Gymrek, M., Racimo, F., Zhao, M., Chennagiri, N., Nordenfelt, S., Tandon, A., et al. (2016). The Simons Genome Diversity Project: 300 genomes from 142 diverse populations. Nature *538*, 201–206. https://doi.org/10.1038/nature18964.

154. Günther, T., Malmström, H., Svensson, E.M., Omrak, A., Sánchez-Quinto, F., Kılınç, G.M., Krzewińska, M., Eriksson, G., Fraser, M., Edlund, H., et al. (2018). Population genomics of Mesolithic Scandinavia: Investigating early postglacial migration routes and high-latitude adaptation. PLoS Biol. *16*, e2003703. https://doi.org/10.1371/journal.pbio.2003703.

155. Patterson, N., Price, A.L., and Reich, D. (2006). Population structure and eigenanalysis. PLoS Genet. *2*, e190. https://doi.org/10.1371/journal.pgen.0020190.

156. Purcell, S., Neale, B., Todd-Brown, K., Thomas, L., Ferreira, M.A.R., Bender, D., Maller, J., Sklar, P., de Bakker, P.I.W., Daly, M.J., and Sham, P.C. (2007). PLINK: a tool set for whole-genome association and population-based linkage analyses. Am. J. Hum. Genet. *81*, 559–575. https://doi.org/10.1086/519795.

157. Behr, A.A., Liu, K.Z., Liu-Fang, G., Nakka, P., and Ramachandran, S. (2016). pong: fast analysis and visualization of latent clusters in

population genetic data. Bioinformatics *32*, 2817–2823. https://doi.org/10.1093/bioinformatics/btw327.

158. Harney, É., Patterson, N., Reich, D., and Wakeley, J. (2020). Assessing the Performance of qpAdm: A Statistical Tool for Studying Population Admixture. bioRxiv. 2020.04.09.032664. https://doi.org/10.1101/2020.04.09.032664.
159. Prüfer, K., Racimo, F., Patterson, N., Jay, F., Sankararaman, S., Sawyer, S., Heinze, A., Renaud, G., Sudmant, P.H., de Filippo, C., et al. (2014). The complete genome sequence of a Neanderthal from the Altai Mountains. Nature *505*, 43–49. https://doi.org/10.1038/nature12886.
160. Meyer, M., Kircher, M., Gansauge, M.T., Li, H., Racimo, F., Mallick, S., Schraiber, J.G., Jay, F., Prüfer, K., de Filippo, C., et al. (2012). A high-coverage genome sequence from an archaic Denisovan individual. Science *338*, 222–226. https://doi.org/10.1126/science.1224344.
161. Skoglund, P., Mallick, S., Bortolini, M.C., Chennagiri, N., Hünemeier, T., Petzl-Erler, M.L., Salzano, F.M., Patterson, N., and Reich, D. (2015). Genetic evidence for two founding populations of the Americas. Nature *525*, 104–108. https://doi.org/10.1038/nature14895.
162. Fu, Q., Posth, C., Hajdinjak, M., Petr, M., Mallick, S., Fernandes, D., Furtwängler, A., Haak, W., Meyer, M., Mittnik, A., et al. (2016). The genetic history of Ice Age Europe. Nature *534*, 200–205. https://doi.org/10.1038/nature17993.
163. Jones, E.R., Gonzalez-Fortes, G., Connell, S., Siska, V., Eriksson, A., Martiniano, R., McLaughlin, R.L., Gallego Llorente, M., Cassidy, L.M., Gamba, C., et al. (2015). Upper Palaeolithic genomes reveal deep roots of modern Eurasians. Nat. Commun. *6*, 8912. https://doi.org/10.1038/ncomms9912.
164. Mathieson, I., Alpaslan-Roodenberg, S., Posth, C., Szécsényi-Nagy, A., Rohland, N., Mallick, S., Olalde, I., Broomandkhoshbacht, N., Candilio, F., Cheronet, O., et al. (2018). The genomic history of southeastern Europe. Nature *555*, 197–203. https://doi.org/10.1038/nature25778.
165. Haak, W., Lazaridis, I., Patterson, N., Rohland, N., Mallick, S., Llamas, B., Brandt, G., Nordenfelt, S., Harney, E., Stewardson, K., et al. (2015). Massive migration from the steppe was a source for Indo-European languages in Europe. Nature *522*, 207–211. https://doi.org/10.1038/nature14317.
166. van de Loosdrecht, M., Bouzouggar, A., Humphrey, L., Posth, C., Barton, N., Aximu-Petri, A., Nickel, B., Nagel, S., Talbi, E.H., El Hajraoui, M.A., et al. (2018). Pleistocene North African genomes link Near Eastern and sub-Saharan African human populations. Science *360*, 548–552. https://doi.org/10.1126/science.aar8380.
167. Enattah, N.S., Sahi, T., Savilahti, E., Terwilliger, J.D., Peltonen, L., and Järvelä, I. (2002). Identification of a variant associated with adult-type hypolactasia. Nat. Genet. *30*, 233–237. https://doi.org/10.1038/ng826.
168. Jones, B.L., Raga, T.O., Liebert, A., Zmarz, P., Bekele, E., Danielsen, E.T., Olsen, A.K., Bradman, N., Troelsen, J.T., and Swallow, D.M. (2013). Diversity of lactase persistence alleles in Ethiopia: signature of a soft selective sweep. Am. J. Hum. Genet. *93*, 538–544. https://doi.org/10.1016/j.ajhg.2013.07.008.
169. Ranciaro, A., Campbell, M.C., Hirbo, J.B., Ko, W.Y., Froment, A., Anagnostou, P., Kotze, M.J., Ibrahim, M., Nyambo, T., Omar, S.A., and Tishkoff, S.A. (2014). Genetic origins of lactase persistence and the spread of pastoralism in Africa. Am. J. Hum. Genet. *94*, 496–510. https://doi.org/10.1016/j.ajhg.2014.02.009.
170. Allentoft, M.E., Sikora, M., Sjögren, K.G., Rasmussen, S., Rasmussen, M., Stenderup, J., Damgaard, P.B., Schroeder, H., Ahlström, T., Vinner, L., et al. (2015). Population genomics of Bronze Age Eurasia. Nature *522*, 167–172. https://doi.org/10.1038/nature14507.
171. McQuillan, R., Leutenegger, A.L., Abdel-Rahman, R., Franklin, C.S., Pericic, M., Barac-Lauc, L., Smolej-Narancic, N., Janicijevic, B., Polasek, O., Tenesa, A., et al. (2008). Runs of homozygosity in European populations. Am. J. Hum. Genet. *83*, 359–372. https://doi.org/10.1016/j.ajhg.2008.08.007.
172. Keller, M.C., Visscher, P.M., and Goddard, M.E. (2011). Quantification of inbreeding due to distant ancestors and its detection using dense single nucleotide polymorphism data. Genetics *189*, 237–249. https://doi.org/10.1534/genetics.111.130922.
173. Bhérer, C., Campbell, C.L., and Auton, A. (2017). Refined genetic maps reveal sexual dimorphism in human meiotic recombination at multiple scales. Nat. Commun. *8*, 14994. https://doi.org/10.1038/ncomms14994.
174. Kent, W.J., Sugnet, C.W., Furey, T.S., Roskin, K.M., Pringle, T.H., Zahler, A.M., and Haussler, D. (2002). The human genome browser at UCSC. Genome Res. *12*, 996–1006. https://doi.org/10.1101/gr.229102.
175. Caballero, M., Seidman, D.N., Qiao, Y., Sannerud, J., Dyer, T.D., Lehman, D.M., Curran, J.E., Duggirala, R., Blangero, J., Carmi, S., and Williams, A.L. (2019). Crossover interference and sex-specific genetic maps shape identical by descent sharing in close relatives. PLoS Genet. *15*, e1007979. https://doi.org/10.1371/journal.pgen.1007979.
176. Van Rossum, G. (1995). Python tutorial. Technical Report CS-R9526.
177. Housworth, E.A., and Stahl, F.W. (2003). Crossover interference in humans. Am. J. Hum. Genet. *73*, 188–197. https://doi.org/10.1086/376610.
178. Campbell, C.L., Furlotte, N.A., Eriksson, N., Hinds, D., and Auton, A. (2015). Escape from crossover interference increases with maternal age. Nat. Commun. *6*, 6260. https://doi.org/10.1038/ncomms7260.
179. Dray, S., and Dufour, A.B. (2007). The ade4 package: Implementing the duality diagram for ecologists. J. Stat. Softw. *22*, 1–20. https://doi.org/10.18637/jss.v022.i04.

CellPress
OPEN ACCESS

STAR★METHODS

KEY RESOURCES TABLE

REAGENT or RESOURCE	SOURCE	IDENTIFIER
**Biological Samples**		
Ash002	This study	2
Ash033	This study	33
Ash040	This study	40
Ash128	This study	128
Ash129	This study	129
Ash131	This study	131
Ash133	This study	133
Ash136	This study	136
cth006	This study	30006
cth728	This study	2728
cth842	This study	2842
cth747	This study	5747
pch034	This study	21981
CCH144	This study	5357
CCH285	This study	21855
CCH163	This study	2017
CCH289	This study	1885
CCH290	This study	2033
CCH294	This study	2779
CCH311	This study	8587
cth739	This study	11739
cth217	This study	20217
**Chemicals, Peptides, and Recombinant Proteins**		
RNase Away		N/A
Sodium Hypochloride	Sigma Aldrich	Cat#S7653
HPLC water	Sigma Aldrich	Cat#270733
Ispropanol	Merck	Cat#1009952500
Proteinase K	Thermo Fisher Scientific	Cat#E00491
Guanidine Hydrochloride	Sigma Aldrich	Cat#50950
Tween-20	BioShop	Cat#TWN508
Ethanol	Isolab	Cat#920.026.2500
EDTA disodium salt dihydrate	Sigma Aldrich	Cat#E5134
**Critical Commercial Assays**		
High Sensitivity DNA Kit (Bioanalyser 2100)	Agilent Technologies	Cat#5067-4626
MYbaits Human Whole Genome Capture Kit (African baits)	Arbor Biosciences (Ann Arbor, MI)	Cat# 302508.v5
High Sensitivity D1000 Screen Tape (Tapestation 2200)	Agilent Technologies	Cat# 5067-5584
MinElute PCR Purification Kit	QIAGEN	Cat#28004
**Deposited Data**		
Ash002 BAM file	European Nucleotide Archive (ENA)	ENA: ERS4811035
Ash033 BAM file	European Nucleotide Archive (ENA)	ENA: ERS4811084
Ash040 BAM file	European Nucleotide Archive (ENA)	ENA: ERS4811085

(*Continued on next page*)

Continued		
REAGENT or RESOURCE	SOURCE	IDENTIFIER
Ash128 BAM file	European Nucleotide Archive (ENA)	ENA: ERS4811086
Ash129 BAM file	European Nucleotide Archive (ENA)	ENA: ERS4811087
Ash131 BAM file	European Nucleotide Archive (ENA)	ENA: ERS4811088
Ash133 BAM file	European Nucleotide Archive (ENA)	ENA: ERS4811089
Ash136 BAM file	European Nucleotide Archive (ENA)	ENA: ERS4811090
cth006 BAM file	European Nucleotide Archive (ENA)	ENA: ERS4811091
cth728 BAM file	European Nucleotide Archive (ENA)	ENA: ERS4811092
cth842 BAM file	European Nucleotide Archive (ENA)	ENA: ERS4811093
cth747 BAM file	European Nucleotide Archive (ENA)	ENA: ERS4811094
pch034 BAM file	European Nucleotide Archive (ENA)	ENA: ERS4811095
CCH144 BAM file	European Nucleotide Archive (ENA)	ENA: ERS4811096
CCH285 BAM file	European Nucleotide Archive (ENA)	ENA: ERS4811098
CCH163 BAM file	European Nucleotide Archive (ENA)	ENA: ERS4811097
CCH289 BAM file	European Nucleotide Archive (ENA)	ENA: ERS4811099
CCH290 BAM file	European Nucleotide Archive (ENA)	ENA: ERS4811100
CCH294 BAM file	European Nucleotide Archive (ENA)	ENA: ERS4811101
CCH311 BAM file	European Nucleotide Archive (ENA)	ENA: ERS4811102
cth739 BAM file	European Nucleotide Archive (ENA)	ENA: ERS4811103
cth217 BAM file	European Nucleotide Archive (ENA)	ENA: ERS4811104
Oligonucleotides		
IS1_adapter.P5: 50-A*C*A*C*TCTTTCC CTACACGACGCTCTTCCG*A*T*C*T-30 (* indicates a PTO bond)	[49]	Biomers
IS2_adapter.P7: 50-G*T*G*A*CTGGAG TTCAGACGTGTGCTCTTCCG*A*T*C*T-30 (* indicates a PTO bond)	[49]	Biomers
IS3_adapter.P5+P7: 50-A*G*A*T*CGGAA* G*A*G*C-30 (* indicates a PTO bond)	[49]	Biomers
IS4: (5¢-AATGATACGGCGACCACCGAG ATCTACACTCTTTCCCTACACGACGCT CTT 3¢)	[49]	Biomers
IS5: (5¢AATGATACGGCGACCACCGA)	[49]	Biomers
IS6: (5¢ AAGCAGAAGACGGCATACGA)	[49]	Biomers
P5 indexing: (5¢-AATGATACGGCGACC ACCGAGATCTACACxxxxxxxACACTCT TTCCCTACACGACGCTCTT 3¢) where x is one of 7 different 7 bp indexes	[49]	Biomers
P7 indexing: (5'-CAAGCAGAAGACGGC ATACGAGATxxxxxxxGTGACTGGAGT TCAGACGTGT 3') where x is one of 22 different 7 bp indexes	[49]	Biomers
Software and Algorithms		
MergeReadsFastQ_cc.py	[50]	https://bioinf.eva.mpg.de/fastqProcessing/
AdapterRemoval	[51]	https://github.com/MikkelSchubert/adapterremoval
Burrows-Wheeler Aligner BWA aln 0.7.15	[52–54]	http://bio-bwa.sourceforge.net/
FilterUniqueSAMCons.py	[50]	https://bioinf.eva.mpg.de/fastqProcessing/
PMDtools	[55]	https://github.com/pontussk/PMDtools
Samtools-0.1.19	[56]	https://github.com/samtools/samtools
ANGSD	[57]	http://popgen.dk/angsd/index.php/ANGSD
HaploGrep2v2.1.1	[58]	https://haplogrep.i-med.ac.at/
EIGENSOFT	[59]	https://github.com/DReichLab/EIG

(*Continued on next page*)

CellPress
OPEN ACCESS

Continued

REAGENT or RESOURCE	SOURCE	IDENTIFIER
ADMIXTOOLS	[20]	https://github.com/DReichLab/AdmixTools
ADMIXTURE	[19]	https://dalexander.github.io/admixture/download.html
PLINK	[60]	https://www.cog-genomics.org/plink2
PONG	[61]	https://github.com/ramachandran-lab/pong
NgsRelate	[34]	https://github.com/ANGSD/NgsRelate
lcMLkin	[35]	https://github.com/COMBINE-lab/maximum-likelihood-relatedness-estimation
READ	[36]	https://bitbucket.org/tguenther/read/src/master/
HIrisPlex	[62–64]	http://hirisplex.erasmusmc.nl/
Other		
Agencourt AMPure XP beads (60 mL)	Beckman Coulter	Cat#A63881
T4 Polynucleotide Kinase (T4 PNK)	Thermo Fisher Scientific	Cat#EK0032
T4 DNA Ligase	Thermo Fisher Scientific	Cat#EL0011, EL0014
Adenine Triphosphate (ATP)	Thermo Fisher Scientific	Cat#R0441
T4 DNA Polymerase	Thermo Fisher Scientific	Cat#EP0062
dNTP Set	Thermo Fisher Scientific	Cat#R0182, R0181
dNTP Mix	Thermo Fisher Scientific	Cat#R1121, R1122
Bst polymerase, large fragment	New England Biolabs	Cat#M0275S
10X ThermoPol reaction buffer	New England Biolabs	Cat#B9004S
Amplitaq Gold 360 DNA Polymerase (with AmpliTaq Gold Buffer)	Thermo Fisher Scientific	Cat#4398833
KAPA HiFi HotStart Uracil+ Kit	Kapa Biosystems	Cat#KK2801
Herculase II Fusion DNA Polymerase	Agilent Technologies	Cat#600675
10X Tango Buffer	Thermo Fisher Scientific	Cat#BY5

RESOURCE AVAILABILITY

Lead Contact

Further information and requests for resources and reagents should be directed to and will be fulfilled by the Lead Contacts, Mehmet Somel (msomel@metu.edu.tr) and Reyhan Yaka (yakaryhn@gmail.com).

Materials Availability

This study did not generate new unique reagents.

Data and Code Availability

Ancient genome data produced for this study is deposited at the European Nucleotide Archive (ENA) under the accession number PRJEB39316 as BAM files. The computer simulation code used in the study is available at github.com/CompEvoMetu/kinshipsim. Bash scripts and R code used in regular population genetic and statistical analyses are available upon request. The supplemental data tables (identified throughout the text by the prefix Z) are available at Zenodo Data: https://doi.org/10.5281/zenodo.4587657.

EXPERIMENTAL MODEL AND SUBJECT DETAILS

Archaeological background

Neolithic buildings and households

The concept of "house" refers to a social institution through which societies define a particular type of membership group, i.e., the "household." What defines a household is based upon the cooperating individuals' criteria for relatedness, task-orientation, and co-residence.[50] These criteria are socio-culturally constructed and therefore highly variable across societies. For example, household members can be genetically related, as in genetic kin-based family organizations, but a household can also be composed of individuals who co-reside and share tasks with reference to relatedness criteria other than genetic ties. Nevertheless, these criteria of relatedness, genetic or otherwise, are considered legitimate only if they express continuity through successful invention and manipulation of concepts such as descent, belonging and other social differences based on age, sex and skill, all of which are also actively employed in terminologies of kinship or affinity.[51] Within this context, long-lasting architecture has been the most potent embodiment of inclusion and relatedness, through which a household membership and its history can be represented via a variety of symbolic activities.[52–55]

Some of the earliest long-lasting residential architecture, considered to be the primary context for the socialization of household members, is found in early Neolithic SW Asia, c. 10th-7th millennia cal BCE. The criteria that define relatedness among the household members of these societies, however, have long been debated: were the co-residents genetic kin, or did other factors determine household membership? Based on the size and form of the buildings, it has been suggested that the earlier curvilinear structures of the c.10th-9th millennium cal BCE were used by extended families, perhaps related to polygamous social structures, whereas the adoption of larger rectilinear and compartmentalized buildings of 8th-7th millennium cal BCE reflects a shift to close genetic kin-based organization.[56,57] Alternatively, given the relatively small size of most Neolithic residential structures, regardless of shape, it has been postulated that these buildings were mostly used by nuclear families.[38] Other researchers hold that the transition from some form of nuclear family household to increasingly autonomous family households occurred during the Pre-Pottery Neolithic B (PPNB).[38,58–62] Yet others argue that the increasingly autonomous households only occurred in the Late Neolithic as an element of multiscalar transformations of Neolithic communities in this period.[63–65]

Research on mortuary practices also underlines the broad regional changes through time, including suggested shifts from community membership to increasingly separate and autonomous household organizations in the PPNB.[1,66] Meanwhile, the repeated construction of mudbrick buildings at the same location over multiple generations, sometimes even maintaining the position of internal structures such as hearths, implies the presence of distinct household identities in these societies.[10,62,67]

Do co-burials represent households?

One potential source of information that could help resolve the nature of Neolithic household composition and social organization comes from burials within buildings during their occupancy. Neolithic SW Asian societies frequently practiced the burial of individuals beneath the floors of domestic buildings, usually during the time these structures were inhabited.[28,29] A common assumption has been that these burials were of household members and were related in some way, possibly genetically or through kinship based on other factors.[27,30,31] This could include households composed of families of closely genetically related individuals, extended families, multi-family households, or social units where genetic relatedness had little role. In reality, however, it remains unclear whether individuals buried under house floors lived in the same building as part of a co-resident group, i.e., whether they represented households.[32,68]

If co-burials were indeed household members, we may expect them to share specific attributes more with each other than with other co-burial groups; most notably, elements of their diet. Evidence on dietary similarity among Neolithic Anatolian households is currently equivocal. A 2015 study reported no dietary differentiation among Çatalhöyük co-burials in different buildings.[33] A 2020 study using a wider dataset again from Çatalhöyük reported statistically significant differentiation in carbon and nitrogen isotope values among buildings.[34] This same work further reported significant dietary differences among neonates buried in different buildings. Still, possible confounding factors that could influence stable isotope profiles (age and sex for adults, pathological conditions for neonates) were not explicitly controlled for in these analyses and we therefore consider these results as preliminary.

There exist additional arguments against the hypothesis that co-burials represent households. It appears that the average number of burials per residential structure is generally too small to represent full households.[69] For instance, in Aşıklı Höyük, only 90 burials have been discovered from more than 400 rooms excavated.[70] This suggests additional factors influenced the choice of burial locations and type of funerary treatments of individuals. Furthermore, an apparent excess of burials in some residential buildings, in sites such as Çatalhöyük,[62] and occasionally at other sites such as Abu Hureyra and Bestansur (although the relevant buildings here may not be ordinary residential structures),[71] implies a special role of some residential buildings for burial of individuals who probably had originally lived in other residences. Düring and Marciniak's (2005) analysis[32] of Çatalhöyük houses also indicates that human burials in buildings may have served to advertise the temporal continuity (history) of the buildings, which thus ensured the continuity and success of the household, regardless of their genetic ties.

If co-burials do not represent household members, their interment in the same buildings could be driven by at least two distinct traditions. First, individuals may be buried together because they died at the same time (e.g.,[72]). This could also include mass burials following disease outbreaks.[73] However, in the case of co-burials in Neolithic Anatolian settlements, the mortuary context and mortality profiles do not indicate mass burials. The evidence overall suggests these were collective burials, where individuals were buried sequentially, as is prevalent at Neolithic Çatalhöyük as well as other sites.[74]

Second, co-burial patterns may reflect local traditions stipulating specific burial arrangements of individuals who do not belong to the same household. Such traditions could involve burying individuals of specific status or social backgrounds together. The motivation behind these traditions may be to maintain social and economic ties among groups and to "consolidate community membership"[75]. For instance, it has been suggested that the emergence of cemeteries during the Natufian period could have represented "the establishment or strengthening of special interest groups, inheritance of corporate property, and territorial ownerships"[76]. Another example could be traditions such as described for Aboriginal Australian groups where the corpses of deceased young children were retained by the mothers to be interred with an adult male who dies next (Musgrave 1930, cited in[77]). If such arrangements were in place also in Neolithic Anatolian settlements, we might expect no direct social or genetic connection among co-burials.

Relatedness among co-burials

Studies on genetic relatedness among co-burials in Neolithic SW Asia have yet been limited. Most work to date relies on dental metric and non-metric traits as proxies for genetics,[3,78–80] and one recent study used mitochondrial DNA.[5] These studies have reported patterns consistent with endogamy[34,78] or with matrilocality in Neolithic Levantine sites,[79] and with patrilocality at Çatalhöyük.[3,80] Meanwhile, the Çatalhöyük studies, based either on dental analysis or mitochondrial DNA, found no evidence for individuals buried in the same building being more closely related to each other than to individuals buried in other buildings.[3,5,80] Still, owing to the

inability to estimate the degree of kinship using dental traits and mitochondrial data, the question of kinship among co-burials in Neolithic SW Asia has remained largely unresolved. Ancient genomics, in turn, can be used as a powerful tool to determine genetic relatedness and kinship among households of the dead, allowing further consideration of how burial locations might have structured relationships between households of the living and the construction of kinship, as well as social memory and social traditions in general. With some temporal depth to our study we are also able to consider if there might be temporal trends in these social practices over the long term.

Description of archaeological sites

Description of Aşıklı Höyük

Aşıklı Höyük, located in the Volcanic Cappadocia Region in eastern Central Anatolia is one of the earliest sedentary communities of the region, radiocarbon dated to the mid-9th and 8th millennium BCE (8350-7300 cal BCE). Excavations at the site started in 1989 as salvage excavations under the direction of Prof. Ufuk Esin (İstanbul University). Since 2010, the research and excavation project has been led by Prof. Mihriban Özbaşaran (İstanbul University) and Güneş Duru (Mimar Sinan Fine Arts University) in collaboration with an international team from various universities and institutions.

The first inhabitants of Aşıklı settled near the western bank of the Melendiz River. The river, flowing from the Ihlara Valley, and the volcanic landscape provided a rich habitat for various animal and plant species. A warm climate and park-woodland vegetation was dominant in the region during the beginning of the Holocene.[6,67] The mid-9th millennium BCE inhabitants of the site lived in semi-subterranean, oval mudbrick buildings that were reconstructed and renewed periodically at the same location. Characteristics of these buildings include hearths, a small platform, grinding stones and burial pits. Daily life was organized outside the buildings, in open activity areas, where many of the daily tasks were conducted.[81]

Archaeozoological data attest to broad spectrum hunting during the 9th millennium BCE, including a variety of small prey animals, birds and fish, although the main focus was always on sheep/goat.[7,82] Analyses of micromorphology and soil chemistry, and the presence of primary dung layers attest to the fact that animals were kept on-site, inside wattle and daub enclosures. Archaeozoological data, as well as isotope analysis show that caprines, specifically sheep, were kept in the settlement from the earliest levels; management and the domestication process continued all through the sequence.[7,82–84] The community had the knowledge and the experience of growing plants and cultivating wild and domestic cereals. Wild plants, legumes and fruits were among the gathered plants.

With the start of the 8th millennium BCE, changes took place in architecture and settlement patterns. Rectangular structures replaced the oval and semi-subterranean buildings. These rectangular buildings were mostly single-roomed. Although few in number, buildings with two or three-room buildings are also present. Toward the end of the settlement occupation, buildings started to cluster. Building clusters, generating neighborhoods, were separated by narrow spaces or passages with access to communal middens.[70] Separated by a "gravel street" from the residential area, to the southwest of the present mound, lies a building complex distinguished from domestic buildings in terms of its plan, size, construction material, internal architectural features and floor and wall treatment. The architectural features and the characteristics of the archaeological material (i.e., the dominance of wild cattle) permit interpretation of this area as a "public area" where communal consumption and certain ceremonies took place. Evidence of communal activities in this area indicates the continuity of the collective way of living,[70] while the daily activities in the residential area most probably took place on the flat roofs and inside the dwellings. During this period, hunting and gathering continued, though with less importance, and subsistence was based mainly on sheep and goat, but these animals were no longer kept within the settlement.[6]

Two concepts, a communal way of life and continuity, characterized the social organization of the Aşıklı community. Interaction with other regions and communities had a certain tempo during the mid-9th millennium BC, as evidenced by the material culture. However, simultaneous with the increasing focus on the full establishment of sedentism and caprine management, the pace of interaction decreased, only to increase again during the last 200-300 years of site occupation, corresponding roughly to the second half of the 8th millennium BC. This is well illustrated by the sudden appearance of non-local materials and technologies during this period. In contrast with this pattern of temporal change, continuity of certain elements, such as the location of the buildings and interior architectural features, constant renewal and maintenance of the floors and walls of buildings, and the transferring of objects and know-how was another factor that characterized the social fabric of the community. The inhabitants managed to live in cohesion throughout the occupation sequence and the communal way of life was maintained with new solutions, but also continuity through temporal changes and transformations was the main characteristic of the newly established Neolithic way of life at Aşıklı Höyük.

The burial customs consist of intramural, single sub-floor inhumations. The deceased were buried in pits under the floors of the buildings in a flexed position. To date, 90 burials have been found in 400 rooms. Although this tradition was not subject to change for hundreds of years, new practices arose during the latest levels of the occupation at the site. The dead were not buried with any items of personal adornment during the mid-9th millennium BCE. However, changes can be observed toward the mid-8th millennium when some individuals are found buried with ornaments. Of the 82 individuals subjected to bioarchaeological analysis, adults constitute 60% while children make up 40%. Of the 46 adults for whom sex can be determined, females constitute 65% while males constitute 35%, a marginally significant difference (binomial test $p = 0.054$). In terms of the daily activities conducted by the Aşıklı Höyük individuals, task-related pathologies of adults show that the shoulders, hips, ankles, elbows and knees were affected by osteoarthritis, possibly stemming from habitual stress. Males exhibit significant degrees of shoulder osteoarthritis, followed by their elbows and hips; for females the ankles were most affected by this disease, followed by the shoulders and hips. This may suggest that males were routinely engaged in activities such as carrying heavy loads, throwing, walking and kneeling, and females were probably engaged in activities that involved walking and squatting.[85]

Five of these burials genetically studied here were interred in Building 1 and Building 3 of Aşıklı Höyük Layer 4 (Figure 3A). These are buildings in direct proximity with less than 1 m between them, which showed temporal overlap in their periods of use, and which shared a common open workspace between them.[6] We therefore treated the individuals from both buildings as a cluster of co-burials, who might represent members of the same household.

Description of Boncuklu Höyük

Boncuklu is situated in the middle of the SW Konya basin (37°45'N 32°52'E) and lies 33.4 km northwest of the site of Pınarbaşı and 9.5 km northeast from Çatalhöyük. The site was discovered during the archaeological survey under the direction of Prof. Douglas Baird from the University of Liverpool, UK. Excavations directed by Baird began in 2006 and continue at the present time.[9] Baird was joined by co-directors, Prof. Andrew Fairbairn of University of Queensland, Australia and Dr. Gökhan Mustafaoğlu of Ankara Haci Bayram Veli University, Turkey, since 2011. Occupation of the site is documented from 8300-7600 cal BCE directly through radiocarbon dating. However, stratigraphic and material evidence suggest a slightly longer span of occupation.[9,86,87]

The exploitation of wild resources seems to have predominated, especially wild cattle and boar, fish and wetland birds, along with nuts and fruits from surrounding hill areas.[9,87] Small-scale cultivation of wheat, lentils and peas was an additional modest component of subsistence activities.[9] The chipped stone industry was microlithic, in significant contrast to broadly contemporary Levantine PPNB and northern Fertile Crescent assemblages and thus shows significant continuities with the earlier, local Epipalaeolithic and the earlier 10th/early 9th millennium BC community at Pınarbaşı in technology and raw material.[9,86] Continuities between Epipalaeolithic and early Holocene forager communities and the community at Boncuklu are clear. This evidence is supported by significant genetic continuity.[21] By 8300 cal BCE it appears local foragers adopted domestic plants from areas to the south and east, incorporating them into their traditional wetland exploitation practices.[9] They were probably introduced to the region as a consequence of the far-reaching and continuous interactions with neighboring regions from the Epipalaeolithic through the 10th-early 9th millennia cal BCE, as also documented at earlier and contemporary Pınarbaşı.[9]

The site possessed a number of sunken-floored sub-oval domestic buildings with mudbrick walls. The households display highly structured use of internal house space, divided into a 'clean' area presumably for sleeping, socializing and food consumption and a 'dirty' kitchen area.[10,87] The houses were very regularly refurbished, plastered and modified, especially the hearth areas, showing the intensity of domestic use. The floor area of these houses is small and modeling shows small intimate household units with intensive and repetitive domestic practices.[10] Evidence of ritual and symbolism in the 'clean' areas, including burials, is regular[10] and differentiated from house to house suggesting creation and maintenance of distinctive household identities.[10] The Boncuklu houses were also repeatedly and continuously reconstructed over multiple generations in the same location, a practice at some other 10th-7th millennia cal BCE sites in the surrounding regions, for example to the northeast at Aşıklı from 8300 cal BCE,[67] just to the south at Çatalhöyük from 7100 cal BCE,[62] in the Levant at PPNA Jericho[88] and in PPNB Tell Halula.[66] This seems to be a symbolic statement of household continuity. This expression of continuity and identity suggests small tight-knit households in continuous occupation of these domestic structures, whatever the nature and dynamism of their composition, which we can start to grasp through aDNA evidence. Nevertheless, there seems evidence that some broader corporate social practices cross-cut households, including some practices involved in food and resource exploitation in the wider landscape.[10]

Primary inhumations were placed under the 'clean' area of the houses during their occupation. It seems the dead 'ancestors', whether biologically related or not, were kept close to the living. In the case of Boncuklu the modest numbers of burials under house floors, maximum 5 and more usually 1-3, per house, suggest many of these could easily be members of the household that lived in these buildings, although we certainly cannot assume that to be so. Nevertheless, reflecting the fact they occurred within the houses while still in use and that these were small-sized buildings with very intimate spaces, presumably means the co-burial of the dead expressed some type of relationship to the households of the living, and thus represented a symbolic statement of connection between the dead and the living. Indeed, evidence attests to ongoing attention to burials and knowledge of their location.[10]

There were also primary burials and burials of deliberately disarticulated human remains, including human crania, in open areas between buildings in areas of midden accumulation.[10] More than 37 Neolithic burials, plus a minimum of 274 individual bones and 129 isolated finds of human remains have been studied, although more have been excavated. Nine skeletal samples from securely stratified 9th-8th millennia cal BCE burials in Areas H, K, and M provided sufficient aDNA preservation for genetic analysis (Table Z2), and thus genomic data.[20,21] Boncuklu human remains do not reflect significant disproportionate representation of males or females and there is an even spread across most age categories, including children and young, middle and old adults, with a slight, but not unusual, lesser presence of older children/adolescents.

Five of these burials (ZHF – Grave 14, ZHJ – Grave 15, ZHAF – Grave 18, ZHAG – Grave 18 and ZHBJ – Grave 30), including 2 pairs of individuals with first-degree genetic relationships were all articulated primary inhumations stratified within a sequence of 2 buildings in Area H, Building 12 and Building 14. Building 12 predates Building 14 and, indeed, the foundation cut for Building 14 removed the northern edge of Building 12 (Figure 3B). Building 14 seemed a direct replacement for Building 12, an example of the continuous reconstruction of the buildings in the same locations, although in this case with some shift of the house to the north. ZHBJ, the likely brother of ZHAF (Table S3), was buried in the northern part of Building 12 (Figure 3B). ZHAF, his likely sister, was buried in the southern part of Building 14 (Figure 3B). This seems a deliberate attempt to keep these individuals close at death and points to the close connections between the living and dead in these households. Both these burials had similar orientations, approximately west-east/northwest-southeast with heads at the West. It is thus tempting to think this might also reflect their close relationship. It may well have done, but these are the most common burial orientations at Boncuklu, among c. 70% of the analyzed burials and so might simply reflect these broader patterns. ZHAG, a female perinatal child that likely died at birth, was placed directly against the pelvis of

CellPress
OPEN ACCESS

ZHAF, but was genetically unrelated to that adult female ZHAF and also unrelated to ZHF and ZHJ in the same building. It is, of course, possible her mother lived in Building 14 but was genetically unrelated to the other adults buried there, or that as a result of some form of connection to the child and/or her mother she was buried with ZHAF, albeit from a household who lived elsewhere. ZHF and ZHJ, most likely adult son and mother respectively (Table S3), were located in the more eastern parts of Building 14 (Figure 3B). The orientation of their bodies was not dissimilar, ZHF had the common northwest-southeast and ZHJ a north-south orientation. However, their heads were at opposite ends of the grave-cut, ZHF to the northwest and ZHJ to the south. It is, therefore, difficult to suggest that orientation at Boncuklu was a direct expression of close family relationships.

ZHAJ was a primary inhumation burial of an adult male that predated Building 14 and seems to have been located in an open area. ZHB was the burial of an adult female burial post-dating Building 14. Overlying stratigraphy was eroded so it was unclear whether the grave for ZHB was cut through the floor of a building or was placed in an external area. These two burials do not show any close genetic relationships to the other sampled individuals.

The other burials analyzed, genetically unrelated to any of these burials in Area H, was one adult male primary inhumation, ZKO, buried in Building 9 in Area K, broadly contemporary but c. 15 m from the cluster in Area H. ZMOJ was a primary inhumation in an external area in the middens of Area M, located c. 25 m from the cluster in Area H. Although well stratified in Neolithic deposits the chronological relationship with the Area H cluster and ZMOJ is not clear.

Description of Çatalhöyük

Located 9 km to the south of Boncuklu Höyük on the Konya Plain in Central Anatolia, the site of Çatalhöyük was discovered and first excavated between 1961-1965 by James Mellaart (British Institute of Archaeology at Ankara), and later between 1995-2015 by Ian Hodder (Stanford University). Çatalhöyük was designated a UNESCO World Heritage Site in 2012.

The site consists of two separate mounds or "tells." The larger East Mound covers an area of 13 ha and has been dated to c. 7100-5950 cal BCE,[11] corresponding roughly to the Ceramic Neolithic period. The smaller West Mound dates to the Early Chalcolithic and was occupied until the middle of the 6th millennium BCE.[89] The Neolithic East Mound, until c. 6300 cal BCE, is characterized by dense clusters of mudbrick domestic structures interspersed with external spaces used for refuse disposal, animal penning and other daily activities. To date, large-scale, clearly identifiable public structures have not been documented at the site. Instead, individual houses at Çatalhöyük appear to have served as the focal point not only for domestic activities such as craft production, food storage and processing, but also ritual behaviors such as burials, wall paintings and other architectural embellishments associated with an elaborate symbolic repertoire and reflecting a complex socio-cultural environment.[90] There is ample evidence for the cultivation of domesticated cereal crops and the keeping of domesticated sheep and goats at the site.[91,92] Wild animal species, including aurochs, also formed part of the diet, and in the later occupation phases (6500-5950 cal BC) there is evidence for the herding of domesticated cattle.[92,93]

Between 1993 and 2017 the skeletal remains of over 700 individuals had been recovered from stratified Neolithic contexts at Çatalhöyük.[4] Primary inhumations (n = 471 individuals) placed beneath the floors of houses are the dominant burial type at the site.[2,40] Individuals were typically buried in narrow oval pits under the eastern and northern platforms of the central room, although prenates, neonates and infants were also recovered from within side rooms and near ovens and hearths.[2,40,94] Secondary burials of loose or partially articulated skeletal remains, often in association with primary burials, are also observed, although less frequently.[39,40] Intramural burials became increasingly rare toward the end of the occupation of Çatalhöyük East,[12,40] while burials are almost completely absent within the settlement on the Chalcolithic West Mound.[95,96]

Of the 471 individuals from primary burial contexts,[97] there are 178 adults (20+ years), 29 adolescents (12-20 years), 90 children (3-12 years), 67 infants (2 months-3 years), 85 neonates (0-2 months), and 22 prenates (> 38 weeks *in utero*). Among the adults and adolescents whose sex could be determined (n = 155), 89 individuals (57%) were assessed as females or possible females, while 66 individuals (43%) were assessed as males or possible males, a marginally significant difference (binomial test $p = 0.077$).

Description of Barcın Höyük

Located in the Yenişehir Valley in the province of Bursa in northwestern Turkey, the site of Barcın Höyük yielded an uninterrupted stratigraphic sequence from 6600 to 6000 cal BCE.[18] The settlement was built on a low natural elevation in what would have been a marshland valley.[98] The Neolithic levels at Barcın Höyük, which lie beneath a relatively thin deposit of later levels dating to the Chalcolithic, Bronze Ages and the Byzantine period, are thick and exceed 4.5 m in most places at the site. Called level VI, the Neolithic phase is divided into seven subphases: VIe (earliest level) through VIa. The VIe levels of the site represent the earliest farming community known to date in the Marmara Region.[18,19] The initial pioneer communities who arrived here around 6600 cal BCE brought with them crops to cultivate and animals to herd.[99,100] With regards to plants, domesticated varieties of cereals and pulses were plentiful.[101] Sheep and cattle were the preferred herd animals although goats were also present while hunting only contributed a minor part of the diet.[102,103] Extensive organic residue analyses on pottery demonstrate that the inhabitants of Barcın Höyük relied heavily on dairy products.[104] This observation confirms those made by Evershed and colleagues for later sites in the Marmara Region.[105]

The initial settlers in the region were accomplished potters even though pottery use was initially limited and indirect methods of heating foods were preferred.[106] Within a century however, thin-walled finely made burnished pots become plentiful. Building on a consistent tradition, recipes of manufacture and temper changed over the ensuing centuries.[106,107] The residents of Barcın Höyük lived in rectilinear timber frame houses with wood and mud walls.[18] Houses tended to be in rows, surrounded by courtyard areas where a variety of activities were carried out.

Burials associated with the settlement were placed within and near structures. Interestingly, many infants were buried within the house proper beneath floors while adults were typically placed in the courtyard areas. Children often tended to be buried outside but closer to the structures, sometimes beneath the floors of the verandas in front of the houses.

Although intensively analyzed for DNA,[22,23,108] the Barcın Höyük skeletons await final anthropological analyses. Based on preliminary data, adults appear to comprise 38% (46 burials) of the 121 burials that come from primary burial contexts.[109] Of the skeletons that can be identified based on sexual characteristics, nearly two thirds of these appear to be females or possibly females. Subadults including adolescents, children, infants and neonates comprise the remaining 62% of the assemblage.

Description of Tepecik-Çiftlik

Tepecik-Ciftlik is located in the Volcanic Cappadocia region of Central Anatolia in the Melendiz/Ciftlik Plain. The excavators suggest it was occupied from the end of the Aceramic Neolithic Period until the early Chalcolithic Period, between c.7500-5800 cal BCE.[17] The Pottery Neolithic levels show evidence of agriculture and animal breeding, as well as continued hunting and gathering. The site is in close proximity to major obsidian ore beds in the region and is notable for its large amount of obsidian tool remains. Further information about the site may be found at.[17]

A 2016 report on Tepecik-Ciftlik indicated that over 170 individuals' remains dating to the Neolithic levels, buried inside buildings and in open areas[20] had been excavated. A collective burial was also found, and is thought to have been used for successive burials, both primary and secondary.[110] It includes at least 42 individuals of both sexes and various ages.

Description of archaeological material

This section describes bioarchaeological characteristics of the individuals from Aşıklı Höyük, Çatalhöyük and Boncuklu Höyük. Some of this data are unpublished. Barcın Höyük and Tepecik-Çiftlik individuals included in this study have been described in the supplementary material of Mathieson et al.[23] and Kılınç et al.,[20] respectively.

Sex was estimated using dimorphic markers, and individual ages-at-death were estimated using standard methods such as human growth and epiphyseal fusion, dental calcification and bone maturity/size.[111] The sex of subadult individuals listed below have been determined based on genetic data produced in this study (Table Z2).

Description of Aşıklı Höyük individuals

SK2 (Level 1/2A; Building AB): the burial of a young adult female. Double burial. SK2 was buried in the same burial pit of a male, slightly later. The pit is located in a one-room rectangular building of the mid-8th millennium BCE settlement. Radiocarbon dating places the individual to 7585-7475 cal BCE (Table Z2).

SK33 (Level 2C, Building C): the burial of a child, buried under the floor of a rectangular planned *kerpiç* (mudbrick) building. Radiocarbon dating places the individual to 7945–7890 cal BCE (9%) or 7870–7595 cal BCE (86%) (Table Z2). Building C was renewed 10 times at the same location (Figure 3A),[112] where this child's burial was contemporary with its eighth renewal phase. Excavated in 1991.

SK40 (Level 2B, Building BH): the burial of an old adult female. Sub-floor inhumation in a rectangular kerpiç building of the 8th millennium BCE settlement. One of the three individuals buried in the same building: a one-month old infant and a middle adult female. Radiocarbon dating places the individual at 7935–7915 cal BCE (1%) or 7825–7590 cal BCE (94%) (Table Z2).

SK128 (Level 4, Building 3): the burial of a female child. She is one of the two individuals buried in the same building. Radiocarbon dated to 8225–7955 cal BCE (95%) (Table Z2).

SK129 (Level 4, Building 3): the burial of a young adult female, buried in a semi-subterranean oval building. She is one of the two individuals buried in the same building. Excavated in 2011; primary burial; radiocarbon dated to 8170–8115 cal BCE (6%), 8060–8045 cal BCE (1%), 8010–7985 cal BCE (1%), 7970–7735 cal BCE (86%) (Table Z2).

SK131 (Level 3E/4, Building 1): the burial of a female child, exposed lying on the pavement of a hearth in a semi-subterranean oval building. This is an exceptional burial, in position and in location. Four more individuals were buried in the same building. The burial was exposed in 2012. She was radiocarbon dated to 8200–8110 cal BCE (16%) or 8095–8035 cal BCE (7%) or 8015–7740 cal BCE (72%) (Table Z2).

SK133 (Level 3E/4, Building 1): the burial of an old adult female, the oldest member of the community thus far excavated. She was one of the five individuals buried in the same oval, semi-subterranean building, B.1. She was a primary burial and was radiocarbon dated to 8170–8115 cal BCE (8%), 8060–8040 cal BCE (1%), 8010–7980 cal BCE (2%), 7975–7735 cal BCE (84%) (Table Z2). Excavated in 2012.

SK136 (Level 3E/4, Building 1): the burial of a young adult female, one of the five individuals from Building 1. She was a primary burial, and was radiocarbon dated to 8175–8110 cal BCE (7%) or 8090–8075 cal BCE (1%) or 8065–8040 cal BCE (1%) or 8015–7705 cal BCE (84%) or 7695–7655 cal BCE (2%) (Table Z2). Excavated in 2015.

Description of Çatalhöyük individuals

Sk.5357 (burial feature 576, Level South K, Early period, Building 17): primary burial of a male infant. He was 9 months ± 3 months at death based on dental development. It was buried in a flexed position along the east wall of B.17 in association with red pigment and traces of reed basketry. The burial was excavated in 1999. Radiocarbon dating places this individual between 7035–6680 cal BCE (93%) or 6670–6650 cal BCE (2%) (Table Z2).

Sk.21855 (burial feature 8214, Level South K, Early period, Building 17): the primary burial of a female child. She was 4 years ± 1yr at death based on dental development. It was placed in a flexed position in a burial cut along the west wall of B.17. The burial was excavated in 2016.

Sk.1885 (burial feature 84, Level South M, Middle period, Building 50): the primary flexed burial of a male child. He was 7 years ± 2yrs at death, excavated in 1995. This individual was interred directly above Sk.2033 (see below) in the southwest corner of B.50. Radiocarbon dating places this individual between 6905–6885 cal BCE (1%) or 6825–6635 cal BCE (92%) or 6625–6600 cal BCE (2%) (Table Z2).

Sk.2033 (burial feature 84, Level South M, Middle period, Building 50): the primary flexed burial of a male child 3 years ± 1yr at death, excavated in 1995. This individual was interred directly below Sk.1885 (see previous) in the southwest corner of B.50. Radiocarbon dating places this individual between 6690-6590 cal BCE (95%) (Table Z2).

Sk.2017 (burial feature 96, Level South M, Middle period, Building 50): the primary burial of a female neonate (0-2 months at death based on measurements of the basi-occipital bone), excavated in 1997. The burial was located near the oven along the southern wall of B.50. The bones of this individual were scorched as a result of the burial's proximity to the oven. Radiocarbon dating places this individual between 6815–6790 cal BCE (2%) or 6775–6595 cal BCE (93%) (Table Z2).

Sk.2728 (burial feature 258, Level South M, Middle period, Building 50): an undisturbed primary burial of a female infant aged 9 months (±3 months) at death based on dental development. It was excavated in 1997 from Building 50, located in the South Area of the site. The body was placed in a small pit near the eastern wall of the main room. Radiocarbon dating of the petrous bone places this individual between 6695-6505 cal BCE (95%) (Table Z2).

Sk.2779.1 (burial feature 265, Level South M, Middle period, Building 50): the primary burial of a male neonate (0-2 months at death based on measurements of the basi-occipital bone), excavated in 1997. The burial was heavily disturbed by Mellaart's earlier excavations in this building during the 1960s.

Sk.2842 (burial feature 274, Level South M, Middle period, Building 50): a disturbed primary burial of a female infant aged 18 months (±6 months) at death based on dental development. It was excavated in 1998 from Building 50, located in the South Area of the site. The body was placed in a small pit near the center of the main room and was partially disturbed by a later burial. Radiocarbon dating of the petrous bone places this individual between 6690-6505 cal BCE (95%) (Table Z2).

Sk.21981 (burial feature 8153, Level South N, Middle period, Building 89): a disturbed primary burial of a female infant/child aged 3 years (±1 year) at death based on dental development. It was excavated in 2015 from Building 89, located in the South Area of the site. The body was placed in a small pit within the north platform of the main room and was subsequently truncated by the digging of a post retrieval pit.

Sk.5747 (burial feature 1064, Level South M, Middle period, Building 91): a primary burial of a female infant aged 18 months (±6 months) at death based on dental development. It was excavated in 2002 from Building 91, located in the South Area of the site. The body was placed in a small pit located in the northeast corner of B.91. Radiocarbon dating of the petrous bone places this individual between 6640-6490 cal BCE (95%) (Table Z2).

Sk.30006 (burial feature 7615, Level North G, Middle period, Building 114): a primary burial of a female infant aged 9 months (±3 months) at death based on dental development. It was excavated in 2015. The body was interred with a middle adult female in an oval pit along the south wall of the main room. Radiocarbon dating of the petrous bone places this individual between 6645–6495 cal BCE (94%) or 6490–6480 cal BCE (1%) (Table Z2).

Sk.8587 (burial feature 1013), Level North G, Middle period, Building 114): a primary burial of a female neonate (0-2 months at death – based on long bone length) excavated in 2002 and located under the southeast platform. The burial was partially disturbed by subsequent burials in this location, and likely also by rodent burrowing.

Sk.11739 (burial feature 1912, Level TP Q-R, Final period): a heavily disturbed set of human remains belonging to a middle adult (35-50 years of age-at-death) based on dental occlusal wear. The individual was assessed as a possible male based on cranial morphology, although aDNA suggested the individual was genetically female. These remains, potentially representing a secondary burial, were excavated in 2005 from Space 411, located in the TP Area of the site. Radiocarbon dating of the petrous bone places this individual between 6235-6075 cal BCE (95%) (Table Z2).

Sk.20217 (burial feature 3931, Level TP Q-R, Final period?): a female child aged 6 years (±2 years) at death based on dental development. This individual, excavated in 2012, is one of three individuals recovered from burial feature 3931 in the TPC Area. The burial was badly damaged as it was found directly beneath the surface. Hence, it could not be associated with any Neolithic buildings or spaces. Its stratigraphic position indicates that it post-dates B.122 from the Late period, which implies it most likely comes from the Final period. However, this is not corroborated by radiocarbon dating of the petrous bone that places this individual significantly earlier, between 6415-6240 cal BCE (95%) (Table Z2).

Description of Boncuklu Höyük individuals

ZHAJ (Area H, Grave 27): this is a primary single inhumation of a middle adult female (as determined by aDNA) buried in a sub-oval cut. The individual was found lying tightly flexed on her left side, positioned east-west with the head toward the west and facing north.

ZHAG and ZHAF (Area H, Grave 18): grave 18 contained a double inhumation of a middle adult female (ZHAF) and a perinatal infant (ZHAG) found in an oval cut larger than average. The adult (ZHAF) was found lying tightly flexed on her left side and positioned with a northwest-southeast orientation with the head toward the northwest. The perinate was articulated and found with the head on top of the adult pelvis. The female sex of the adult could be confirmed by ancient DNA.[20] The sex of the perinate was determined as a female by aDNA, and it can be ruled out that ZHAF and ZHAG were first or second-degree related. Skeleton ZHAF has been radiocarbon dated to 8285–8175 cal BCE (83%) or 8115–8090 cal BCE (4%) or 8040–8010 cal BCE (8%) (Table Z2).

ZHB (Area H, Grave 9): a single inhumation of an adolescent-young adult female. The sex of the individual has been confirmed by ancient DNA analysis.[20] The individual was found lying on her right side/partially prone, in a semi-flexed position. The body was

orientated east-west with head to the east and facing northeast, and has been radiocarbon dated to between 8280–8165 cal BCE (57%) or 8120–7960 cal BCE (38%) (Table Z2).

ZHF (Area H, Grave 14): single inhumation of an adult male buried in a sub-oval cut. The age-at-death of the individual was difficult to estimate accurately because both the skull and pelvis were highly fragmented. The sex has been confirmed by ancient DNA.[20] The body was found lying on the left side and orientated northwest-southeast with the head orientated toward the northwest and facing northeast. The upper limbs were flexed at the elbow with the palms of the hands together and placed immediately in front of the face. The long bones were highly fragmented and animal burrowing had destroyed much of the skull, most of the axial elements and the feet. The skeleton has been radiocarbon dated to 8225–7940 cal BCE (95%) (Table Z2)

ZHJ (Area H, Grave 15): this is a primary single old adult inhumation found in a sub-oval cut. The individual was found in a flexed position lying on its right side and positioned north-south with the head orientated toward the south. The bones were relatively well preserved compared with other graves, although burrowing animals had destroyed parts of the skull and axial skeleton, including the left foot. Morphological sex determination was difficult because the remains were gracile, probably as a result of the aging process. Ancient DNA analyses demonstrated that this individual was female. She has been radiocarbon dated to 8295–8240 cal BCE (95%) (Table Z2).

ZHBJ (Area H Grave 30): single inhumation of a middle/old adult male in a suboval cut. Sex has been confirmed by ancient DNA.[20] The individual was found lying tightly flexed on his right side, although it should be noted that there was considerable damage from bioturbation that disturbed much of the skeleton and destroyed most of the thorax and skull. The body was positioned east-west with the head toward the west, but the facing direction could not be ascertained due to the aforementioned disturbance.

ZKO (Area K, Grave 12): this is a single inhumation of an old adult male in an oval cut. The individual was found lying tightly flexed on his left side and orientated east-west with the head toward the east. The bones were generally well preserved, but rodent burrowing activity caused significant disturbance of the ribs, scapulae and vertebrae. Sex was confirmed through aDNA analysis as male.

ZMOJ (Area M, Grave 49): a primary but heavily disturbed burial of a young adult male (determined by aDNA) in a sub-circular grave. The individual was orientated east-west with head to the west and facing north. The skull was found at one end of the grave and many of the other bones had been moved by animal action, so their anatomical position was not maintained. Ancient DNA indicates that this individual was male.

METHOD DETAILS

Radiocarbon dating

Radiocarbon measurements have been obtained on remains from a total of eighteen individuals, eight from Aşıklı Höyük and ten individuals from Çatalhöyük as part of this study. Fourteen samples were dated at the TÜBİTAK-MAM facility (Gebze, Turkey) in 2019, three at the Oxford Radiocarbon Accelerator Unit in 2009, 2016, and 2018, three at the Keck Carbon Cycle AMS Facility, University of California (Irvine) in 2014 and 2018, and three at Uppsala University in 2018. At TÜBİTAK-MAM and Uppsala University samples obtained from petrous or rib bones of each individual were dated. At Oxford and Irvine samples were processed from lower limb bones (femora or tibiae). At TÜBİTAK-MAM the human bone samples were gelatinised and ultrafiltered,[113] graphitised[114] and dated by AMS on a 1MV NEC Pelletron accelerator. At Oxford the samples were also gelatinised and ultrafiltered,[115] graphitized,[116] and dated by AMS.[117] At Irvine samples were also gelatinised and ultrafiltered,[113,118] combusted,[119] graphitized,[120] and dated by AMS.[121] In Uppsala the samples were gelatinized,[122] combusted and graphitized,[123] and dated by AMS.[124] The results are conventional radiocarbon ages[125] and have been corrected for fractionation using d^{13}C values measured by AMS. Two samples from Çatalhöyük (30006 and 5747) were dated at both the TÜBİTAK-MAM facility and the Oxford Radiocarbon Accelerator Unit, and in both cases produced measurements that are not statistically different at the 5% significance level[126] (Table Z2). The three individuals dated at Uppsala University were also dated at the TÜBİTAK-MAM facility, again producing pairs of results that were not statistically different at the 5% significance level[126] (Table Z2).

Radiocarbon ages were calibrated using *IntCal20*[127] and the probability method[128] with ranges rounded outward to 5 years; replicate measurements have been combined by taking a weighted mean before calibration[126] (Tables 1 and Z2). The remaining sample ages were inferred based on their archaeological context.

Molecular biology laboratory methods

Sample preparation and DNA extraction

Sample preparation and DNA extraction were performed using two different protocols. In the first protocol, sample preparation, DNA extraction and library preparation from Aşıklı Höyük and Çatalhöyük samples were carried out in a dedicated laboratory for ancient DNA analysis at the Middle East Technical University (METU) (Ankara, Turkey). All necessary precautions to avoid contamination were taken during the sample grinding, DNA extraction and library preparation processes: tools and surfaces were regularly cleaned with bleach, RNase AWAY and long exposures with UV light in the aDNA laboratory. Samples were decontaminated and prepared as in:[129] The outer surface of the samples were carefully removed and discarded using either single use blades or Dremel drill with single use cutting discs. Each side of a sample was irradiated with UV-light (254 nm wavelength, 12 V and a distance of 5 cm from the UV source) in a cross-linker for 30 min. The samples were ground into fine powder using a freezer mill. DNA extraction was performed from 120-200 mg bone powder using a silica spin column method following Dabney et al. (2013)[130] with slight modifications from Ottoni et al. (2011).[131] Briefly, bone powder from the inner part of petrous bones were digested in 1 mL extraction buffer (0.45 M

EDTA, 0.25 mg/mL of Proteinase K, pH 8.0) h by vortexing and leaving in a rotating shaker at 56 þC for 24 h and then 37 þC for another 24. The supernatant was then transferred to an extension reservoir (Zymo Research) and fully mixed with the 13 mL custom binding buffer (5 M guanidine hydrochloride, 40% Isopropanol, 90 mM sodium acetate and 0.05% Tween-20), which is fitted to MinElute silica spin column (QIAGEN). First, extension reservoir -MinElute assembly was placed into a 50 mL falcon tube and centrifuged for 4 min at 1,500 x g, then rotated 90þ and centrifuged for another 2 min at 1,500 x g. Collection tubes with MinElute spin column are centrifuged (dry spin) for 1 min at 3,300 x g followed by two washes with PE buffer (MinElute, QIAGEN). The DNA was then eluted twice in 50 mL of Elution Buffer (EB) (MinElute, QIAGEN) with 0.05% Tween-20 and centrifuged at 16,100 x g for 1 min to collect DNA. Two negative extraction controls accompanied every 8-10 samples.

In the second protocol, one sample (21981) from Çatalhöyük was processed in the laboratory dedicated to ancient DNA analyses, at the Institute of Human Biology and Evolution, at the Adam Mickiewicz University in Poznań (AMU) (Poland). Similar precautions to avoid contamination were taken as in the METU laboratory. In addition, prior to DNA extraction, the sample was cleaned with 2% NaOCl and rinsed with sterile water. After cleaning, the sample was UV irradiated for at least one h on each side. Following UV irradiation, the petrous bone was cut in half and drilled with the single use Dremel cutting disc and drill bit. Then DNA was extracted from 150 mg of bone powder following a silica-based method developed by Yang et al.[132] with modifications introduced by Svensson et al.[133] Briefly, the bone powder was digested in 1.5 mL extraction buffer (0.4 M EDTA, pH 8.0), 1 M Urea and 15 μl of Proteinase K) for 24 h in a rotating shaker at 56þC. The obtained solution was than concentrated to 100 mL using Amicon Ultra-0.5 Centrifugal Filters. The DNA was then extracted from the concentrated solution using QiaQuick PCR purification kit (QIAGEN) following the manufacturer protocol, where final elution was performed twice in 55 mL of EB buffer.

UDG-treatment was not applied on any sample.

Library preparation and initial sequencing

Double-stranded DNA libraries were prepared using 20 mL of DNA extract using blunt-end ligation method following Meyer et al. (2010)[134] with modifications as in Günther et al. (2015).[135] Each library was amplified via PCR in six replicates, each in a total volume of 25 ml, using specific indexing primers (15 single- and seven double-indexing) (Table Z1). Negative controls were included in both the library preparation and PCR steps. Each reaction contained 3 mL DNA library and the following in final concentrations; 1X AmpliTaq Gold Buffer, 2.5 mM MgCl2, 250 nM of each dNTP, 2.5U AmpliTaq Gold (Life Technologies), and 200 nM each of the IS4 primer and an indexed P7 primer. The cycling conditions were 94°C for 10 min followed by 10-14 cycles of 94°C for 30 s, 60°C for 30 s, 72°C for 45 s and a final extension at 72°C for 10 min. Amplified libraries were pooled and purified with AMPure XP beads (Agencourt). The libraries were quantified on a 2100 Bioanalyzer using the High Sensitivity DNA Kit (Agilent Technologies) or on Tapestation 2200 (High Sensitivity D1000 ScreenTape). None of the extraction blanks or PCR blanks showed presence of DNA and were therefore not sequenced. Libraries were pooled in equimolar concentrations (final vol of 10 nM total pool) for the initial sequencing (prescreening) process and sequenced on Illumina HiSeq 2500, HiSeq X and NovaSeqS4 platforms at SciLife, Stockholm, with 100-150 bp paired-end reads on single or several lanes. Libraries that yielded sufficient reads from the initial screening process were then sequenced deeper in pools of three to six libraries per lane.

Whole genome in-solution capture and resequencing

To increase the depth of coverage, the best libraries of 11 individuals (Table Z1) -in terms of human DNA proportions in the prescreening data- we enriched for human genomic DNA using the MYbaits Human Whole Genome Capture Kit (African baits) from Arbor Biosciences (Ann Arbor, MI) following the manufacturer's instructions (http://www.mycroarray.com/pdf/MYbaits-manual-v4.pdf). PCR was performed for each captured library at 16-19 cycles with primers IS5 (5¢AATGATACGGCGACCACCGA) and IS6 (5¢ AAGCAGAAGACGGCATACGA) using either Herculase II Fusion DNA Polymerase (Agilent Technologies) or KAPA HiFi HotStart Polymerase (Kapa Biosystems). Captured libraries were purified with AMPure XP beads and quantified on the Bioanalyzer 2100 using the High Sensitivity DNA Kit (Agilent Technologies). Purified libraries were pooled in equimolar concentrations and sequenced on Illumina Hiseq 2500 and Hiseq X platforms at SciLife, Stockholm, with 100-150 bp paired-end reads on one or several lanes. The enrichment procedure increased human DNA endogenous proportions by 1.2× −27× (median 8×) (Table Z1).

QUANTIFICATION AND STATISTICAL ANALYSIS

Sequence read processing

We processed sequencing data from each library as described in Kılınç et al.[20] We trimmed the residual adaptor sequences in *FASTQ* files and merged the paired-end sequencing reads using *MergeReadsFastQ_cc.py*[136] or *AdapterRemoval*,[137] with an overlap of at least 11 bp between the pairs. We mapped the merged reads with single-ended mode to the human reference genome (version hs37d5) using *BWA aln* (version 0.7.15)[138] with the parameters *"-n 0.01 -o 2"* and disabled the seed with *"-l 16500"*[139,140]. We merged different libraries of the same individual and removed PCR duplicates, collapsing the reads with identical start and end positions using *FilterUniqueSAMCons.py*.[136] Finally, we filtered the reads shorter than 35 bp length and more than 10% mismatches to the human reference genome.[20] We also remapped published ancient data from Table Z3 following the same procedures for comparative analysis.

Authentication of data, contamination estimates and molecular sex determination

We estimated the authenticity and level of contamination in the ancient genomes using three different approaches: (a) studying the post-mortem damage (PMD) patterns that are characteristic of ancient DNA, (b) checking for mtDNA contamination based on the presence of heterozygous sites, and (c) ruling out X chromosome contamination in male individuals.

Postmortem damage

We assessed the authenticity of all samples from Aşıklı Höyük (n = 8) and Çatalhöyük (n = 14) by estimating aDNA-specific damage patterns represented by high frequency cytosine to thymine (C to T) transitions at 5¢ ends of reads, and guanine to adenine transitions at the 3¢ ends. We used *PMDtools*[141] to evaluate the frequency of PMDs at the first 30 positions at the 5¢- and 3¢ ends of the reads. All individuals' libraries showed > 25% PMD at 5¢- and 3¢ ends (Figure S1A; Table Z1).

Mitochondrial contamination

We calculated mitochondrial DNA (mtDNA) contamination of all samples from Aşıklı (n = 8) and Çatalhöyük (n = 14) using *contamMix* (version 1.0.10).[142] This method calculates posterior probability of mtDNA contamination using a Bayesian approach. We called a consensus mtDNA sequence for each individual from BAM files using *samtools mpileup* (version 1.9)[143] and *vcfutils* modules. Then, the consensus sequences were added to a set of 311 modern human mtDNA sequences and mapped to these 311 modern human mtDNA sequences. Here, we calculated contamination rates with and without transitions using *contamMix*. Authenticity was estimated at ^{3}93% for all 22 libraries (Table Z1).

X chromosome contamination

X chromosome contamination was estimated for the male individuals from Aşıklı (n = 1) and Çatalhöyük (n = 4) using *ANGSD*.[144] We generated a binary chrX *BAM* file with the following parameters *"-r X:5000000-154900000 -doCounts 1 -iCounts 1 -minMapQ 30 -minQ 30"* for X chromosome positions. Then, we ran the *contamination.R* script to estimate chrX contamination using Fisher's exact test and the jackknife procedure (Table Z1).

Overall evaluation of aDNA authenticity

Evaluating the above results together, we confirmed that all n = 22 individuals examined passed at least two of the contamination estimation methods.[108] Given these results, we included all samples in further analyses.

Molecular sex determination

To assess the molecular sex of all individuals, we calculated the ratio of reads mapping to the Y chromosome to mapping to both X and Y chromosomes using the *Ry* method as described in (Skoglund et al., 2012, 2013)[145,146] with mapping quality of at least 30. One Aşıklı and four Çatalhöyük individuals were identified as males. Seven Aşıklı and ten Çatalhöyük individuals were assigned as females. Our molecular sexing results are consistent with the osteological analysis (Table Z2).

Mitochondrial DNA and Y chromosome analyses

Mitochondrial DNA

We obtained mitochondrial genomes with mean coverages between 0.4- and 258-fold per individual (Table Z1). In order to assess the mitochondrial haplogroups, we called consensus mitochondrial sequences of each individual using *samtools* (version 1.9) *mpileup* and *variant caller tools*[143] with parameters set for aDNA; namely, filtering for sites that have a minimum depth of 3 and a base and mapping quality score of at least 30.[147] We assigned mtDNA haplogroups of each individual based on SNPs at informative nucleotide positions of the mitochondrial genome using *HaploGrep2v2.1.1* (https://haplogrep.i-med.ac.at/).[148]

The results are presented in Table Z2 and Figure S1E. We observed haplogroup K1a4, one of the common haplogroups in Neolithic farmer populations,[149] in three individuals from Aşıklı (128, 129, 133). Two Aşıklı individuals (131, 136) belonged to T2c1a and the remaining three individuals from Aşıklı (2, 33, 40) belonged to haplogroups H2a2a, U3a and N1a1a1, respectively. We also found haplogroup K1a4 in one Çatalhöyük individual (30006) and a subtype of haplogroup K1 (K1a) in three individuals (2728, 2842, 1885). Three Çatalhöyük individuals (21981, 11739, 20217) belonged to three subtypes of K1 (K1a17, K1b1, K1a4b), respectively. Three individuals from Çatalhöyük (8587, 2017, 5747) belonged to subtypes of T2 (T2e, T2, T2c1) and one (5357) belonged to N1a1a1, one of the most abundant haplogroup in Near Eastern and European farmer populations.[149,150] The remaining Çatalhöyük individuals (2033, 2779, 21855) belonged to subtypes of H2a2a (H2a2a1d, H2a2a, H2a2a1), respectively.

Y chromosome

We used the *yHaplo* program (version 1.0.19)[151] to assign Y chromosome haplogroups of one Aşıklı and four Çatalhöyük male individuals. We genotyped each male individual based on 13,508 ISOGG (International Society of Genetic Genealogy, http://isogg.org, version 11.04) consortium SNPs, excluding strand-ambiguous SNPs (C/G and A/T) and by randomly choosing one allele for each of 13,508 ISOGG SNPs. We called all single base substitutions using *BAM* files mapped to hs37d5 and *samtools mpileup* (version 1.9)[143] (filtering the sites with a mapping quality and a base quality of lower than 30. We also excluded insertions/deletions and sites that displayed multiple alleles.

We observed haplogroup G2a2b in Aşıklı33 male individual. Two individuals from Çatalhöyük (5357 and 2779) were assigned to haplogroup C1a2 and the remaining two Çatalhöyük individuals (1885 and 2033) belonged to haplogroups, G2a2a1 and H3a1, respectively (Table Z2).

Dataset processing

Reference SNP datasets

We prepared three datasets by merging the ancient genomes generated in this study (n = 22) with published ancient genomes from previous studies (n = 191; Table Z3) and with different datasets of modern-day populations:

DS1: The Human Origins SNP Array dataset[25,139] that includes 594,924 autosomal SNPs (both transitions and transversions) genotyped in 2,730 modern-day individuals from 203 populations. This SNP list was used for PCA and *ADMIXTURE* analyses. When genotyping using DS1, transitions were included only conditionally (see 'Genotyping' below) to avoid the influence of postmortem damage.

DS2: The 1000 Genomes whole genome sequencing dataset (phase 3)[152] comprised of 1,938,919 biallelic autosomal transversion SNPs genotyped in West African Yoruba (YRI) individuals (n = 108) was used to maximize the genetic overlap between ancient samples and reduce the potential effect of post-mortem damage. The dataset was prepared by extracting all transversion SNPs and choosing alleles with minor allele frequency ³10% in Yorubans to avoid the effect of Eurasian admixture into Yorubans, as described in (Günther et al., 2015; Kılınç et al., 2016).[20,135] This dataset was used for outgroup f_3 and *D*-statistics, F_{ST} estimation, ROH analysis and also for kinship estimation analysis. For qpAdm analyses, we merged DS2 with SGDP v4[153] (Simons Genome Diversity Project, downloaded from https://reichdata.hms.harvard.edu/pub/datasets/sgdp/) by overlapping 1,479,034 SNPs.

DS3: X chromosome SNPs from the 1000 Genomes whole genome sequencing dataset version (phase 3)[126] that were genotyped in West African Yorubans were extracted and those with minor allele frequency ³10% were chosen (across n = 56 female and n = 52 male Yoruba individuals). We also removed pseudoautosomal regions from X chromosomes as described in the human reference genome (hs37d5). We included only transversion SNPs in this dataset. The resulting 73,799 SNP list was used for X chromosome-based kinship estimation.

Genotyping

For each of the described SNP datasets, we genotyped ancient individuals at each SNP using reads with minimum base quality and mapping quality of 30.

We pseudohaploidized our data, such that when multiple reads overlapped with the same SNP we randomly selected one read and this position was assumed to be homozygous in the ancient individuals.[20,154] We removed the non-biallelic sites, transitions and indels including the sites that were found in an ancient individual but not in the reference data.

To avoid the influence of postmortem damage, whenever we encountered a T or an A when the reference genome carried a C or a G, respectively, we coded that position as missing in the ancient genotype, following.[20,135]

For kinship estimation using *NgsRelate* we used the *ANGSD* program (see section '*NgsRelate* analysis' below).

Population genetic analyses

In all population genetics analyses we excluded related individuals, by choosing the individual with the highest genome coverage among groups of closely related individuals (> 3rd degree).

Principal components analysis

We carried out principal component analysis (PCA) as a first assessment of the genetic affinities of the newly generated genomes from Aşıklı and Çatalhöyük following.[20] First, we conducted PCA using a total of 49 modern-day West Eurasian populations from the Human Origins SNP Array dataset[25,139] and projected the 136 ancient individuals (114 previously published and 22 reported in this study) onto the first two principal components inferred from modern individuals. The list of modern-day populations is presented in Figure S3B. For this, the *smartpca* program in the *EIGENSOFT* package[155] was used with the parameters *"numoutlieriter:0"* and *"lsqproject:YES"*. We used dataset DS1 for Figure 1B. We further repeated the PCA also using transversion SNPs only (Figure S3C).

f_3- and D-statistics

To investigate pairwise genetic affinity between populations or between individuals, we computed outgroup f_3-statistics using DS2 (autosomal data) with the *qp3Pop* program in the *ADMIXTOOLS* package.[25] This quantifies shared drift between individuals/populations as their divergence from an outgroup population.[25] The calculation was performed at both the individual and the population levels. The Yoruba (YRI) population from 1000 Genomes Project phase 3[152] was used as an outgroup.

To study population-level similarities, we converted f_3-statistics into a pairwise distance matrix by subtracting all values from 1; we then summarized this distance matrix on two dimensions using multidimensional scaling (*MDS*) with the *'cmdscale'* function in the *R 'stats'* package (http://www.r-project.org/) (Figures 1C and S2A). We computed the *MDS* goodness of fit by calculating a new distance matrix from the *MDS* output and calculating its Pearson correlation coefficient with the original distance matrix.

These outgroup f_3-statistics were used for 3 different purposes: (a) studying inter-population relationships (Table Z5), (b) comparing inter-individual genetic diversity within populations (see below) (Table Z8), (c) studying correlation between burial location and genetic distance.

We calculated *D*-statistics for two purposes. First, we tested whether pairs of individuals from a Neolithic site systematically formed clades (showed a higher degree of allele sharing with each other) relative to individuals from other sites (Table Z7; Figures S3D–S3F). Second, we tested population-level affinities between and among Neolithic Anatolian populations (all individuals sampled from a site) and other broadly contemporaneous populations and to infer admixture events (Tables Z3 and Z4). For *D*-tests we used the program *qpDstat* in the *ADMIXTOOLS* package, and used the Yoruba population as outgroup. Statistical significance and the confidence intervals were estimated using the block-jackknife procedure by *ADMIXTOOLS*. Confidence intervals shown in plots display ± 2 standard errors from the mean. We used a cutoff of $|Z|^3 3$ for nominal statistical significance but did not apply multiple testing correction, due to the descriptive nature of the analyses.

We conducted additional individual-level *D*-statistics (a) only using shotgun data for Boncuklu to avoid the influence of technical biases that may arise due to use of capture and shotgun data, (b) comparing only Boncuklu and Aşıklı Höyük data to directly test possible structure within Aceramic Central Anatolia (Figures S3E and S3F).

Technical influence on f_3-statistics

Because our dataset includes genomic data produced by different laboratory procedures, including shotgun sequencing and 1240K SNP capture, we explored the possible influence of technical effects on outgroup f_3-statistic estimates, taking advantage of the fact

that our dataset contained both types of data for individuals from Boncuklu,[20,21] and that Boncuklu Höyük shows high genetic homogeneity within the settlement (see below). We found that f_3-statistics that include pairs of individuals with genomic data produced using alternative procedures are only slightly lower than f_3-statistics that include pairs of individuals produced using the same procedure, when autosomal data is included (Figure S1D). On the other hand, calculating the same statistics with X chromosomal data, we observed a conspicuous technical effect (Figure S1D). Accordingly, we did not analyze X chromosomal genetic diversity in the following analysis.

Within-group genetic diversity comparisons

We analyzed genetic diversity differences among groups, either comparing (a) populations, represented by all individuals sampled per settlement, or (b) subsets of individuals from specific phases of settlements. Diversity per group was calculated as the mean of 1- f_3 (see above) among all members of a group (Table Z8). To test the statistical significance of differences in diversity between 2 groups, we used a random permutation test. Specifically, to calculate the null distribution for the diversity difference between 2 groups, we permuted group membership randomly using the *R "sample"* function, we then calculated the mean of 1- f_3 values among all members of each pseudogroup, and calculated the absolute difference of these means (Table Z9). This was repeated 10,000 times to create a null distribution. Finally, we compared the observed absolute mean diversity differences to the null expectation, yielding an empirical *P*-value for each pair of compared groups. Note that permutation tests can be convenient for testing null hypotheses of no difference between groups, but they are also conservative because the null distribution can include the observed difference especially when the sample size is small.

ADMIXTURE analysis

We performed unsupervised genetic clustering using *ADMIXTURE*[24] software to estimate ancestry components in ancient genomes produced in this study. We used present-day Eurasian, African, Asian, and American groups' genotype data from the Human Origins dataset (n = 231)[25,139] and merged this with the ancient individuals' genotypes. We filtered the dataset by pruning for linkage disequilibrium using *PLINK*[156] with the parameters *"–indep-pairwise 200 25 0.4"* and for missing genotype with *"-geno 0.99"*, leaving 518,401 SNPs to be analyzed. We conducted *ADMIXTURE* analysis for each value of *K* ranging from 2 to 15 and determined the clusters of each ancient individual using the *"projection"* function of *ADMIXTURE*. We visualized the results using *PONG* software.[157]

F_{ST} estimation

We computed pairwise F_{ST} to evaluate genetic differentiation among early Holocene populations from Anatolia, Levant and Iran. Population-level F_{ST} were calculated for ancient population samples that included at least two ancient individuals - the same criterion as used for f_3-statistics-based population comparisons. The *smartpca* program in the *EIGENSOFT* package[155] was used with default parameters *"inbreed:YES"* and *"fstonly:YES"*, and with DS2 (autosomal data). We visualized the results on two dimensions using multidimensional scaling (*MDS*) with the *'cmdscale'* function in the *R 'stats'* package (http://www.r-project.org/) (Figure S2B; Table Z6).

qpWave/qpAdm admixture analysis

We modeled target Anatolian Ceramic period populations (Çatalhöyük and Barcın) as admixture between two or three source populations using the *qpWave* (v1200) and *qpAdm* (v1201)[158] programs in *ADMIXTOOLS* (v7.0),[25] with the option "allsnps: YES." We used a basic set of 12 outgroups as "right populations," which included the present-day individuals (n = 28); Mbuti,[153,159] Han,[153,159] Papuan,[159–161] Onge,[153] Chukchi,[153] Mixe,[153,159] as well as the ancient individuals (n = 24); Kostenki14,[162] Mal'ta,[162] Villabruna,[162–164] Natufian,[108] Caucasian hunter-gatherers (CHG)[162,163] and Pınarbaşı[21] (collectively referred to as "Base" in Table Z10). These outgroups were chosen by considering their geographical remoteness, and lack of recent backflow from the selected target and source populations.[21,165] Our source populations were Anatolian Aceramic (Aşıklı or Boncuklu), Levant Neolithic and Iran Neolithic, which were used as "left populations" (Table Z10). We used a significance level threshold of p = 0.05 to reject models for both *qpWave* and *qpAdm* analysis.

Results of *qpWave* showed that neither the Aşıklı versus Boncuklu populations could be distinguished from each other, nor Çatalhöyük versus Barcın populations (p > 0.01), a result expected given their similarities observed in other analyses (e.g., MDS). We could model the latter Ceramic Neolithic populations (Çatalhöyük or Barcın) as mixtures of Aceramic Neolithic (Aşıklı or Boncuklu) and Levant Neolithic (*P*-value > 0.05; Table Z10). However the fit was poor when we tried to model Ceramic Neolithic populations (Çatalhöyük or Barcın) as mixtures of Aceramic Neolithic (Aşıklı or Boncuklu) and Iran Neolithic (mixture proportions > 1 and/or *P*-value < 0.05). Thus, in a second round of analyses, we tested whether CHG could be a better proxy for a possible eastern source of gene flow. For this we removed CHG from the "right populations" list and used it instead of Iran Neolithic among the "left populations" (Table Z10). This likewise produced poor fits.

Estimating genetic relatedness and pedigree relationships

Overview of kinship analyses

We first inferred relatedness coefficients for pairs of Aşıklı and Çatalhöyük individuals and previously published Anatolian Neolithic individuals. For this we used three different software, *NgsRelate*,[35] *lcMLkin*,[36] and *READ*,[37] as described below. *NgsRelate* and *lcMLkin* infer the genetic kinship coefficient (θ) between a pair of individuals, i.e., the probability that a pair of randomly chosen alleles from two individuals each are identical-by-descent (*IBD*). For this they first estimate k_0, k_1, k_2 (Cotterman coefficients), which are the probabilities of sharing 0, 1 and 2 alleles *IBD* between a pair of individuals, such that $k_0 + k_1 + k_2 = 1$. The kinship coefficient θ is then calculated as $\theta = k_1 / 4 + k_2 / 2$. *READ* uses an alternative approach, described below. All 3-software produced consistent results.

CellPress
OPEN ACCESS

We restricted the kinship analyses to estimates of 1st-3rd degree and to a minimum threshold of 5,000 overlapping SNPs between a pair of individuals. This was based on the empirical observation that the software sometimes estimated non-0 kinship coefficient values for pairs of individuals with < 5,000 SNPs overlapping between them, and who could not possibly be genetically close relatives. For instance, *NgsRelate* calculates the kinship coefficient (θ) between Aşıklı 2 and Barcın M10_352, sharing 3,044 SNPs, as 0.02, suggesting a 4th-5th degree relationship, despite the pair being separated by c.1,000 years, i.e., about 40 generations (Figure S1B; Table Z11). In another example, the kinship coefficient calculated by *NgsRelate* between Aşıklı 2 and Aşıklı 40 was estimated as 0.71 by *NgsRelate,* both with low quality data and sharing 1,662 SNPs, suggesting an inbred 1st degree relationship pairs, despite more than 300 year between two individuals (Figure S1B; Table Z11).

All analyses were run on the autosomal transversion dataset DS2 and using *BAM* files as input data for all three software. In addition, we also implemented *NgsRelate* on DS3 to estimate X chromosomal kinship coefficients.

NgsRelate analysis

NgsRelate software (version 2)[35] is designed to work efficiently on low-coverage genomic data, and uses genotype likelihood estimates instead of genotype calls. It also uses background population allele frequencies in θ estimation, which is based on maximum likelihood using expectation maximization. According to Hanghøj and colleagues, it accurately estimates genetic relatedness between pairs of individuals down to 5th degree (e.g., second-degree cousins). Importantly the software also estimates the inbreeding coefficient per individual simultaneously, which distinguishes it from *lcMLkin*.

We ran *NgsRelate* using *BAM* files as input with default parameters. We used genotype likelihoods calculated by the *ANGSD* program[144] with population allele frequencies calculated from n = 60 Anatolian early Holocene individuals (Tables Z2 and Z3). We computed ten replicate runs for each biologically related pair using autosomal data (Figure 2B).

lcMLkin analysis

lcMLkin[36] also implements a maximum likelihood approach with expectation maximization, uses genotype likelihoods and population allele frequencies. The authors suggest it can accurately estimate genetic relatedness down to 5th degree (e.g., second-degree cousins) in low-coverage data. As opposed to *NgsRelate, lcMLkin* assumes no inbred individuals. We ran *lcMLkin* with population allele frequencies calculated from n = 60 Anatolian early Holocene individuals (Tables Z2 and Z3) including the newly generated data (n = 22) and using default parameters.

READ analysis

As a third approach, we used the software *READ*[37] which applies a non-parametric approach to estimate biological kin-relationship between pairs. *READ* calculates and normalizes the mismatch rate (non-matching alleles, *P0*) in non-overlapping windows of 1 Mbps across the whole genome using pseudo-haploid data. *READ* uses this value to infer the degree of relationship (first degree as immediate family; parent-offspring and siblings, and second degree as extended family; cousins, uncles/aunts, grandparent-grandchild, and half-siblings), but cannot detect more distant relatives than estimating relatedness down to 3rd degree. *READ* compares the *P0* distribution of each pair to those average unrelated pairs.

We implemented *READ* on each population separately, i.e., with all individuals from each Anatolian Neolithic site as background, with default parameters. For each pair, we used normalized 1-*P0* values as a proxy for the kinship coefficient (θ), shown in Figure 2A. Here, normalization is done using the median *P0* across all pairs, which represents the mismatch level of an average unrelated pair.

The effect of population background on θ estimates

In *NgsRelate* and *lcMLkin* analyses we used all 60 Anatolian Neolithic individuals (Tables Z2 and Z3) to calculate population allele frequencies. Here, the relatively high genetic homogeneity of Aşıklı Höyük and Boncuklu Höyük relative to the 3 Ceramic Neolithic sites (Figure 1D) could theoretically lead to overestimation of θ among pairs from Aşıklı Höyük and Boncuklu Höyük. However, we do not expect that the magnitude of this effect could create artificially high θ values consistent with 1st degree relatedness. In addition, in *READ* analysis we used each site's population as background, and the consistency among results of the 3 software suggests that high genetic homogeneity of Aşıklı Höyük and Boncuklu Höyük does not confound the results.

Pedigree relationships

In addition to the genetic relatedness level, we further inferred the most likely pedigree relationship between close relatives. This is a more challenging task because the Cotterman coefficient estimates from aDNA data are highly noisy, as evident from our simulations. This is not surprising as our genome coverages rarely exceed 1X, while Cotterman coefficients describe the probabilities of IBD across 4 alleles of 2 individuals at diploid loci. We therefore used multiple sources of information in combination: Cotterman coefficients, the ratio between X chromosomal θ and autosomal θ, the anthropometric age-at-death estimates, radiocarbon dates, and mitochondrial and Y chromosomal haplotype information. We further used pedigree simulations to best assess the results.

Biological kinship estimation on the X chromosome

We estimated the kinship coefficient from X chromosome loci in order to distinguish different pedigree relationships (e.g., siblings, mother-son, father-daughter) among putative first-degree pairs (Tables S1, S3 and Z11). For this, we used *NgsRelate* software[35] and X chromosome SNP data from 1000 Genomes genotyped in African Yorubans, following the same method described in previous sections. We restricted the X chromosome-based kinship estimates to a minimum threshold of 800 overlapping SNPs between a pair of individuals. We then compared the ratio between autosomal and X chromosome θ for each pair.

Testing mtDNA homogeneity for resolving pedigree relationships

We tested mtDNA homogeneity for estimating pedigree relationships among Boncuklu Höyük individuals using pairwise mismatch rate of mtDNA sequences, since the Boncuklu Höyük population is genetically homogeneous. For this, we called consensus mtDNA sequences using the method described earlier and calculated the pairwise mismatch rate between mtDNA consensus sequences of

each individual pair following.[135,141] We find that both first-degree related Boncuklu Höyük pairs, despite sharing the same mtDNA haplogroup with other Boncuklu Höyük individuals, have the lowest mismatch rates (Table Z14). This suggests they might actually share the same haplotype and the observed mismatches could be attributed to sequencing error. If so, combined with the other evidence (Tables S2, S3 and Z14), we infer the most likely pedigree relationship for the Boncuklu ZHF-ZHJ pair as mother-son.

Goodness-of-fit tests

We performed goodness-of-fit tests on contingency tables for Figure 2D and for the data in Table Z13, using a two-sided Fisher's exact test as implemented in the R "stats" package. For testing the frequency of sisters in Neolithic Anatolia versus Bronze Age Europe (reported by Mittnik and colleagues), we grouped all reported first-degree related pairs as "sisters" versus non-sisters, and conducted a single test on this 2x2 contingency table. We focused on sisters as we reasoned that sister co-burials (especially adults) could inform us most about sex-biased mobility or burial traditions.

Inferring phenotypic traits and inbreeding for Aşıklı-128

Phenotypic traits

We analyzed a number of functional SNPs (e.g., lactose tolerance, skin pigmentation, eye color) in the high coverage genome of Aşıklı-128. We restricted the analysis to this one individual with the highest coverage (5×) to avoid excessive amounts of missing data. SNPs associated with phenotypes of interest were called from the *BAM* file using the *samtools mpileup* function[143] and filtering with a mapping quality and a base quality lower than 30, as described in van de Loosdrecht et al. (2018).[166] We retrieved the alleles associated with predicting skin, eye and hair color[166] and computed the probability of skin, eye and hair shade for the Aşıklı-128 individual using the tool *HIrisPlex* (http://hirisplex.erasmusmc.nl/). Probabilities of 0.968, 0.019 and 0.009 for having intermediate, very pale and pale skin color were obtained respectively, including a high probability of having light hair color (> 0.99). Regarding the eye color prediction, probabilities of 0.547, 0.338 and 0.115 were obtained for brown, blue and intermediate color, respectively.

We further analyzed derived allele variants in the *MCM6* gene associated with lactose tolerance in Europeans (rs4988235),[167] Africans (rs41456145, rs145946881)[168,169] and Middle Easterners (rs41380347)[168,169] for the Aşıklı-128 individual. Aşıklı-128 shows a homozygous ancestral genotype for these SNP positions, as reported in previous studies that propose the appearance of the lactose tolerance allele much later than the Neolithic.[23,170] Thus, Aşıklı-128 individual was likely lactose intolerant and could not digest milk as an adult.

Runs of homozygosity

We analyzed runs of homozygosity (ROH) in Aşıklı-128, the ancient genome with relatively high genome coverage produced in this study, and also six other relatively high coverage (> 5×) ancient individuals with published data: ZHB (Bon002), Bar8 (M10-106), Loschbour, Stuttgart, NE1 and WC1 (Table Z3), following Kılınç et al.[20] First, we performed diploid genotype calling with DS2 (autosomal transversions data) using *samtools mpileup*,[143] which generated between 1,798,444 and 1,893,648 transversion SNPs for these seven individuals. We estimated the distribution of ROH using *PLINK* (v. 1.9)[156] with the parameters *"–homozyg,–homozyg-window-snp 50,–homozyg-window-het 1,–homolog-windowsthreshold 0.05,–homozyg-snp 50,–homozyg-kb 500,–homozyg-density 50,–homozyg-gap 100"*.

We next calculated the genomic inbreeding coefficient using the F_{ROH} to estimate the level of inbreeding. F_{ROH} measures the individual homozygosity which is the proportion of the genome covered by ROH.[171,172] We calculated F_{ROH} by dividing the summed length of ROH ([3]1.5 Mb) for each individual by the total length of autosomal chromosomes covered by SNPs in megabases. The results are presented in Figure S3G and Table Z15.

Estimating the expected ranges for kinship coefficients using simulations

In order to estimate the expected ranges for the kinship coefficient (θ) and Cotterman coefficients (k_0, k_1, k_2) given noisy data, we performed a set of simulation experiments that we describe here. This is especially important when inferring genetic kinship with ancient genomes due to large and highly variable quantities of missing information. Thus, to investigate the uncertainty in the inferred kinship and Cotterman coefficients based on limited data, we simulated multiple pedigrees (Figure S4C) using both autosomal and X chromosomal data and with realistic degrees of sampling error, and studied the distribution of the aforementioned parameters across each degree of interest in an attempt to approximate their boundaries.

For simulations, we used the modern-day Tuscany individuals (TSI) from the 1000 Genomes phase 3 dataset[152] as a source to generate pedigrees (n = 107). First we extracted both autosomal and X chromosomal SNP data that were genotyped in these Tuscany individuals from the 1000 Genomes Project phased *VCF* files (phase 3).[152] To prepare the autosomal dataset, we filtered SNPs by pruning for linkage disequilibrium with parameters *"–indep-pairwise 100 25 0.4"* and selection those with a minor-allele frequency (MAF) > 0.05 with *"–maf 0.05"* using the *PLINK* tool.[156] We then retained only autosomal SNPs with known sex-specific genetic mapping positions from Bhérer et al.,[173] thus resulting in a total of 157,172 biallelic SNPs. For the X chromosomal dataset, we used *"–maf 0.05"* filtering and removed pseudoautosomal regions from X chromosomes as described in the human reference genome (hs37d5),[174] therefore ensuing a total of 179,716 SNPs. Second, we generated *VCF* files for each pedigree using these autosomal and X chromosomal datasets, using either the *ped-sim* software[175] or our in-house *Python 3.8*[176] library (available at https://github.com/CompEvoMetu/kinshipsim), respectively. Next, we analyzed simulated data using *NgsRelate* software (version 2)[35] to estimate kinship relationships. We finally merged the results generated by *NgsRelate* on the simulated pedigrees to produce the distributions of the kinship statistics. We chose *NgsRelate* for these simulation experiments because its results were generally consistent with the *lcMLkin* and *READ*, and it has the further advantage of being able to co-estimate kinship and inbreeding.[35]

CellPress
OPEN ACCESS

The recombination of genetic information for autosomes was realized via the Housworth and Stahl crossover interference model[177] and the *ped-sim* algorithm (with parameters *"–intf interfere/nu_p_campbell.tsv*[178] *–miss_rate 0–keep_phase"*) using the genetic mapping data as described above. Because the available version of *ped-sim* was designed for autosomal simulations, we simulated the X chromosome using in-house code. The molecule inherited from the mother was generated through the following recombination process: the creation of a gamete, a haploid molecule to be inherited by the offspring, began with the random selection of three points (i.e., SNP locations) on the mother X chromosome -thus dividing it into four random-sized segments. Each segment of the gamete was obtained by randomly selecting that region from one of the two molecules from the parent (i.e., one of the haplotypes in the *VCF*), creating a haploid genotype. While sons only possess the single molecule inherited from the mother, daughters also receive a single-copy X chromosome from their fathers (Figure S4D).

The autosomal investigation focused on first-, second- and third- degree relationships -from which inbred individuals were excluded- as well as unrelated pairs. We generated one hundred independent pedigrees, ensuring sample sizes of 3,500 (first degree), 3,200 (second degree), 2,000 (third degree), 15,600 (unrelated) as well as 700 (siblings), 2,800 (parent-offspring), 800 (half-siblings) and 800 (avuncular, i.e., aunt/uncle-niece/nephew). Before studying properties of their distributions, sample sizes were equalized by random selection. Estimates of θ, k_0, k_1 and k_2 were computed for different SNP sets:

(a) The full set of 157,172 SNPs,

(b) 5,000 randomly chosen SNPs to inform on the lower limit,

(c) Randomly chosen SNPs (smaller than the full set) that match the numbers of overlapping SNPs for ancient individual pairs identified as close relatives in this study: 125,110 for the pair Tepecik-Çiftlik 37-21; 86,247 for the pair Barcın M10_275-M10_271; 80,115 for the pair Barcın L11_216-L11_215; 22,076 for the pair Boncuklu ZHF-ZHJ; 20,810 for the pair Aşıklı 131-136; 18,136 for the pair Boncuklu ZHBJ-ZHAF; 9,151 for the pair Çatalhöyük 2728-2842. Since, the pair Aşıklı 128-133 share 741,193 SNPs, we used the full set of 157,172 SNPs for this Aşıklı pair (Figure S4B).

Since simulating pedigrees starting from *VCF* files is significantly more straightforward than using *BAM* files, we performed *NgsRelate* with *VCF* files, which differs from the real situation where we had used *BAM* files. To account for genotype uncertainty while using *VCF* data we performed simulations using both diploid and pseudo-haploidized datasets.

We then analyzed the distributions of θ, k_0, k_1 and k_2 estimates from *NgsRelate* from the simulated pedigrees. First, we used the 0.025 and 0.975 quantiles of these distributions (two tails of the distributions) to describe the range of expected values for each type of relationship given a certain SNP number, for first-, second-, and third- degrees as well as for parent-offspring, siblings, half-siblings and avuncular relationships.

Differently, for the unrelated case we considered 0.95 as the upper limit (single tail of the distribution, considering zero as the lower edge). The mean value, standard deviation and estimated range for diploid and pseudo-haploidized data are summarized in Table Z16 (kinship coefficient for first-, second, and third- degrees and unrelated cases, number of samples n = 2,000) and Table Z17 (Cotterman coefficients for parent-offspring, siblings, half-siblings and avuncular relationships, n = 700).

The detailed first-degree relationships (e.g., mother-son, father-daughter, sisters) were studied through the θ, k_0, k_1 and k_2 estimates of the X chromosomes computed from two hundred pedigrees. The analysis was performed for the following X chromosomal SNP sets:

(a) The full set of 179,716 SNPs,

(b) 800 randomly chosen SNPs to inform on the lower limit,

(c) Randomly chosen SNPs that match the numbers of overlapping SNPs for ancient individual pairs identified as close relatives in this study: 64,969 for the pair Aşıklı 128-133; 10,654 for the pair Barcın L11_216-L11_215; 9,940 for the pair Tepecik-Çiftlik 37-21; 6,558 for the pair Barcın M10_275-M10_271; 2,653 for the pair Aşıklı 131-136; 1,673 for the pair Boncuklu ZHBJ-ZHAF; 1,538 for the pair Boncuklu ZHF-ZHJ; 814 for the pair Çatalhöyük 2728-2842.

From the resulting distributions, the 95% confidence intervals (ranges) of the coefficients were determined by considering the 0.025 and 0.975 quantiles. The mean value, standard deviation and estimated ranges resulting from the X chromosome simulation are summarized in Table Z18 (θ coefficient, number of samples n = 200).

In the attempt to better characterize mother-daughter and sisters relationships, we also computed the mean and variance of the ratio between autosomal (estimated using 5,000 SNPs) and X chromosomal (estimated using 800 SNPs) θs:

$$\mu\left(\frac{\theta_a}{\theta_x}\right) = \mu(\theta_a) \times \mu\left(\frac{1}{\theta_x}\right)$$

and

$$\sigma^2\left(\frac{\theta_a}{\theta_x}\right) = \mu\left({\theta_a}^2\right) \times \mu\left[\left(\frac{1}{\theta_x}\right)^2\right] - \left[\mu(\theta_a) \times \mu\left(\frac{1}{\theta_x}\right)\right]^2$$

where μ and σ^2 represent mean and variance, respectively, of autosomal θ (θ_a) or X chromosomal θ (θ_x), calculated from the simulation outcomes. The resulting ratio coefficients are summarized in Table Z19. These values were then used to evaluate results shown in Figure 2B.

We note that in Table Z16, the variance in theta estimates shows little dependence on SNP numbers (sample size). We hypothesized that this may be caused by the major contribution of variance in these estimates being the variance in background relatedness among pairs across different simulated pedigrees, rather than sampling error due to differences in randomly chosen SNP sets. In

order to test this idea, we measured the effect of SNP sample size on the variance of our estimates, within the same pedigrees only. Specifically, we generated 10 pedigrees and, for each pedigree, the subsampling process was repeated 10 times for a total of 100 simulations. The average values of the coefficients, together with the standard deviations, were computed for each subsampling and for all couples and relations of interest. Results are summarized in Tables Z20-Z23 for θ, k_0, k_1 and k_2, respectively.

Spatial distances versus genetic distances among burials

We studied the question of whether any *distantly* related pairs of individuals in this dataset, related beyond the 3rd degree, might tend to be buried in the same buildings or at closer proximity, compared to fully unrelated individuals. For this we applied two types of analysis described below. In both analyses we used the outgroup-f_3 statistic between two individuals, calculated as f_3*(YRI, INDV1; INDV2)*, to measure genome-wide genetic similarity, and 1-f_3 to measure genetic distance.

Spatial-genetic distance correlations

We selected specific subsets of individuals from Aşıklı Höyük, Boncuklu Höyük, Çatalhöyük and Barcın Höyük. Table S4 presents the criteria used for selecting the subsets and the individuals excluded from each subset. Namely, in all sites, we included only individuals who were found not closely related, by removing one of each pair of close relatives identified in Figures 2A and 2B, keeping the individual with the higher coverage. We created additional subsets removing individuals who were spatially or temporally distant from the rest (Table S4).

In each subset, for the chosen individual pairs, we created matrices of spatial distance between each pair, calculated as the Euclidean distance between each pair of burials' x and y positions in the site plan. For the same pairs of individuals, we also created genetic distance matrices, using (1-f_3) as measure of distance, where the outgroup f_3-statistic was calculated as f_3*(YRI, INDV1; INDV2)*, with YRI representing the outgroup, and *INDV1* and *INDV2* the genotypes of the individuals studied.

We then calculated the Pearson correlation coefficient between each such pair of spatial and genetic distance matrices, and further calculated a one-sided Mantel test *P*-value using the *"mantel.rtest"* function in the *R* package *"ade4"* (v1.7-13).[179]

Average genetic similarity within buildings

We tested whether individuals buried in or associated with the same building tend to show higher genetic similarity to each other, relative to individuals buried in or associated with other buildings.

This test was performed on the Çatalhöyük and Barcın Höyük data, because these were the only sites with > 1 co-burial clusters. Again, we included only individuals who were found not to be closely related, by removing one of each pair of close relatives identified in Figures 2A and 2B, keeping the individual with the higher coverage.

In Çatalhöyük the data included n = 9 such co-buried individuals associated with Buildings 17, 50, and 114. In Barcın Höyük the data included n = 8 such co-buried individuals associated with Buildings 4, 5, and 14/15.

As a measure of genetic similarity, we used the outgroup f_3, calculated as described above. We first calculated the within-building genetic similarity of all pairs of individuals from each of the 3 buildings, and calculated the mean of these distances. We then created a null distribution of mean co-burial genetic similarities, randomly assigning the 9 or 8 individuals to each building and calculating the mean of within-building similarities, 10,000 times (performed in *R* using the *"sample"* function). A one-sided *P*-value for the alternative hypothesis that within-building genetic similarity would be higher than random was calculated by comparing the observed value with the null.

In both Çatalhöyük and Barcın Höyük, the mean similarity within buildings observed was within the randomly expected distribution. The permutation test *P*-values were calculated as 0.52 and 0.8, respectively.

Current Biology, Volume *31*

Supplemental Information

Variable kinship patterns in Neolithic Anatolia revealed by ancient genomes

Reyhan Yaka, Igor Mapelli, Damla Kaptan, Ayça Doğu, Maciej Chyleński, Ömür Dilek Erdal, Dilek Koptekin, Kıvılcım Başak Vural, Alex Bayliss, Camilla Mazzucato, Evrim Fer, Sevim Seda Çokoğlu, Vendela Kempe Lagerholm, Maja Krzewińska, Cansu Karamurat, Hasan Can Gemici, Arda Sevkar, Nihan Dilşad Dağtaş, Gülşah Merve Kılınç, Donovan Adams, Arielle R. Munters, Ekin Sağlıcan, Marco Milella, Eline M.J. Schotsmans, Erinç Yurtman, Mehmet Çetin, Sevgi Yorulmaz, N. Ezgi Altınışık, Ayshin Ghalichi, Anna Juras, C. Can Bilgin, Torsten Günther, Jan Storå, Mattias Jakobsson, Maurice de Kleijn, Gökhan Mustafaoğlu, Andrew Fairbairn, Jessica Pearson, İnci Togan, Nurcan Kayacan, Arkadiusz Marciniak, Clark Spencer Larsen, Ian Hodder, Çiğdem Atakuman, Marin Pilloud, Elif Sürer, Fokke Gerritsen, Rana Özbal, Douglas Baird, Yılmaz Selim Erdal, Güneş Duru, Mihriban Özbaşaran, Scott D. Haddow, Christopher J. Knüsel, Anders Götherström, Füsun Özer, and Mehmet Somel

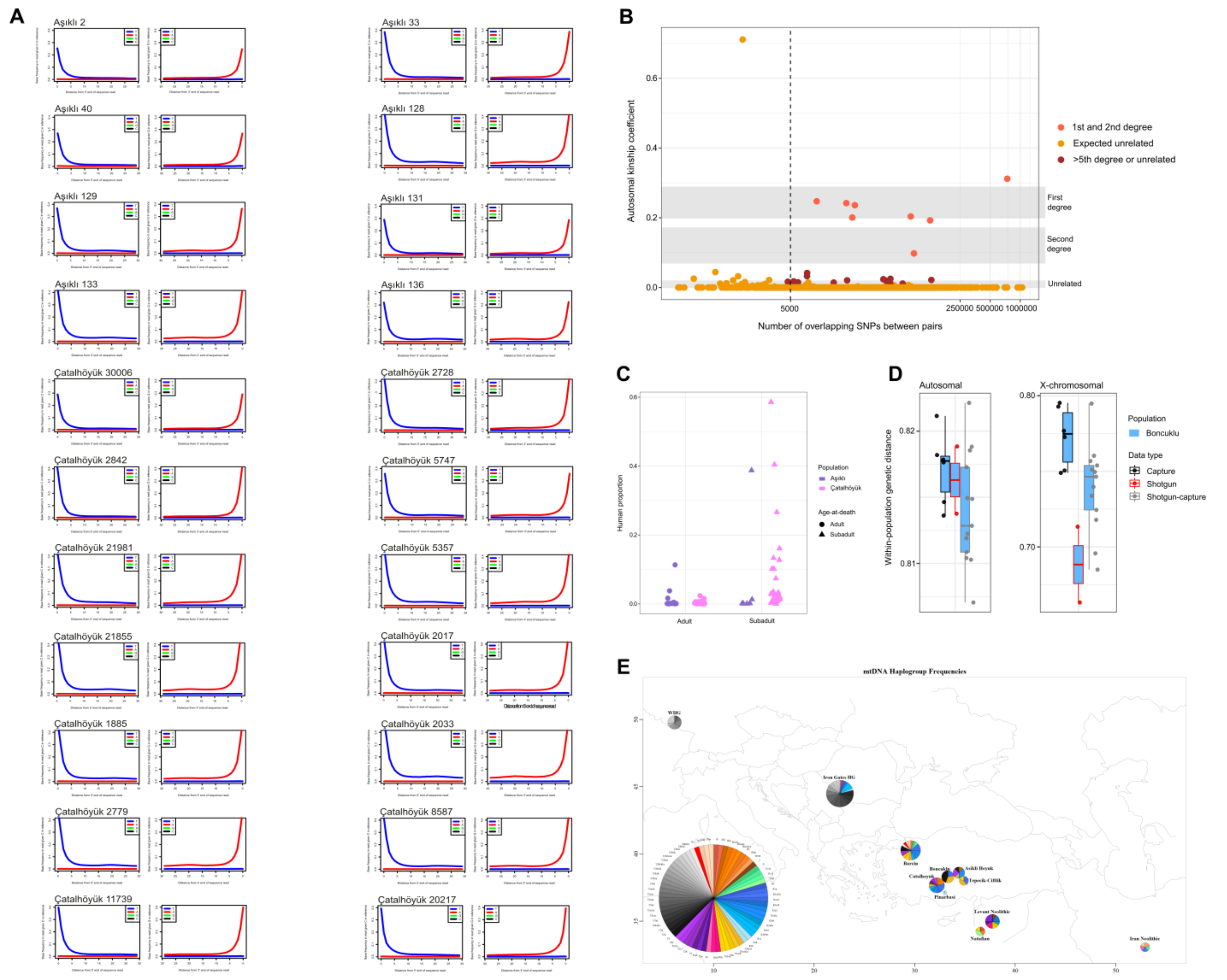

Figure S1. Postmortem damage (deamination) patterns, kinship coefficient (θ) estimates, endogenous human DNA proportions, technical influence on f_3-statistic-based genetic distances and mitochondrial DNA haplogroups, Related to Table 1, Figure 2 and STAR Methods. (A) The graphs show postmortem damage patterns (*PMD*) of all samples from Aşıklı (*n*=8) and Çatalhöyük (*n*=14) as measured by the frequency of transitions at the first 30 positions at the 5' (cytosine to thymine transitions) and 3'ends (guanine to adenine transitions) of the reads. The x-axis indicates the distance from the 5' and 3'ends of the reads and the y-axis indicates the frequency of the *PMDs* (Table Z1). **(B)** Kinship coefficient (θ) estimates vs. overlapping SNP numbers between n=1711 pairs of n=59 Anatolian Neolithic individuals. The x-axis shows the number of overlapping autosomal SNPs between a pair of ancient genomes, and the y-axis shows kinship coefficient estimates for pairs of individuals calculated with autosomal genomic data using *NgsRelate* software (Table Z11). The horizontal gray bars indicate expected θ values based on simulation for first-degree related, second-degree related, and unrelated pairs, from top to bottom, calculated with *NgsRelate* software using haploid data (STAR Methods). The orange points are pairs expected to be unrelated based on their temporal and spatial distances, pink points are those pairs identified as 1st and 2nd degree related from the same site and period, while dark red points are those pairs identified as distantly (>5th degree) or unrelated from the same site and period. The pair with a θ estimate of >0.7 is Aşıklı 2-Aşıklı 40, with c.300 years between them. Based on this result, in order to avoid false positives, a minimum

5,000 overlapping SNPs was chosen as a conservative threshold to assess genetic kinship between pairs. **(C)** Endogenous human DNA proportions in Aşıklı (n=30) and Çatalhöyük (n=60) individuals. Endogenous human DNA proportions in Aşıklı (purple) and Çatalhöyük (pink) individuals' remains were measured as the percentage of shotgun sequenced DNA molecules from prescreening experiments mapping to the reference human genome. Each individual is represented by a single observation. The highest proportion was used for a minority of cases where multiple libraries had been produced per individual. Adult and subadult categories were assigned using osteological age-at-death estimates by the anthropology teams, using standard methods such as human growth, dental calcification and bone maturity [S1]. Endogenous DNA proportions show significant difference between subadult and adult categories in Çatalhöyük (two sided Mann-Whitney U test, $P<0.0001$), but not in Aşıklı ($P=0.70$). **(D)** Technical influence on f_3-statistic-based genetic distances. Distributions of pairwise genetic distances based on outgroup f_3-statistics ($1-f_3$) among Boncuklu individuals, plotted depending on data type, for autosomal SNPs (left) and for X chromosomal SNPs (right). "Capture" indicates pairs of individuals where data were generated using the 1240K SNP capture technology [S2], while "shotgun" indicates pairs of individuals where data were generated using shotgun sequencing enriched with whole genome capture probes [3]; "shotgun-capture" indicates pairs of individuals where one of each pair was produced using either procedure. Although the impact of technical differences is modest for autosomal data, in the comparisons involving X chromosomal SNPs the f_3-statistics-based distance estimates behave differently between the capture and shotgun datasets (despite the overall genetic homogeneity of the Boncuklu population), suggesting the influence of technical effects. **(E)** The geographical distribution of mitochondrial DNA haplogroups of the ancient individuals used in this study. The map displays mitochondrial haplogroup frequencies identified in the early Holocene populations listed in Tables Z1 and Z3. Shades of the same color represent haplogroups categorized under the same major haplogroup, as indicated in the key. Size of the pies are proportional to the sample size. Pie charts were plotted based on the approximate coordinates of archaeological sites and regions. WHG: Western European hunter-gatherers. The map was drawn using the *mapplots* package version 1.5.1 [4] in *R*.

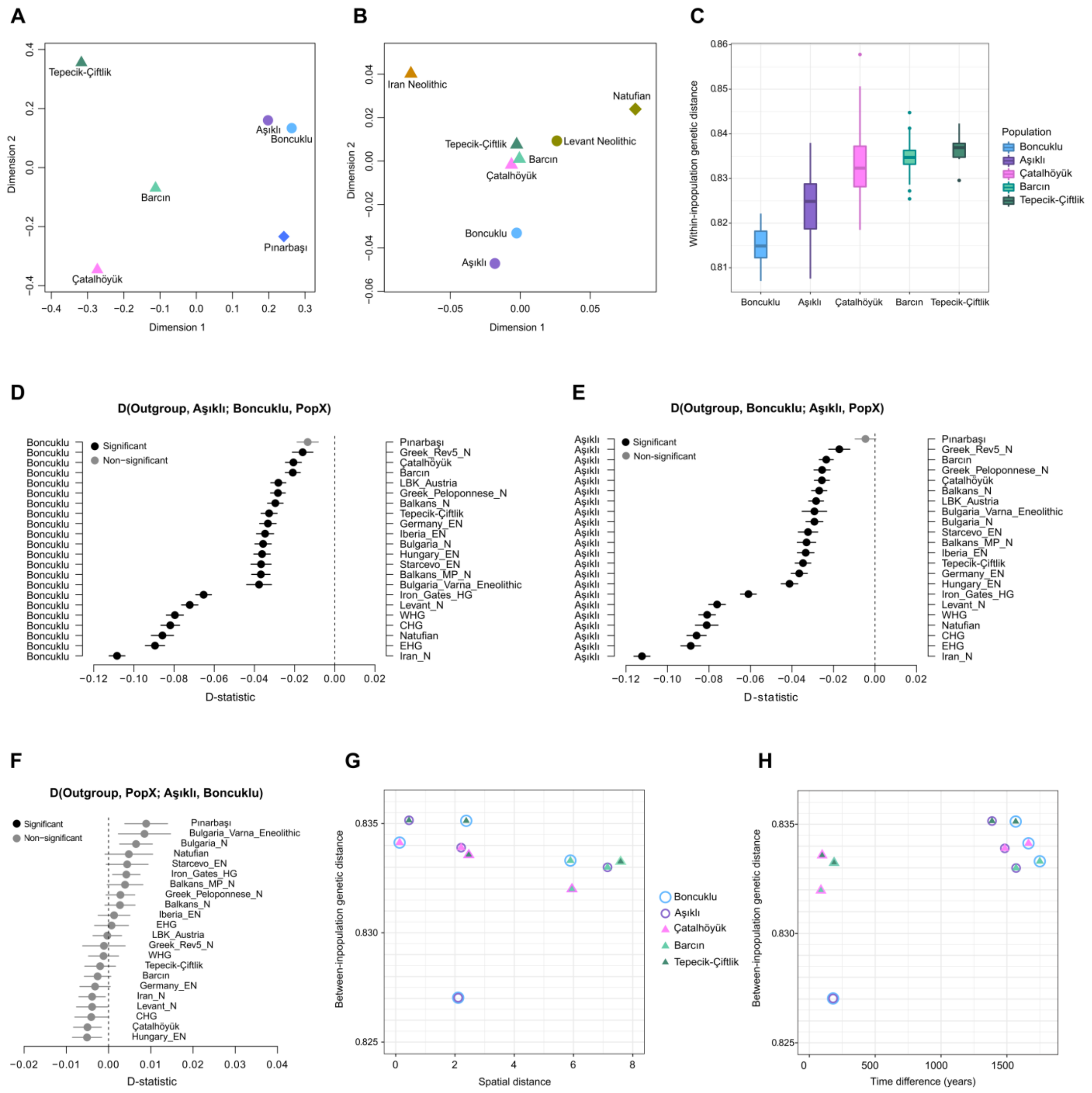

Figure S2. Genome-wide similarity patterns among ancient populations, Related to Figure 1. (**A**) Population level multidimensional scaling (MDS) plot summarizing genetic distances based on outgroup f_3-statistics calculated between populations from Anatolia using population genotype data (goodness of fit r^2=0.8). (**B**) Population level MDS plot summarizing genetic distances based on F_{ST} calculated between populations from Anatolia, Levant and Iran using population genotype data (goodness of fit r^2=0.95). **(C)** Distribution of within-population genetic distances for each population based on outgroup f_3-statistics. The whiskers extend to the most extreme data point ≤1.5 times the interquartile range. (**D**) Population level D-statistics for the topology *D(Yoruba, Aşıklı; Boncuklu, PopX)* where Boncuklu is indicated on the left-hand y-axis and *PopX* stands for ancient individual/populations from Anatolian, Near Eastern, Balkans and European farmer and hunter-gatherer groups, indicated on the right-hand y-axis. $D < 0$ shows higher genetic affinity between the test population (name indicated on the top, Aşıklı) and

Boncuklu, and $D > 0$ shows higher genetic affinity between the test population and the second individual/population as *PopX*. In each comparison, black color indicates nominally significant *D*-statistics (*i.e.,* not corrected for multiple testing) with $|Z| \geq 3$ and grey-color shows non-significant values. Horizontal lines show +/- one standard error. See Table Z3 for population details. (**E**) Population level *D*-statistic for the topology *D(Yoruba, Boncuklu; Aşıklı, PopX)* where Aşıklı is indicated on the left-hand y-axis and *PopX* stands for ancient individual/populations from Anatolian, Near Eastern, Balkans and European farmer and hunter-gatherer groups, indicated on the right-hand y-axis. $D < 0$ shows higher genetic affinity between the test population (name indicated on the top, Boncuklu) and Aşıklı, and $D > 0$ shows higher genetic affinity between the test population and the second individual/population as *PopX*. See Table Z3 for population details. (**F**) Population level *D*-statistics for the topology *D(Yoruba, PopX; Aşıklı, Boncuklu)* where Aşıklı and Boncuklu are indicated on the left- and right-hand y-axis, respectively, and *PopX* stands for ancient individual/populations from Anatolian, Near Eastern, Balkans and European farmer and hunter-gatherer groups, indicated in the middle. $D < 0$ shows higher genetic affinity between the test population (*PopX*) and Aşıklı, and $D > 0$ shows higher genetic affinity between the test population (*PopX*) and Boncuklu. See Table Z3 for population details. (**G-H**) Genetic distance between populations compared with their spatial and chronological differences. The plots show genetic distance vs. spatial distance (**G**) and genetic distance vs. time difference (**H**) between pairs of Neolithic Anatolian settlements. Each pair is denoted by the combination of symbols of both sites, shown in the key. For each pair of sites, a genetic distance matrix was created based on outgroup f_3-statistics using $(1-f_3)$. Matrices of spatial distance and time distance between the same pairs were calculated as the Euclidean distance between their longitude and latitude, and the difference between the ages of the settlements (mid-point of the occupation period), respectively. Overall, both time and space explain non-significant genetic variation among across these five sites (one-sided Mantel test $P>0.8$ and $P>0.4$, respectively).

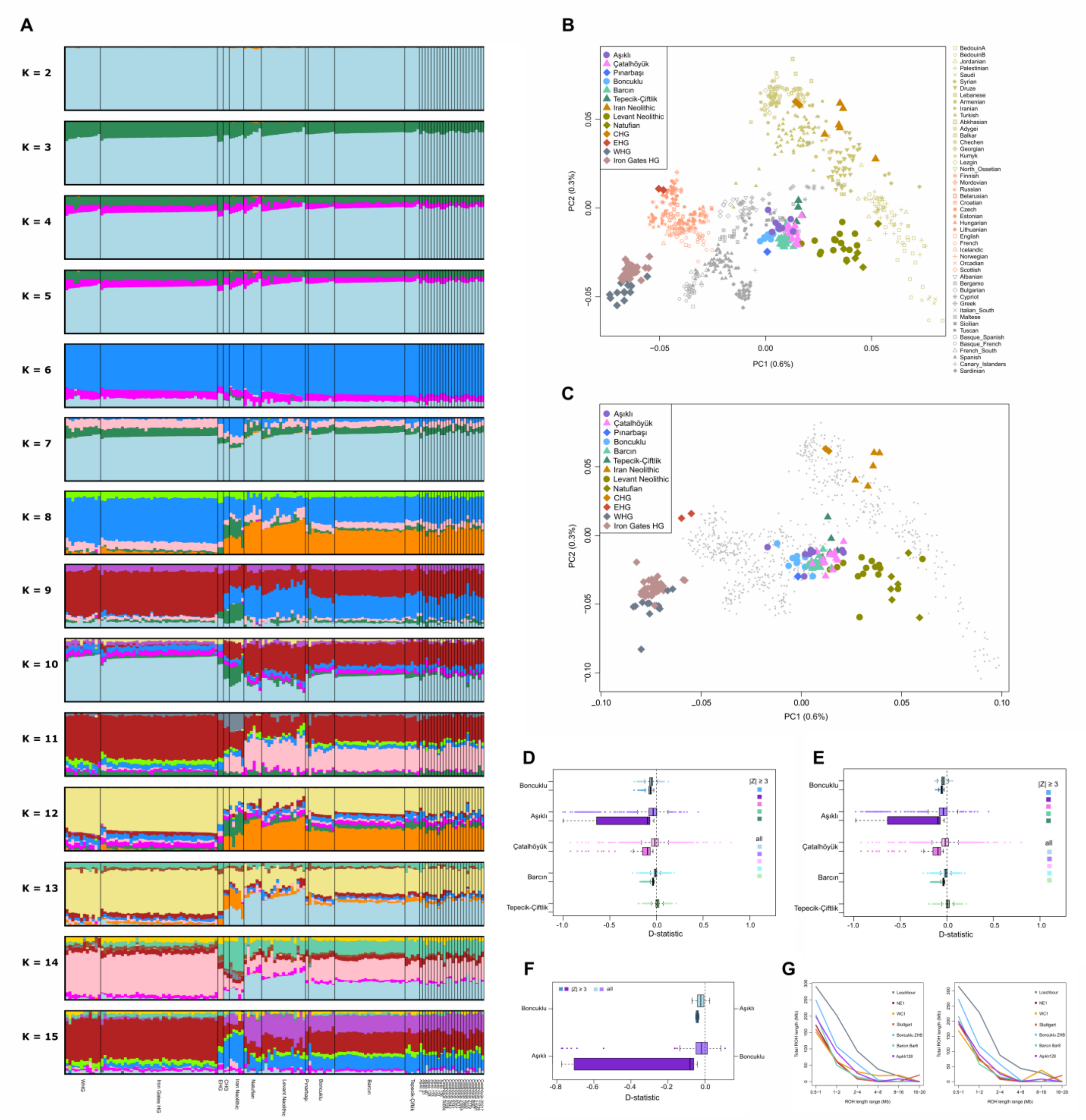

Figure S3. ***ADMIXTURE*** **analysis, principal components analysis (PCA), individual-level** ***D*****-statistics results, and runs of homozygosity (ROH), Related to Figure 1. (A)** *ADMIXTURE* analysis results for *K*=2 to *K*=15 ancestry components for ancient individuals are shown as bar charts. The individuals included in each population sample are listed in Tables Z2 and Z3. We performed unsupervised *ADMIXTURE* analysis using n=231 present-day populations from the Human Origins dataset [5,6] and ancient individuals were projected on the calculated components (STAR Methods). **(B)** PCA with ancient individuals from Anatolia including published ancient and present-day individuals from West Eurasian populations. The individuals included in each population sample are listed in Tables Z2 and Z3. Each ancient individual's genotype was projected upon the first two principal components calculated using n=49 West Eurasian present-day populations from the Human Origins dataset [5,6] (the percentages of

variance explained are shown in parentheses). Colored dots represent different ancient and modern-day individuals as shown in the key. **(C)** PCA plot describing genetic similarities as in panel B, but using only n=110,933 transversion SNPs and n=49 West Eurasian present-day populations from the Human Origins dataset [5,6]. Also, for simplicity we did not color present-day populations in this figure. **(D)** Distributions of individual-level *D*-statistics involving comparisons among and within Anatolian Neolithic settlements. Negative *D*-statistics indicate an individual was genetically closer to another from her/his own settlement (shown on the left-hand side labels), than to an individual from one of the other four settlements, in *D*-tests of the form *D(Outgroup, X1; X2, X3)*, where *X1* and *X2* are from the same, and *X3* from a different Anatolian Neolithic settlement. Test results that are nominally significant ($|Z|>3$, darker colors) and all results (lighter colors) are shown as separate boxplots. In total, 22,638 *D*-tests were conducted and 84-93% of 576-11,780 comparisons per site were significant, except for Tepecik-Çiftlik. In the latter, none of the test results were nominally significant. In all analyses we removed all but one individual where clusters of genetic relatives were identified. **(E)** Distributions of individual-level *D*-statistics as in panel D, but only using shotgun data for Boncuklu, to avoid the influence of technical biases that may arise due to use of capture and shotgun data. The results are qualitatively the same as in panel D. **(F)** Distributions of individual-level *D*-statistics as in panel D for Aşıklı vs Boncuklu comparisons only using shotgun data for Boncuklu to avoid the influence of technical biases that may arise due to use of capture and shotgun data. In total, 156 *D*-tests were conducted and 11-13% of 36-120 comparisons per site were significant. **(G)** Runs of homozygosity (ROH) in seven ancient early Holocene West Eurasian genomes. The left panel shows ROH length distributions for Loschbour (European Mesolithic), NE1 (Hungary Neolithic), WC1 (Iran Neolithic), Stuttgart (early European Neolithic), Boncuklu ZHB (Bon002) (Anatolian Aceramic), Barcın Bar8 (M10-106) (Anatolian Ceramic Neolithic), Aşıklı 128 (Anatolian Aceramic) and in the right panel ROH length distributions with each genome subsampled down to 5× coverage. Estimated F_{ROH} values per genome are presented in Table Z15.

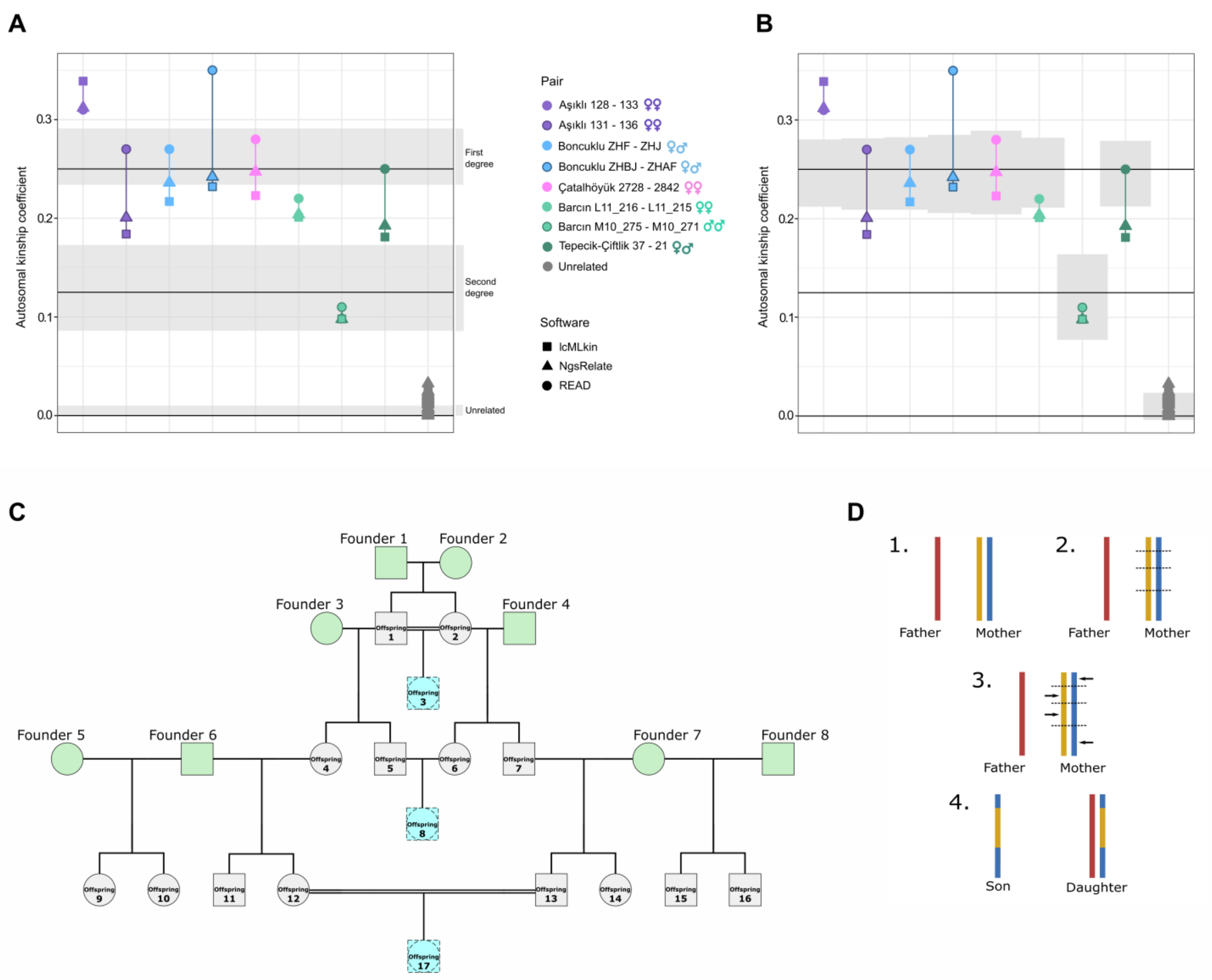

Figure S4. Kinship coefficients (*θ*), the pedigree used in kinship simulations and simulation of X chromosomal meiosis, Related to Figure 2. (A-B) Kinship coefficients (*θ*) between related pairs of individuals calculated using autosomal genomic data and three different software, shown as different symbols. The horizontal black lines indicate expected autosomal *θ* values for first-degree related, second-degree related, and unrelated pairs (0.25, 0.125 and 0, respectively). The horizontal gray bars indicate expected *θ* values for first-degree related, second-degree related, and unrelated pairs, from top to bottom, calculated with *NgsRelate* software on one hundred simulated pedigrees, which resulted in 2,000 samples for each group (STAR Methods). Whereas first-, second- and third- degree ranges were defined by the 0.025 and 0.975 quantiles, the unrelated group had an upper boundary identified by the 0.95 quantile, as we considered zero to be its lower limit. The left panel (**A**) shows the ranges for diploid data with 5,000 SNPs – [0.2357, 0.2882], [0.0919, 0.1681], [0.0304, 0.1187], [0.0000, 0.0095] for first-, second-, third- (not in the figure) degrees and unrelated pairs respectively (the difference from Figure 2A is that the latter shows ranges calculated using pseudo-haploidized data) (Table Z16). In the right panel (**B**) the ranges were determined using haploidized data and the same number of SNPs each pair shared, i.e., 157,172 SNPs for Aşıklı 128-133 resulting in a first-degree range of [0.2201, 0.2760], 20,810 for Aşıklı 131-136 and an interval for the first degree equal to [0.2155, 0.2765], 22,076 for Boncuklu ZHF-ZHJ resulting in a first-degree range of [0.2142, 0.2790], 18,136 for Boncuklu ZHBJ-ZHAF for an estimated first-degree interval equal to [0.2148, 0.2771], 9,151 for

Çatalhöyük 2728-2842 and first-degree range of [0.2118, 0.2827], 80,115 for Barcın L11_216-L11_215 producing a first-degree interval of [0.2197, 0.2763], 86,247 for Barcın M10_275-M10_271 and a second-degree range of [0.0852, 0.1586], 125,110 for Tepecik-Çiftlik 37-21 with a first-degree range of [0.2204, 0.2742]. For the unrelated group we considered 5,000 SNPs which resulted in an interval of [0.0000, 0.0210] (Table Z16). Since Aşıklı 128-133 shared 741,193 SNPs, we used the full set of 157,172 SNPs for this Aşıklı pair. **(C)** The light green-colored elements (delimited by a solid line) represent the founders who were randomly selected from a genomic polymorphism dataset of unrelated modern Tuscany individuals from 1000 Genomes phase 3 dataset [7]. All individuals in the pedigree have a predefined sex (circle for female and square for male), the only exception being the inbred offspring (light blue-colored elements surrounded by dashed lines) whose sex was uniformly randomized across the generated pedigrees. (**D**) The X chromosome recombination process in the mother but not in the father. A pair of X chromosomes in the mother (**1**) are each randomly broken at 3 recombination (crossover) points (**2**), and the resulting 4 segments are randomly switched between each pair of X chromosomes (**3**). A son solely inherits one of the resulting mother's recombinant gamete, whereas a daughter inherits also the original X chromosome of the father (**4**).

Site	Individuals studied	Contemporaneous pairs	Co-buried pairs	First (second) degree related pairs	Kin pairs among co-burials %	Co-buried individuals	Genetic kin	Kin among co-burials %
Aşıklı	8	10	10	2 (0)	20 (2/10)	5	4	80 (4/5)
Boncuklu	9	36	10	2 (0)	20 (2/10)	5	4	80 (4/5)
Çatalhöyük	14	66	17	1 (0)	6 (1/17)	10	2	20 (2/10)
Barcın	23	107	12	1 (1)	16 (2/12)	10	4	40 (4/10)
Tepecik-Çiftlik	5	4	1	1 (0)	100 (1/1)	2	2	100 (2/2)
TOTAL	59	223	50	7 (1)	-	32	16	-

Table S1. Frequencies of individuals and individual pairs studied for genetic kinship, Related to Figure 2 and STAR Methods.
The column titled "Contemporaneous pairs" shows the numbers of all pairs of individuals from the same or similar archaeological levels and thus could be potentially closely related. "Co-buried pairs" shows the number of pairs of individuals in co-burial clusters, associated with (*i.e.,* buried inside or next to) the same or proximate buildings, shown in detail in Figure 3. "First (second) degree related pairs" shows the number of first-degree (and in parentheses, second-degree) related pairs identified in this study. "Kin pairs among co-burials %" shows the percentage of co-buried pairs identified as genetic kin, i.e. first- or second-degree (and the actual numbers in parentheses). "Co-buried individuals" shows the number of individuals' whose remains were associated with a building that included at least one other individual genetically studied here. "Kin among co-burials %" shows the percentage of co-buried individuals with a first- or second-degree relative identified in this study (and the actual numbers in parentheses). All genetic kin identified were individuals who were part of co-burials clusters. No pairs with third degree relatedness were identified here. A Fisher's exact test performed by comparing the frequencies of individuals in co-burials with relatives in Aşıklı and Boncuklu (n=8) versus Çatalhöyük and Barcın Höyük (n=6), and without relatives in the same pairs of sites (n=2 and n=14, respectively), reveals a significant difference (odds ratio=8.6, Fisher's exact test P=0.019). When including Tepecik-Çiftlik data to the Ceramic Neolithic group, we also find a marginal difference between Aceramic and Ceramic period frequencies (odds ratio=6.6, Fisher's exact test P=0.054).

Kin-relationship	Autosomal θ	chrX θ	k_0	k_1	k_2
Mother-daughter	0.25	0.25	0	1	0
Mother-son	0.25	0.5	0	1	0
Father-daughter	0.25	0.5	0	1	0
Father-son	0.25	0	0	1	0
Sisters	0.25	0.375	0.25	0.5	0.25
Brothers	0.25	0.5	0.25	0.5	0.25
Brother-sister	0.25	0.25	0.25	0.5	0.25

Table S2. Expected autosomal and X chromosomal (chrX) kinship coefficients (θ) and Cotterman coefficients for first-degree genetic relatives, Related to Figure 2 and STAR Methods.

Cotterman coefficients (k_0, k_1, k_2) define probabilities of sharing 0, 1 or 2 alleles identical-by-descent between a pair of individuals. The values assume no inbreeding in the pedigree.

Pair	Most likely pedigree relationship	Autosomal θ	chr X θ	Autosomal Cotterman coefficients			MtDNA haplogroup	chrY haplogroup	Osteological age-at-death	No. of SNPs	C14 date (95% cal BCE)
				k_0	k_1	k_2					
Aşıklı 128-133	Sisters	0.31	0.25	0.15	0.45	0.40	Same	-	Child and old adult	741,193	8225–7955<br>8170-7735
	We inferred this pair as likely sisters (as opposed to mother-daughter) due to their low k_1 values, although their low X chromosomal θ could also be consistent with a mother-daughter pair. Notably, the Çatalhöyük and Barcın sister pairs also display similar statistics. They may have been contemporary based on their radiocarbon data.										
Aşıklı 131-136	Sisters	0.20	0.17	0.46	0.28	0.25	Same	-	Child and adult	20,810	8200-7740<br>8175-7655
	We inferred this pair as likely sisters (as opposed to mother-daughter) due to their low k_1 values, although their low X chromosomal θ could also be consistent with a mother-daughter pair. Notably, the Çatalhöyük and Barcın sister pairs also display similar statistics. They may have been contemporary based on their radiocarbon data.										
Boncuklu ZHJ-ZHF	Mother-son	0.24	0.43	0.22	0.62	0.13	Same	-	Old adult and adult	22,076	8295-8240<br>8225-7940
	We inferred this pair as parent-offspring (as opposed to brother-sister) due to their relatively high X chromosomal θ, their relatively high autosomal k_1 values and their age-at-death and C14 dates. A mother-son relationship is more likely than a father-daughter relationship given mtDNA haplogroup sharing (Table Z14) and their age-at-death and C14 dates (Table Z2). Their radiocarbon data suggests that it is 90% probable that the woman (ZHJ) died first.										
Boncuklu ZHBJ-ZHAF	Brother-sister	0.24	0.28	0.18	0.67	0.10	Same	-	Middle adults	18,136	>7952<br>8285-8010
	We inferred this pair as likely brother-sister (as opposed to parent-offspring) due to their low X chromosomal θ, but their relatively high autosomal k_1 could also be consistent with a mother-son pair. A father-daughter pair is also possible but even less likely due to mtDNA haplogroup sharing. They may have been contemporary based on their radiocarbon data.										
Çatalhöyük 2728-2842	Sisters	0.25	0.23	0.49	0.03	0.48	Same	-	Infant and child	9,151	6695-6505<br>6690-6505
	We inferred this pair as sisters (as opposed to mother-daughter) due to their subadult age. Their radiocarbon data also suggests they may have been contemporary.										

Barcın L11_216-L11_215	Sisters	0.20	0.28	0.37	0.44	0.19	Same	-	Both infants	80,115	c.6200-6100 6320-6080
	We inferred this pair as sisters (as opposed to mother-daughter) due to their subadult age. Their radiocarbon data also suggests they may have been contemporary.										
Barcın M10_275 - M10_271	Paternal half-siblings or uncle-nephew	0.1	0.02	0.61	0.39	0.0	Different	Same	Both infants	86,247	6405-6240 6425-6250
	We inferred this pair as paternal half-siblings or an uncle-nephew pair (as opposed to maternal half-siblings or a grandparent-grandchild pair) due to their sharing of Y chromosomal but not mtDNA haplogroups and their young age. A third-degree relationship (paternal cousins) is also possible. Their radiocarbon data suggests they may have been contemporary.										
Tepecik-Çiftlik 37-21	Mother-son	0.19	0.41	0.23	0.77	0.00	Same	-	Adult and old adult	125,110	6385-6100 6225-6070
	We inferred this pair as likely mother-son (as opposed to brother-sister) due to their high X chromosomal θ, their high autosomal k_1 value and their C14 dates, although a father-daughter pair is also possible, but less likely. Their radiocarbon data also suggests that it is 96% probable that the woman (Individual 37) died first.										

Table S3. Combined evidence for pedigree relationships for related pairs and its evaluation, Related to Figure 2 and STAR Methods.

Cotterman coefficients (k_0, k_1, k_2) define probabilities of sharing 0, 1 or 2 alleles identical-by-descent between a pair of individuals. The "Osteological age" and "C14 date" columns show the information for each individual in each pair in the same order as listed in the "Pair" column. Expected values for the mentioned statistics are presented in Table S2. For each pair, we provide brief discussion on how the most likely relationship was inferred.

Site	*n*	*ρ*	*P*-value	Sample criteria	Individuals excluded (justification)
Çatalhöyük	13	-0.0402	0.62	No relatives	2728 (with relatives)
Çatalhöyük	11	-0.0014	0.49	No relatives and only mid-7th mil. BCE	2728 (with relatives), 11739 and 20217 (Final Çatalhöyük layers)
Çatalhöyük	9	-0.1086	0.77	No relatives and only levels South K,M,N	2728 (with relatives), 11739 and 20217 (Final Çatalhöyük layers)
Barcın	21	-0.0857	0.76	No relatives	L11-215 and M10-271 (with relatives)
Barcın	13	0.0219	0.36	No relatives and only Layers c and d	L11-215 and M10-271 (with relatives), L11_322, M13_72, M11_59, M10_106, L11_213, L11_439, M13_170, L11_216 (from Layers a and b)
Aşıklı	6	-0.3035	0.65	No relatives	Ash131 and Ash133 (with relatives)
Aşıklı	5	0.0191	0.42	No relatives and spatial proximity	Ash131 and Ash133 (with relatives), Ash002 (spatially an outlier)
Boncuklu	7	0.0348	0.45	No relatives	ZHAF and ZHJ (with relatives)

Table S4. Correlation between genetic and spatial distances across burials, Related to STAR Methods.

The table shows the number of individuals (*n*), Pearson correlation coefficient (*ρ*) and Mantel test *P*-values (one-sided) per test. The column “Sample criteria” describes what criteria were chosen to include individuals in the analysis, where “no relatives” indicates one individual from each pair of close relatives was removed. The column “Individuals excluded” lists the IDs of individuals excluded from the analysis and the justification in parentheses.

Supplemental References:

S1. Buikstra, J.E., and Ubelaker, D.H. (1994). Standards for Data Collection from Human Skeletal Remains : Proceedings of a Seminar at the Field Museum of Natural History J. E. Buikstra, ed. (Arkansas Archeological Survey).

S2. Feldman, M., Fernández-Domínguez, E., Reynolds, L., Baird, D., Pearson, J., Hershkovitz, I., May, H., Goring-Morris, N., Benz, M., Gresky, J., et al. (2019). Late Pleistocene human genome suggests a local origin for the first farmers of central Anatolia. Nat. Commun. *10*, 1–10. 10.1038/s41467-019-09209-7.

S3. Kılınç, G.M., Omrak, A., Özer, F., Günther, T., Büyükkarakaya, A.M., Bıçakçı, E., Baird, D., Dönertaş, H.M., Ghalichi, A., Yaka, R., et al. (2016). The Demographic Development of the First Farmers in Anatolia. Curr. Biol. *26*, 2659–2666. 10.1016/j.cub.2016.07.057.

S4. Gerritsen, H. (2018). mapplots: Data Visualisation on Maps. R package version 1.5.1. https://cran.r-project.org/package=mapplots.

S5. Lazaridis, I., Patterson, N., Mittnik, A., Renaud, G., Mallick, S., Kirsanow, K., Sudmant, P.H., Schraiber, J.G., Castellano, S., Lipson, M., et al. (2014). Ancient human genomes suggest three ancestral populations for present-day Europeans. Nature *513*, 409–413. 10.1038/nature13673.

S6. Patterson, N., Moorjani, P., Luo, Y., Mallick, S., Rohland, N., Zhan, Y., Genschoreck, T., Webster, T., and Reich, D. (2012). Ancient admixture in human history. Genetics *192*, 1065–1093. 10.1534/genetics.112.145037.

S7. Auton, A., Abecasis, G.R., Altshuler, D.M., Durbin, R.M., Bentley, D.R., Chakravarti, A., Clark, A.G., Donnelly, P., Eichler, E.E., Flicek, P., et al. (2015). A global reference for human genetic variation. Nature *526*, 68–74. 10.1038/nature15393.
